# A Network-Based Method to Assess the Statistical Significance of Mild Co-Regulation Effects

**DOI:** 10.1371/journal.pone.0073413

**Published:** 2013-09-09

**Authors:** Emőke-Ágnes Horvát, Jitao David Zhang, Stefan Uhlmann, Özgür Sahin, Katharina Anna Zweig

**Affiliations:** 1 Interdisciplinary Center for Scientific Computing, University of Heidelberg, Heidelberg, Germany; 2 Network Analysis and Graph Theory, Technical University of Kaiserslautern, Kaiserslautern, Germany; 3 Computational Biology and Bioinformatics, Pharmaceutical Research and Early Development, Basel, Switzerland; 4 Division of Molecular Genome Analysis, German Cancer Research Institute, Heidelberg, Germany; 5 Department of Molecular and Cellular Oncology, The University of Texas MD Anderson Cancer Center, Houston, Texas, United States of America; Niels Bohr Institute, Denmark

## Abstract

Recent development of high-throughput, multiplexing technology has initiated projects that systematically investigate interactions between two types of components in biological networks, for instance transcription factors and promoter sequences, or microRNAs (miRNAs) and mRNAs. In terms of network biology, such screening approaches primarily attempt to elucidate relations between biological components of two distinct types, which can be represented as edges between nodes in a bipartite graph. However, it is often desirable not only to determine regulatory relationships between nodes of different types, but also to understand the connection patterns of nodes of the same type. Especially interesting is the co-occurrence of two nodes of the same type, i.e., the number of their common neighbours, which current high-throughput screening analysis fails to address. The co-occurrence gives the number of circumstances under which both of the biological components are influenced in the same way. Here we present SICORE, a novel network-based method to detect pairs of nodes with a statistically significant co-occurrence. We first show the stability of the proposed method on artificial data sets: when randomly adding and deleting observations we obtain reliable results even with noise exceeding the expected level in large-scale experiments. Subsequently, we illustrate the viability of the method based on the analysis of a proteomic screening data set to reveal regulatory patterns of human microRNAs targeting proteins in the EGFR-driven cell cycle signalling system. Since statistically significant co-occurrence may indicate functional synergy and the mechanisms underlying canalization, and thus hold promise in drug target identification and therapeutic development, we provide a platform-independent implementation of SICORE with a graphical user interface as a novel tool in the arsenal of high-throughput screening analysis.

## Introduction

High-throughput screening is a well-established tool for large-scale experiments since it provides an overview of how different cellular variables change under various conditions. Such experiments monitor for instance the alteration of protein levels due to different transcription factors and changed environmental conditions like starvation or enhanced radiation [1]. Biological or chemical perturbations that specifically influence single gene expression, including small interference RNAs (siRNAs) or microRNAs (miRNAs), have been coupled with protein assays to systematically study the relationship between gene expression and function [2]. miRNAs are a large class of small non-protein-coding RNAs that usually (but not exclusively [3]) function as negative regulators. It is known that they play an essential role in the development and maintenance of many diseases: for example, they are tumour suppressors or oncogenes (oncomirs) in various types of cancer [4]–[10]. There are slightly more than 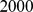 mature human miRNAs registered in the miRBase release 19 [11], [12] and these may target over 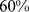 of the mammalian genes [13] whose corresponding proteins can display diverse functions.

Until recently, large-scale experiments designed to investigate regulatory relationships between miRNAs and protein-coding genes have either studied one or few miRNAs against a large number of genes (on the transcriptomic [14] or the proteomic [15], [16] level), or tested a library of miRNA mimics or inhibitors against one or few genes [17]. In either approach, univariate analysis prevalent in high-throughput analysis [18] has been frequently applied to rank targets or perturbations, e.g., by 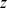-score or 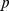-value, in order to interpret the results. It is known that large-scale experiments often come with the trade-off that not all of the results are very reliable [19]: the preparation of the cells and tissues, variances in the chip, detection mediated by antibodies, and sensors that quantify signals are all independent sources of noise. To avoid false-positive results, a strict threshold on these values assures that only those effects are reported that have a low probability to be caused by random or non-functional fluctuation around the resting level, e.g., due to handling or measuring errors. It has however been confirmed that many of the protein regulating effects of the whole human genome miRNA (miRome) are mild [15], [16], [20]. These mild effects can only be detected if observations with a low significance are also included in the analysis, which in turn increases false-positive results. This problem of detecting mild regulation effects was the motivation behind a novel computational approach: as we show in this article, it is computationally feasible to determine whether the number of shared co-regulation conditions of two proteins or protein-regulating conditions is statistically significant or not. The proposed method helps to find groups of proteins that are significantly co-regulated by the same set of miRNAs (or groups of miRNAs that co-regulate the same set of proteins). The implication is then that if two proteins are co-regulated by a significant number of regulating conditions, these regulation effects have a higher chance to be true-positive regulating effects than their respective 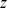-scores suggest. Furthermore, by identifying pairs of proteins that are significantly co-regulated, experimentalists can make hypotheses of functional relationships following the *guilt-by-association* principle [21], [22].

In this article, we present the SIgnificant CO-REgulation filter algorithm (SICORE) and give details needed for transferring it to other applications. For instance, we discuss when to use the method (noisy data containing mild effects) and which decisions need to be made when applying it (especially concerning the choice of meaningful significance thresholds). The algorithm was motivated by the specific biological question raised by the high-throughput study described in Uhlman et al. [20]: How to map regulatory network structures in the EGFR-driven signalling system modulated by human miRNAs? In that paper, we briefly presented SICORE, showed the protein co-regulation network identified by it and provided experimental validation for several of the obtained predictions. Besides determining co-regulation patterns, the framework is generally applicable to any biological data set containing two types of entities that interact with each other. In network terms, the data set must have a bipartite structure. In the following, the *co-occurrence* of two nodes of the same type will be defined as the number of common neighbours they share in this network. With this, the method proceeds in three steps:

1. Given a pair of nodes, the number of their co-occurrences is counted.
2. Then, the probability that at least this number of co-occurrences appears in an appropriate null model is computed.
3. A proper significance level is chosen. Based on it, the null hypothesis is accepted or rejected.

The main feature of the proposed method is its robustness against noise, which we demonstrate here on artificial data sets that emulate a possible biological structure. The advantage of artificial data sets is that they can be constructed in such a way that the *gold standard* (the true positive and negative results to be found by an optimal algorithm) can easily be determined. We show that SICORE is robust against random elimination and random addition of observations, which models two typical sources of noise in biological data. Furthermore, we analyse a real data set between all known human miRNAs (miRome) and a subset of proteins in the EGFR-driven signalling system in an *in vitro* model of human breast cancer. While the results for protein co-regulation have been reported in Uhlman et al. [20], here we provide key features of co-regulated miRNAs for the first time and discuss the general applicability of the method. Finally, we provide an open-source software implementation of SICORE available under a GPL licence at cna.cs.uni-kl.de/SICOP.

## Materials and Methods

### Biological Data used in this Paper

Cells from the human breast cancer line MDA-MB-231 are transfected with a library of 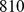 miRNA mimics (miRIDIAN, Dharmacon) listed in Table S1 and the level of 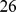 different proteins from the EGFR-signalling pathway (Table S2) is measured on reverse phase protein arrays (RPPA) with carefully selected antibodies. Normalized signals are transformed to a 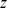-score for pairs consisting of one miRNA and one protein [18], [20]. Images of RPPA are analysed with the GenePix software. The light signal is log2 transformed after removing the background using neighbourhood pixels. Block effects are removed by fitting transformed values to a one-way ANOVA model incorporating blocks of protein arrays. Normalization of signals with respect to input protein concentration is performed with an adapted linear model from [23] allowing for polynomial fitting. A positive 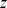-score signifies that the protein’s level was higher than its mean, while a negative 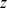-score indicates that it was lower. The array of normalized 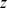-scores then quantifies the change in the gene expression level with regard to the protein’s resting level, building the basis for the following analysis.

The data obtained in this way is not without limitations. For instance, the used experimental methodologies (transfection of cells with a miRNA library and reverse phase protein arrays) can only be applied in a population-based manner. Thus, cell-to-cell variability is disregarded and only mechanisms involving most of the cells in the population are identified.

### Regulation Graphs: Building a Bipartite Graph Model from Protein Array Data

**Figure 1:**
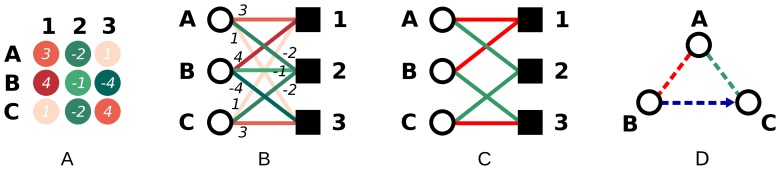
Converting the normalized *z*-score array into a bipartite graph and illustrating the co-regulation patterns of interest. (A) Exemplary array depicting the normalized 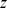-scores of the change in expression level for proteins 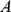, 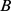, and 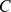 when cells are transfected with miRNAs 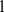, 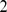, and 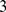. The 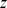-scores are specified as white labels. (B) The corresponding bipartite graph where 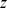-scores are represented by weighted edges; the weights are shown as labels on the edges. (C) After applying a threshold 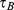 to the weights, only some relationships are retained. In this case 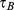 equals 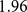, corresponding to a 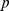-value of 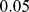. Edges with a positive weight (up-regulation) are shown in red, edges with a negative weight (down-regulation) in green. (D) Protein co-regulation graph based on the co-regulation patterns as described in the text. Colours denote the co-regulation pattern: the red edge denotes co-up-regulation, the green edge denotes co-down-regulation; the blue, directed edge from 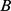 to 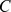 indicates that 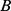 is down-regulated while 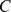 is up-regulated by the same miRNA.

The processed protein array data consists of a 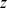-score for each pair of miRNA and protein. We determine a hard threshold 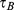 to build the basic bipartite graph. Given the data and the threshold 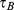, the bipartite graph model contains an edge between any pair of miRNA and protein if the absolute value of the corresponding observed 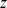-score is at least as large as 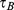. Note that these edges are unweighted, i.e., all of the edges are treated equally after this step, regardless of the value of the original 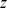-score. However, we differentiate between those edges with a positive 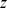-score (up-regulation) and those edges with a negative 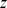-score (down-regulation). Figure 1(a–c) shows schematically how the protein array data is transformed into an unweighted bipartite graph. Alternatively, different thresholds can be used to filter up- and down-regulations.

The higher the 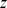-score threshold, the smaller the probability that the change in the protein level is merely a random fluctuation, and subsequently the fewer edges are present in the bipartite graph. As stated above, the goal is to understand mild regulation effects, which can only be analysed if the threshold is moderately low. In the following, we choose three thresholds: 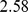 (corresponding to an unadjusted, two-sided 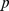-value of 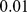), 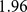 (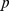-value of 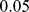), and 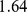 (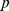-value of 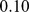). The unweighted bipartite graph that results from thresholding the weighted bipartite graph at 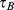 is henceforth called the *regulation graph* 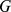 at 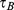.

### Co-regulation Graphs: One-mode Projection of Bipartite Graphs

In the setting described above, we are interested in the co-regulating behaviour of either the proteins or the protein-regulating conditions (miRNAs), i.e., we are interested in the indirect relation between nodes on the same side of the bipartite graph model. In essence, this requires creating a graph that contains only the nodes of 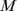 *or* 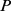. In the new graph, two nodes are connected if they take part in a significant number of co-regulation conditions. Such a graph is called a *one-mode projection* of the bipartite graph. Obviously, the bipartite graph can be projected onto either of the two sides.

As the bipartite graph model contains two different types of edges (up- and down-regulation effects), its one-mode projection displays the following relations that can be defined for both proteins and miRNAs, as illustrated by Figure 1D:

1. Co-up-regulation: 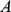 and 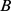 are both up-regulated by the same miRNA 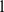, represented by the two red edges connecting 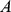 and 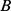 to 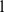;
2. Co-down-regulation: 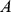 and 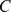 are both down-regulated by the same miRNA 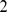, represented by the two green edges connecting 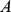 and 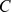 to 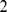;
3. Antagonistic regulation: 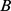 is down-regulated by miRNA 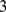 while 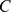 is up-regulated by it. This antagonistic co-regulation is denoted by a *directed* edge (represented by an arrow) from 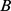 to 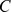.

Note that in principle, each pair of proteins or miRNAs could be connected through all four types of co-regulation patterns and thus be connected by all four possible edges (red, green, and a blue edge in both directions). In reality, we expect that two proteins or miRNAs are either 1) in only one relationship, or 2) at the same time co-up-regulated and co-down-regulated (connected by one green and one red edge), or 3) reversely co-regulated (blue edges in both directions).

In classic one-mode projections [24], an edge between two nodes on the same side of a bipartite graph is created if they share at least one neighbour on the other side, i.e., in our case one co-regulation event would be sufficient. In contrast, the newly proposed SICORE algorithm includes only statistically significant co-regulations in the one-mode projection. In the next section, we provide a sketch of the general method by which the statistical significance of a given network pattern is assessed, followed by the description of the necessary adaptations to regulation graphs.

### Assessing the Statistical Significance of Network Patterns

A firm assumption underlying network analysis is that a network’s structure follows its function [25]. It is therefore informative to look for substructures, so-called *network motifs* [26], [27], which occur more often than expected in a random network with the same degree sequence (for graph definitions see Text S1). This more than random idea corrects for those substructures which occur in a network with the same basic components but an otherwise random structure. There are different types of substructures of interest. One of them is, for example, the feed-forward loop, in which 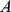 is influencing 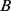 and 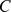, while 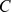 influences 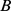. The method for the computation of the statistical significance of any kind of substructure in a network was introduced by Shen-Orr et al. [26], [27]. For instance, they showed that feed-forward loops are much more common in transcriptional regulation networks than expected. Their method can be described as follows:

1. Given a graph 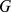 and a network pattern 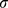, count the number of occurrences of this pattern in the whole graph 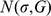;
2. Build a set of graphs 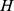 with the same degree sequence as 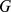 but otherwise randomly distributed edges.
3. Compute the number of occurrences of this pattern for all graphs 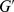 in 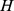 and compute the fraction 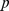 of graphs in which the number of occurrences of this pattern is at least as large as in the original graph 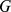.

The mathematical intuition behind this algorithm is the following: Let 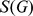 be the degree sequence of 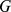 and let 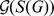 denote the set of all possible graphs with the same degree sequence as 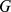, then the sample 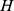 is a subset of 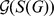. If 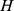 is large enough, then the fraction of graphs in 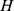 with at least as many occurrences of the pattern 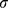 as contained in 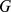 approximates the 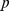-value of 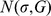 in the complete set 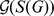. The complete set is generally too large to be enumerated, i.e., even for a small graph containing 20+20 nodes and 20 edges such that each node has degree 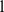, 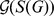 contains 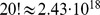 graphs. Since an exhaustive search is computationally not feasible, heuristic methods are preferred, namely only a sample 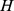 from this set is used to approximate the real 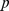-value. A low value implies that the observed occurrence of 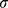 is less likely to be simply caused by the structure of the data but might rather hint at a functional correlation. In the following we present an extension of this network motif approach in which the patterns of interest are the different types of co-regulations.

### SICORE: Finding Significant Co-regulation Patterns in Regulation Graphs

Given 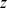-scores from a large-scale protein regulation experiment and a threshold 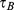 on the observations to be included into the graph model, the number of co-regulation conditions can be computed for each pair of proteins. Vice versa, the number of co-regulated proteins can be computed for each pair of regulating conditions. We want to understand whether the resulting numbers are actually significant or might 1) be just a random effect caused by noise, 2) occur simply due to some of the proteins showing extreme variation in their level, or 3) result from many miRNAs targeting a central protein by both direct interference and indirect effects propagated through the gene regulatory network. According to the more than random idea, all of these problems can be mitigated by assessing the probability that this number of co-regulating conditions is observed in graphs with the given degree sequence. Only those numbers which are unlikely to be the result of this random model will then be accepted as significant. The main idea behind overcoming the first problem is that filtering random missing edges or randomly added edges will not induce significant numbers of co-regulation conditions. The second problem, namely proteins with an erratically jumping abundance level, will mainly induce random edges in the network. The random model can cope with both types of problems since a node with a higher degree will also have higher numbers of co-regulating conditions in the model. The third problem is that miRNAs with many indirect effects induce proteins with high degree. Their co-regulations are corrected by the same noise-filtering effect.

**Figure 2:**
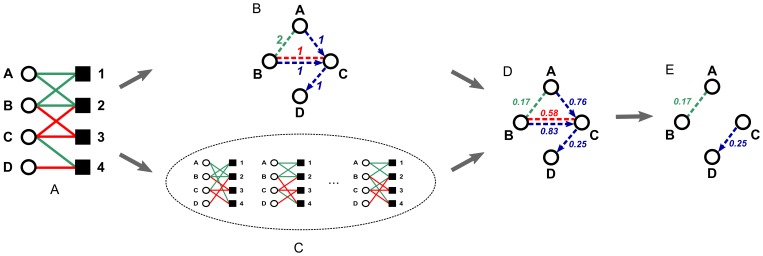
Steps performed by the SICORE algorithm. (A) Defining the initial bipartite graph, (B) counting the observed number of co-regulations, (C) simulating the set of random bipartite graphs which define the expected number of co-regulations, (D) building the protein co-regulation graph where the weight of the edges indicates the 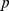-value assigned to the co-regulation of a given protein pair, (E) considering each co-regulation with a 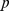-value smaller or equal than a threshold 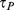 (e.g., 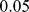) statistically significant.

Our method consists in adapting the scheme for the detection of network motifs in general graphs to the case of bipartite regulation graphs containing two types of edges: those corresponding to up-regulation and those corresponding to down-regulation (Figure 2A). The new algorithm is based on earlier work that aimed at finding significant co-occurrences in general bipartite graphs [28], [29]. Because there are two types of edges in bipartite regulation graphs, we need to maintain both the degree sequence of the up-regulations and the degree sequence of the down-regulations. The edge type specific degree sequence of each protein and each miRNA in the bipartite graph is then fixed while the edges of the same type are perturbed (Figure 2C). This is achieved by the so-called *edge swap* procedure [30]–[32]: two edges of the same type, e.g., (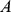,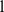) and (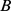,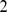), are picked uniformly at random. If (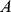,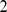) and (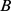,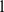) are not yet connected, edges (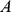,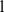) and (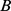,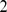) are removed and edges (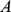,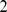) and (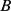,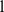) added. If at least one of the edges (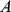,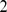) or (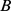,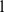) already exists, no such swap is performed. The edge swaps constitute a random walk (in mathematical terms a Markov chain) in the space of bipartite graphs with the same degree sequences for up- and down-regulations. It is thus assured that, if the number of attempted and conducted edge swaps is sufficiently large, the resulting graph is a uniform random sample from the set of all bipartite graphs with this fixed degree sequence [29], taking into account the two different types of edges. The first random walk starts at the observed bipartite graph, while subsequent walks start from the bipartite graph obtained in the previous step.

In summary, as sketched in Figure 2, the newly proposed SICORE algorithm performs the following steps to assess the statistical significance of the observed co-regulation patterns:

1. Given the observed data and a threshold 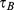, create the bipartite regulation graph 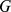 (Figure 2A).
2. For each pair of nodes on the side of interest in 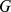, compute the number of all co-regulation conditions, sorted by type (Figure 2B).
3. Let 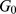 equal 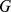 and let 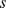 be the number of graphs in the sample 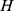.
4. For 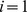 to 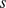 do:
  - Starting from 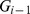, build graph 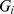 by performing edge swaps as described above (Figure 2C).
  - For all pairs of nodes on the side of interest in 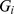, compute the number of all co-regulation conditions, sorted by type. If the number is at least as large as the observed value in 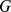, increase the empirical 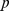-value of this pair and this type of co-regulation event by 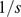 (Figure 2D).
5. Keep only those edges of the projection with an empirical 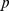-value below a threshold 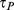 (Figure 2E). We address the procedure of choosing a proper threshold later on.

### Artificial Data for the Robustness Analysis

For the kind of question at hand, namely the co-regulation behaviour of proteins under various experimental conditions, there is, to our knowledge, no large data set where the correct result is known. We thus build artificial data sets for which the gold standard is defined by construction and test our method against it. This approach is often used in the clustering of networks, e.g., to prove the usefulness of the Girvan-Newman clustering algorithm [33] or to test the performance of clustering algorithms [34], [35].

**Figure 3:**
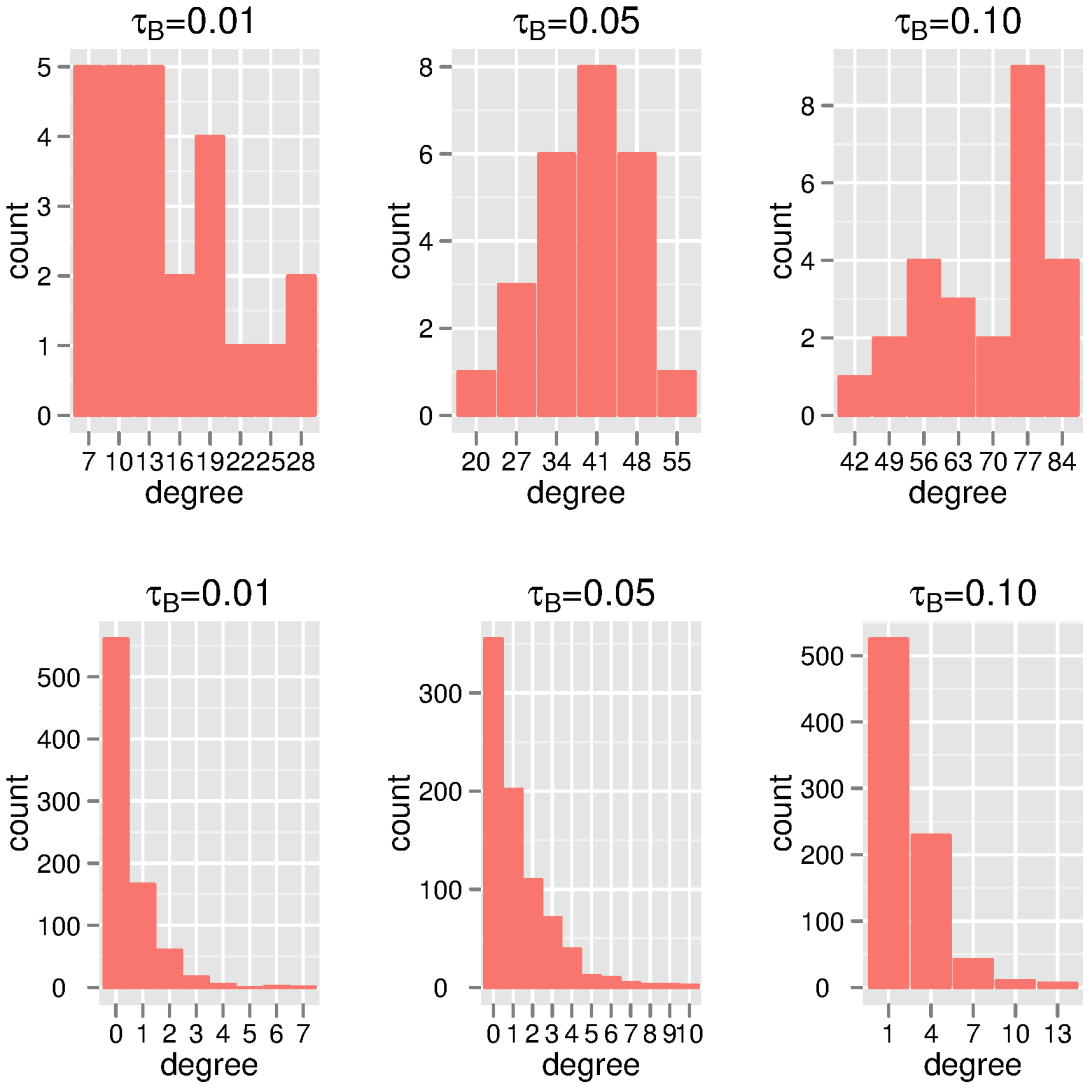
Degree distributions of the bipartite biological data. Shown are the degree distributions of proteins (top panel) and miRNAs (bottom panel) at thresholds 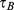 which correspond to 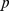-values of 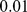, 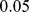 and 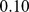.

**Figure 4:**
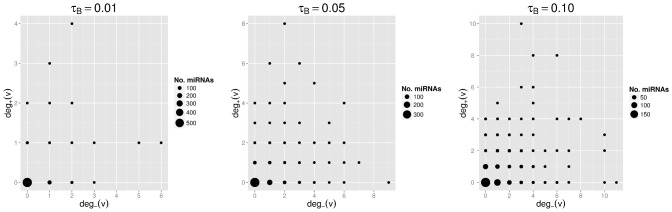
Up- and down-regulation effect of miRNAs. The weighted scatterplot shows the number of miRNAs for each combination of down- and up-regulation degrees.

In addition to constructing them in such a way that the optimal result is known, the artificial data sets should also have a structure which resembles the data the algorithm is applied to. For the biological data set at hand, there is a strong imbalance between the number of proteins (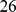) and the number of miRNAs (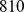). Moreover, their degree sequences (for a definition see Text S1) show a large variance (see Figure 3). Constructing artificial graphs that best fulfil these requirements at the same time is difficult and involves several modelling decisions. For illustration purposes, we formulate the simplifying assumptions behind the construction of the artificial graphs in terms of *artificial proteins* and *artificial miRNAs*: a) There are groups of artificial proteins that are either co-up- or co-down-regulated by a subset of artificial miRNAs. b) Such a group of up-regulated artificial proteins and a group of down-regulated artificial proteins are antagonistically regulated by some subset of artificial miRNAs. c) Each group of artificial miRNAs is responsible for up-regulating exactly one group of artificial proteins and down-regulating another group of artificial proteins. d) Additionally, the regulation effect of the artificial miRNAs is assumed to be half up- and half down-regulations. Note however, that real-world data might be biased towards one of the edge types. For instance, in the biological data set at hand, miRNAs have a preference for down-regulations (see Figure 4).

**Figure 5:**
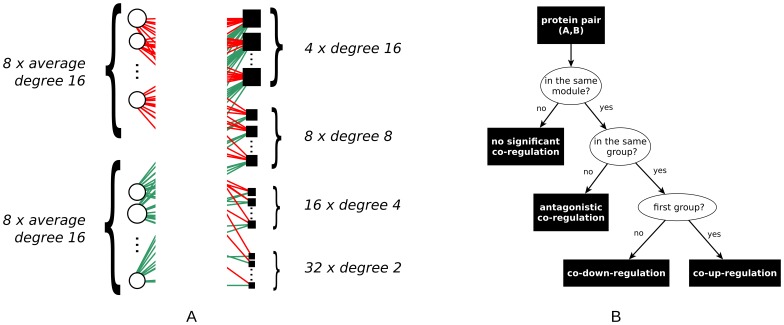
Structure of the artificial data. (A) Sketch of one module of an artificial graph. The degree of artificial proteins/miRNAs is proportional to size of circles/squares. (B) Decision tree illustrating the principle behind the construction of the gold standard.

To model these assumptions, we build artificial graphs consisting of five modules with 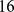 nodes on the left side and 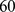 nodes on the right side, where the left side represents the artificial proteins and the right side the artificial miRNAs. In each module, there are 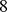 artificial proteins that are up-regulated and 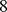 that are down-regulated by the artificial miRNAs in the same module. Each of these modules represents one group of artificial proteins that are up-regulated, and another group of artificial proteins that are down-regulated by the same group of artificial miRNAs. Figure 5A sketches the structure of a single module. The degree distributions of the artificial proteins and artificial miRNAs are chosen to be similar to the ones in our biological data set: The degree distribution of the artificial miRNAs is strongly skewed, i.e., four of the nodes have degree 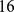, 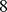 nodes have degree 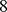, 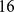 nodes have degree 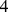, and 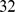 nodes have degree 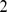, while artificial proteins have a Poisson degree distribution.

For these artificial graphs, the gold standard (i.e., the wanted result of a meaningful computation) when projecting to the artificial protein side is that within each of the modules all artificial proteins of the first group are significantly co-up-regulated, while all artificial proteins of the second group are significantly co-down-regulated. For any pair consisting of one artificial protein from the first and one from the second group, we require the algorithm to detect a significant antagonistic co-regulation directed from the second group to the first one. Defining a gold standard for the projection to the artificial miRNA side is not equally straightforward due to the presence of artificial miRNAs with a high degree, which will inherently be involved in non-significant co-regulations as well. However, since the robustness of the projection onto the artificial miRNA side is also highly relevant, we test the stability of the obtained artificial miRNA co-regulations with increasing noise. Thus, to show the stability of our method, the artificial data is further perturbed to model two types of noise typical for biological data:

1. false-negative observations, i.e., the miRNA does regulate the protein’s level but the change is too low due to random fluctuations, measuring errors, or simple handling errors. In this case, the regulation is not included in the regulation graph model and is thus a *missing edge*.
2. false-positive observations. By lowering the threshold of the original 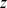-scores we *add edges* to the bipartite graph which are unlikely to represent significant regulations.

These two types of noise are modelled by altering the artificial data in the following way:

1. random elimination of a percentage 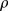 of edges (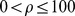) and
2. random addition of a percentage 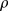 of edges (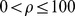).

The quality of the algorithm is measured by its ability to find the structure embedded in the original, artificial graph despite the presence of noise.

### Quality Measures for Evaluating the Predictions of SICORE

The gold standard of the artificial data set defines for each pair of proteins whether the algorithm should identify their co-regulation pattern as significant. As shown in Figure 5B, the gold standard partitions all pairs into co-regulated pairs of proteins 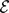 and not co-regulated pairs of proteins 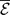. When projecting onto the protein-regulating conditions, the gold standard can be similarly defined.

Given a bipartite graph, our algorithm assigns a 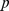-value to each protein pair which can then be sorted non-decreasingly by this value. For a fixed threshold 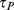 all pairs with a 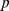-value lower than that threshold are predicted to be actually co-regulated. Compared with the gold standard, these pairs can either belong to 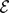 and thus be *true positives* (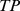), or belong to 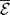 and be *false positives* (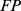). Analogously, pairs of proteins predicted to be not co-regulated might belong to 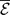 and thus be *true negatives* (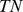) or belong to 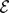 and be *false negatives* (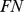).

Usually, prediction in bioinformatical problems is difficult because in most cases the set 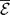 is substantially larger than 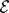. This is valid for our artificial data as well, because there are approximately 20 times less realized edges than possible ones. This implicit imbalance has to be taken into account when choosing the quality measures for evaluation. A trivial algorithm which always predicts a pair to be non-co-regulated would deceivingly result in a perfect *specificity* (correctly identified non-co-regulations). However, from a biological point of view, our interest focuses on the prediction of significantly co-regulated pairs, implying that the *sensitivity* (correctly predicted co-regulations) is more relevant. We thus need measures which combine specificity and sensitivity in a meaningful way.

Therefore, when assessing the performance of an algorithm, we first look at the 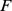-score which combines sensitivity (also called *recall*) and *precision* (the fraction of predicted edges that are true):
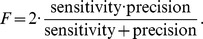



The 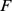-score is always in 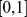, and the higher the score, the better the prediction. Having no false positive and no false negative predictions would result in an 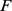-score of 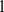.

The 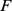-score depends on the arbitrarily chosen threshold for the observed 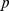-value, classically one of the following: 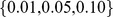. Another measure, the *positive predictive value* among the first 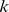 samples, the PPV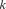, chooses a threshold such that for each of the co-regulation patterns exactly 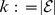 many pairs of proteins are predicted to be co-regulated [36]. 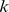 is thus determined by the number of edges in the gold standard. This measure is particularly helpful because of two important features it possesses. By definition, the PPV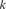 is equal to sensitivity. The higher the value, the more edges are among the first 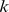 ranked samples. Second, it can be shown that if 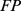 = 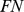 as in our case then PPV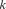 is proportional to specificity:
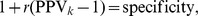
where 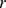 denotes the ratio between 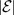 and 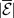. The values of PPV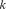 lie in the 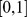 interval and a perfect predictor achieves 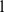.

## Results

We show the robustness of the proposed SICORE algorithm on artificial data and present its application to a challenging biological data set.

### Experiments on Artificial Data

**Figure 6:**
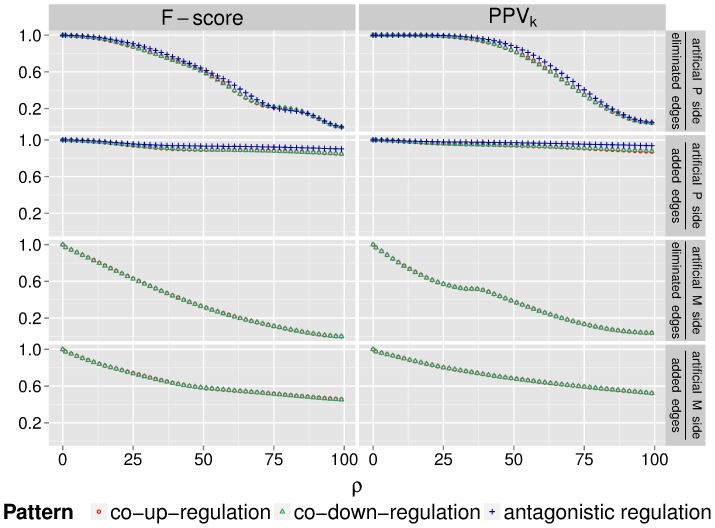
*F*-score and PPV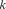 evaluating the performance of the SICORE algorithm on artificial data sets for increasing noise levels 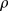. Results are shown for eliminated and added edges when projecting onto the artificial protein and the artificial miRNA side. Red data points represent the performance of predicting co-up-regulation, green data points refer to co-down-regulation, and blue ones to antagonistic co-regulation.

We construct 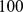 artificial graphs with predefined modular structure as described in the Materials and Methods section. In this section, whenever we refer to artificial proteins or artificial miRNAs, we use the terms *protein* and *miRNA*. Each artificial graph is projected twice: first to the protein side and then to the miRNA side. In order to assess the statistical significance of the edges in the projections, a sample of 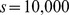 random graphs is used. Based on the projection onto the protein side (with an easily definable gold standard), our aim is to assess how well the SICORE algorithm recovers the built-in modular structure of the gold standard projection. Then, based on both projections, we test the robustness of the algorithm against elimination and addition of randomly chosen edges. To quantify the precision of the algorithm for different noise levels, we use the quality measures defined above. Figure 6 shows the performance of the algorithm when projecting onto the protein side (upper half) and when projecting onto the miRNA side (lower half). There are three patterns of interest for the protein case: when both proteins are up-regulated, both proteins are down-regulated, or one is up- while the other is down-regulated. For miRNAs, we only have two patterns: the antagonistic co-regulation pattern is omitted due to the lack of miRNA pairs in the original graphs that would antagonistically co-regulate proteins.

As both measures suggest, in the absence of noise, the algorithm recovers the protein modules perfectly. As the noise increases, the performance decays slowly. When projecting onto the protein side, gradual elimination of all edges in the bipartite graph (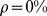 to 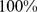) covers the whole range of possible prediction qualities. Accordingly, the 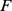-score drops from 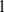 to 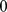 at 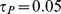 (the threshold used for determining the significance level of the edges that are included in the projection). The PPV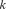 decreases from 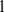 to about 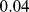 (for the co-up- and co-down-regulation patterns) and to 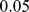 (for the antagonistic co-regulation), where the latter ones are the baseline values for this measure, i.e., the proportion of true positives among all samples. Up until the point where 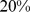 of all edges are eliminated, the PPV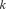 is almost perfect, while the 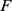-score is above 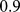 for all considered patterns. Thus, the algorithm compensates well for noise. The prediction accuracies when projecting onto the miRNA side show similar tendencies: for 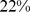 noise, the PPV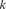 is about 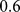, while the 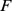-score is 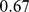.

The addition of edges exerts a milder effect on the prediction quality. Thus, for as many as 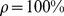 added edges, there are still many correct predictions. In this range, when projecting onto the protein side, the PPV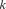 is above 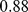 and the 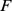-score exceeds 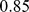 for all patterns. Projecting onto the miRNA side results in lower, but still convincing accuracies: the PPV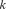 remains above 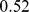, while the 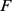-score always exceeds 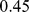. This is reassuring as it means that we can still find significant co-regulation patterns even when including mild effects into the original bipartite regulation graph.

Although the two chosen quality measures are conceptually different, the resulting performance plots are relatively similar in our case. The general trend is that, for low noise values, the PPV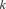 scores higher than the 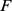-score. This is due to the different thresholds the two measures use. While PPV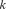 uses a threshold that is innate to the graph (the number of edge samples 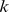), for the 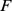-score we fix the threshold according to the rule of thumb 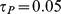. This emphasises that the proper choice of 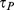 for the SICORE algorithm is crucial and needs further consideration. Overall, we conclude that the SICORE algorithm is robust against both investigated types of noise. Having thus validated it on artificial data, we proceed to the analysis of a real biological data set.

### Results on the Biological Data

As described above, the chosen biological data set contains the effect of a genome-wide library of miRNA mimics on the expression of 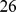 proteins in the EGFR-driven cell cycle pathway in a breast cancer cell line. Proteins are typically regulated by multiple miRNAs and miRNAs generally modulate, directly and/or indirectly, the expression of many proteins. Given these complex interactions between proteins and miRNAs, it is challenging to differentiate mild biological effects from technical fluctuations and to identify regulatory patterns. The SICORE algorithm is designed to detect on the one hand those pairs of proteins which are systematically co-targeted by a set of miRNAs, and on the other hand those pairs of miRNAs which systematically co-target a set of proteins. In this article, we use SICORE to search for miRNA pairs which simultaneously and significantly regulate the same proteins, i.e., we project the bipartite graph onto the miRNA side. For this, we use a sample of 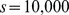 random graphs. Out of the obtained three projections, one for each co-regulation type, we focus on the biologically most relevant miRNA co-regulation pattern, namely co-down-regulation. A similar analysis can be performed on the other two projections consisting of co-up-regulations and antagonistic regulations.

The robustness analysis discussed above suggests that the choice of 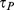 is one of the subtleties of the method that may influence the performance of SICORE considerably. Thus, we first discuss this final step of the algorithm (see Figure 2(e)). When interpreting the result of a statistical analysis, it is common practice to choose the threshold for the significance level by some rule of thumb. For instance, it is widely accepted to define the significance level as 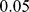 or 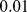. In contrast to this arbitrary choice of threshold, a trial and error approach is possible: one can set different thresholds and choose the best parameter by validating the results against prior knowledge or experiments, i.e., by using an *external reference approach*. Since external references might be difficult to obtain, we suggest the use of intrinsic properties like the network topology to automatically determine threshold candidates. The idea behind this *internal reference approach* is motivated by the core assumption in the analysis of biological networks namely that a network’s function is reflected by its structure [25], [37]. To find the significance threshold, one can thus use a general criterion that relies on network analytic reasoning and results in a network-specific threshold that is chosen based on the structure of the network rather than just on the underlying problem (similar ideas have been suggested in sociology [38], chemistry [39] and physics [40]). In an ideal setting, the two methods (the external and internal reference approaches) can be combined in order to maximize the efficiency of the predictions.

**Figure 7:**
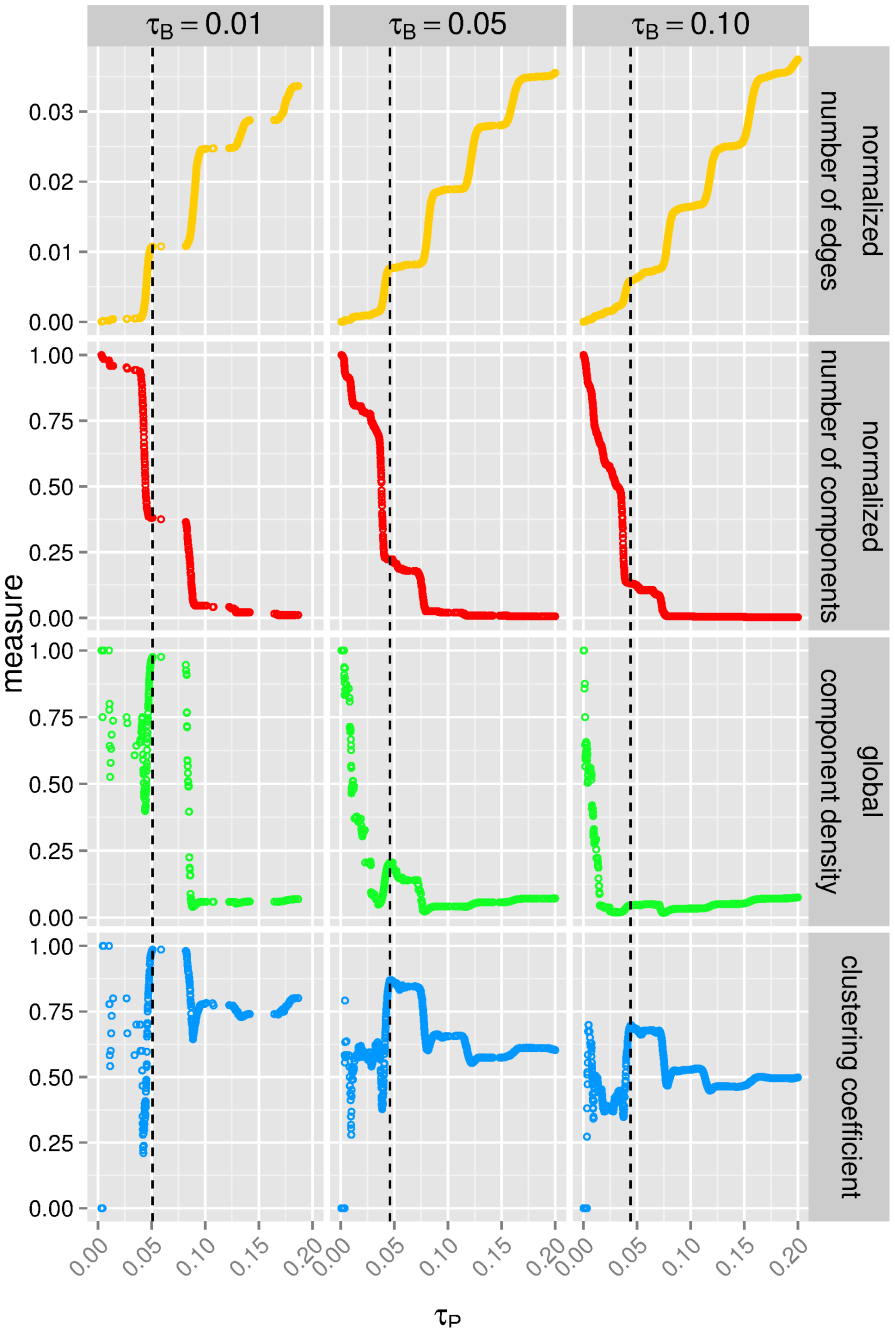
Deducing meaningful 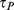 significance thresholds from structural measures. Shown are four graph topological measures against the 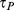 thresholds for the 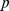-values of the projection. The projections onto the miRNA side are constructed from the bipartite graphs with thresholds 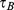 corresponding to a 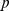-value of 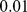, 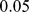 and 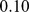.

To choose a proper threshold for miRNA co-regulations, we propose the internal reference approach and base the decision on intrinsic information deduced from the underlying graph. Thus, we search for an appropriate threshold by inspecting the topology of the sub-graphs built with different possible thresholds. Topological features of interest are: 1) the number of edges normalized by the maximum number of edges, 2) the number of components (i.e., sub-graphs in which any two nodes are connected to each other by paths), 3) the component density of the sub-graphs normalized by the maximum number of components, where the density of a component is defined as the total number of its edges divided by the number of possible edges, 4) and the clustering coefficient that quantifies the probability that any two of a node’s neighbours are connected themselves [41]. The clustering coefficient of a graph is the average clustering coefficient of its nodes. In our case, it measures the probability that two miRNAs, which each co-regulate proteins with a given third miRNA, also co-regulate the same protein(s). Monitoring these features at varying thresholds, we observe nontrivial changes in the structure of the sub-graphs indicating the more informative threshold candidates (see Figure 7). The thresholds are considered optimal when there is a strong increase or local maximum in the clustering coefficient and in the global component density, while the number of components is still considerable. With respect to miRNA co-regulation, these criteria assure increased transitivity and best reveal the local connection patterns of the individual miRNAs. Accordingly, for our data we choose the 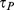 thresholds shown in Table 1. Interestingly, for this data set, the thresholds for the statistical significance of the co-regulations do not differ considerably for altered significance levels 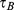 of the edges in the bipartite graph.

**Table 1:** Properties of the co-down-regulation projections obtained from the bipartite graphs with different 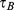 thresholds.

*τ_B_*	0.10	0.05	0.01
*τ_P_*	0.0440	0.0459	0.0509
number of miRNAs	437	322	151
number of groups	33	42	31

Shown are the significance thresholds 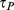 for the edges in the corresponding co-down-regulation graphs alongside the number of miRNAs and groups of size 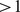 obtained at those thresholds.

**Figure 8:**
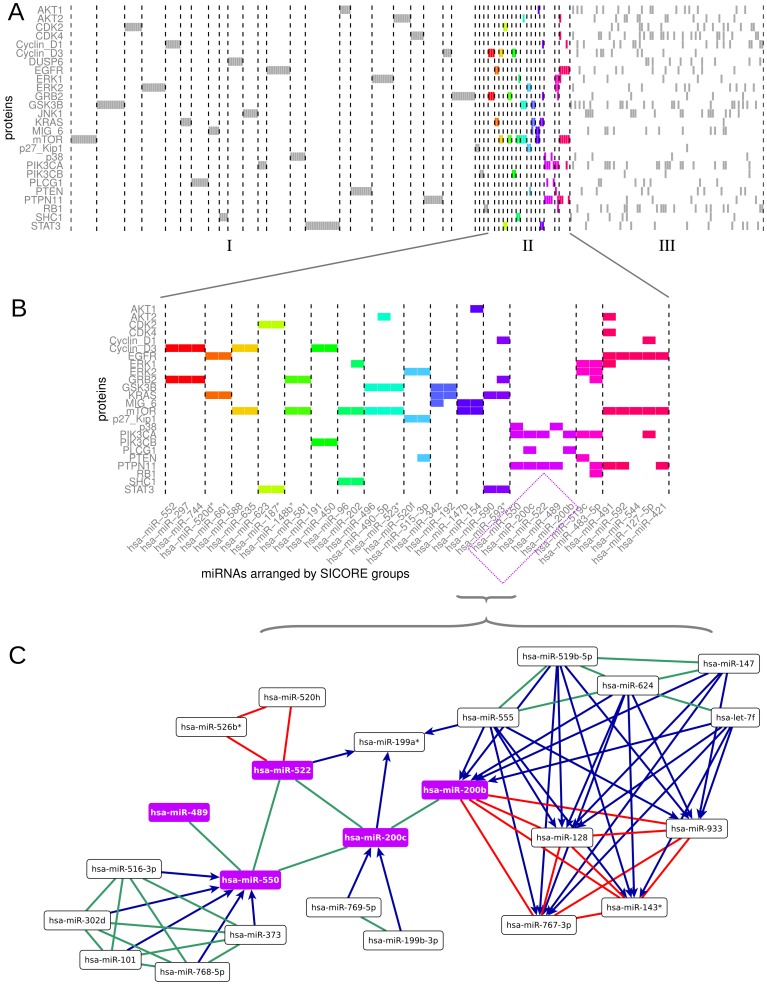
miRNA groups obtained by the SICORE algorithm from the bipartite graph with 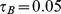 and 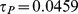. Each square represents a down-regulation in the bipartite graph. (A) Shown are the groups with one exclusive protein target (section I), with 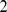 to 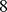 targets (section II, coloured regulations), and with multiple targets (section III). The names of all shown miRNAs and their protein targets are listed in the order of their appearance in in the figure in Table S4. (B) Magnification of section II containing nontrivial co-regulations. In accordance with (A), colours indicate the different SICORE groups. (C) The SICORE group containing hsa-miR-489, hsa-miR-522, hsa-miR-200c, hsa-miR-550, and hsa-miR-200b together with the co-regulating miRNAs. Co-down-regulation is shown in green, co-up-regulation in red, while antagonistic regulation is coloured blue (the miRNA at the source of the arrow significantly down-regulates and the miRNA at the head of the arrow significantly up-regulates).

Analysing the effect of the bipartite graph threshold on the resulting co-regulation graphs (Table S3), we observe that as 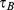 gets stricter, these projections contain a decreasing number of miRNAs that are grouped in several components of size 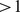 (see Table 1). We call these groups of miRNAs the SICORE groups. (Figure S1 shows these SICORE groups for all three thresholds 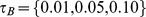.) First, to reinforce the assumption that SICORE detects miRNA groups which have similar regulation patterns, we return to the bipartite graph model and analyse it with respect to the newly acquired grouping of the miRNAs. As shown in Figure 8, based on the number of proteins that are co-targeted by the miRNAs contained in the SICORE groups, we can differentiate between three types of groups:

1. Groups of miRNAs that target one single protein (section I in Figure 8A). Although they do not provide new biological insights, these groups are reassuring findings since they obviously satisfy the criterion of non-random co-regulation;
2. Groups of miRNAs that have 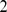 to 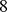 protein targets (section II in Figure 8A and magnified in Figure 8B). These groups represent nontrivial co-regulations and should be central to further experimental investigations aimed at finding candidates for new tumour suppressors; and
3. One larger group that contains several miRNAs with multiple targets (section III in Figure 8A). Here the interconnectedness in the bipartite graph is highly complex and requires further research. For instance, the group could be split up by lowering the projection threshold 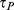 or using a subsequent clustering algorithm which detects subgroups based on the 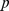-values assigned to each miRNA pair.

Figure 8C shows an exemplary excerpt of the co-regulation graph (the five miRNAs belonging to one of the SICORE groups and the co-regulating miRNAs) with typical patterns for the entire graph. Accordingly, co-up- and co-down-regulations define tightly connected clusters. Antagonistic co-regulations occur *between* these clusters, systematically connecting co-down-regulated clusters with co-up-regulated clusters, i.e., consistently with their direction.

We expect that the membership of the miRNAs in the SICORE groups is biologically meaningful. To test this, we analyse the groups in relation to the assignment of miRNAs into families according to their *seed sequence* – a non-disrupted subsequence between the 2nd and 7th bases of the mature miRNA, which is believed to be decisive for RNA binding. Specifically, we compare the seed sequences of miRNAs belonging to the same SICORE group. To quantify the similarity of two miRNAs, we use the edit distance of their seed sequences, i.e., the number of alterations required to change one sequence into the other. The similarity of the miRNAs which SICORE places in the same group is then defined as the average pairwise edit distance between the miRNAs. To test whether the sequence similarity within a given group is statistically significant, we conduct simulation with bootstrapping. In some of the cases, the edit distances suggest a significant similarity between the sequences in the SICORE groups (see Figure S2). As shown in Table 2, a hypergeometric test reveals that for 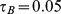 there are 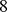 over-represented families in the SICORE groups. Four of these families are reported to be oncogene or tumour suppressors in breast cancer, while two of them, miR-99 and miR-506, have a role in prostate/head-and-neck cancer and melanoma, respectively. Thus, by using the SICORE algorithm we can extract miRNAs and families which have already established roles in the pathogenesis of breast cancer. This implies the ability of our algorithm to identify the potentially most pathologically-relevant miRNAs.

**Table 2:** miRNA groups identified by the SICORE algorithm in which the precursor families are significantly over-represented.

group	enriched miRNA	# miRNAs in	# miRNAs of this family	# miRNAs of	miRNAs of the family	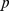 .hyper
index	precursor family	the group	having 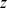 -scores	this family	that are in the group as well	
			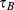 over the threshold	in the group		
1	mir-99	12	4	3	hsa-miR-100, hsa-miR-99a,	0.001
					hsa-miR-99b	
5	let-7	90	7	5	hsa-let-7f, hsa-let-7f-1*,	0.029
					hsa-let-7f-2*, hsa-let-7g*,	
					hsa-let-7i*	
17	mir-146	11	3	2	hsa-miR-146a, hsa-miR-146b	0.005
19	mir-221	16	4	2	hsa-miR-221, hsa-miR-222	0.011
19	mir-29	16	2	2	hsa-miR-29a, hsa-miR-29c	0.001
33	mir-506	9	7	2	hsa-miR-509-3-5p, hsa-miR-510	0.018
42	mir-8	5	5	2	hsa-miR-200b, hsa-miR-200c	0.001
45	mir-515	2	61	2	hsa-miR-515-3p, hsa-miR-520f	0.021

For analysis, we consider the seed sequences of the groups obtained at the regulation stringency threshold 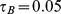. The statistical significance of over-representation was assessed by a hypergeometric test. The complete list of over-represented families for all used stringency thresholds 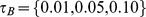 can be found in Table S5.

## Discussion

Since the early days of genetics and molecular biology, it has been noted that proteins can be regulated by more than one regulator and one regulator may in turn affect several proteins. In many situations, a regulator or a given experimental condition exerts only a mild effect on an observed protein, which might be difficult to differentiate from a random fluctuation. To address this complication, we propose a network analytic method called SICORE which is rooted in the observation that if proteins are collaborating with each other to coerce a common biological function, then this should be reflected in the way they are co-regulated. Based on this assumption, we search for pairs of proteins or protein-regulating agents, which are significantly co-regulated under many different experimental conditions. In a biological system with many layers of regulatory networks, co-regulations may contribute to the robustness of the system, since the regulation can be resistant to partial losses of functional members due to gene deletion, mutation, or stochastic expression regulations. Understanding co-regulation is vital in establishing an effective and stable modulation of the molecular target and thus it is important for cellular engineering and drug research.

Given a complex interconnected system of proteins and regulators, SICORE finds statistically significant co-regulations. In this article, we have shown on artificial data sets that systematic co-regulations are detected even in the presence of random noise in the form of eliminated or added regulations. To test SICORE on a real biological data set, we applied it to the EGFR-driven cell cycle system regulated by miRNAs. The biological function of miRNAs is only partially understood and the regulation of signalling networks by miRNAs is highly complex. In particular, little is known about physiological relevance of co-regulated protein pairs by miRNAs. It is believed that such co-regulations within a network confer signalling robustness (e.g., dampening and buffering effect) and can mediate the crosstalk of different signalling pathways [42]. Two different kinds of co-regulation patterns can occur: several miRNAs co-regulate a single protein and a single miRNA might co-regulate several proteins. For example, one of the first discovered miRNAs lin-4 and let-7 were identified to cooperatively target the gene lin-28 [43]. Similarly, miRNAs let-7b, miR-375 and miR-124 were validated to cooperatively control Mtpn in mammals [44]. In the study of Wu et al., the CDKI p21Cip1/Waf1 was shown to be directly targeted by 28 miRNAs in a high-throughput luciferase reporter screen [45]. Similarly to these examples, we identified novel co-regulations of proteins which belong to the same functional modules at genome-wide miRNA level [20]. Interestingly, the expression of several key proteins controlling the G1/S transition was regulated in a tightly coordinated manner by the studied miRNAs and we could identify co-regulated protein pairs with a possible physiological relevance. For example, we found that miR-520d*/miR-661 co-down-regulate EGFR and KRAS. This co-down-regulation could be a two-tier regulation at the receptor and pathway level to ensure robust control of two key oncogenes in cancer. Taken together, all these findings indicate that miRNAs should be studied further on a system-wide level to understand their regulation in the context of biological networks, thereby going beyond the level of individual interactions between miRNAs and their corresponding targets. This article represents a further step in this direction.

Focusing on miRNA co-regulation, we showed with sequence analysis and miRNA family enrichment analysis that the theory, according to which miRNA targeting is sequence-dependent, indeed partially explains the observed co-regulations obtained by SICORE. However, the results of the SICORE algorithm show that even miRNAs with distinct seed regions can induce strong co-regulations, which may be caused by the co-targeting of upstream transcription factors or separate targeting of canalized pathways. This indicates the complexity of the miRNA regulatory machinery, since miRNAs from different families may target different genes while yielding the same output. To tackle this complexity, further experiments are needed, such as profiling gene expression by over-expressing miRNAs of the same SICORE groups. Our results do not only yield proteomic evidences that sequence similarity of miRNAs determine their targets, but also provide hypotheses of other types of co-targeting that can be tested experimentally. Thus, potential therapeutic applications have to consider miRNA sets with similar co-regulation patterns. Based on our observations, we therefore argue that systematic approaches examining regulations between two biological components (miRNA and EGFR pathway proteins in our case) can be essential to the detection of co-regulation patterns and in the design of multiplex targeting strategies.

High-throughput studies aiming at exploiting regulatory networks between two types of biological entities have been made feasible thanks to technological development and community efforts. Recently, as the ENCODE project reached its milestone, several data sets and accompanying papers were published (for a review see [46]), providing data in various settings that can be modelled by bipartite graphs, e.g., transcription factors binding to DNA promoter regions [47], gene-coding RNAs and co-transcriptional long non-coding RNAs [48], single-nucleotide polymorphisms (SNPs) and diseases [49]. Despite their distinct natures, all these data sets can be represented as bipartite graphs and therefore be analysed by the SICORE algorithm to identify significant co-regulation patterns. Previous approaches of finding such patterns include various clustering methods, most prominently hierarchical clustering or *k-means* clustering. SICORE differs from these methods in four important aspects:

1. It applies thresholding when building the bipartite graph model. We reckon that this step can be both advantageous and risky: by using a hard threshold, on the one hand we filter out noise, but on the other we may disregard potentially useful information by eliminating edges. However, benchmarking with artificial data sets suggests that SICORE is highly robust against randomly added edges (noise included due to a loose threshold) or eliminated edges (relevant regulation lost because of a strict threshold). This gives us flexibility when choosing the threshold 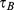, suggesting that small deviations of the threshold may not have a considerable impact on the algorithm.
2. The co-regulation graph with the threshold 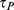 is selected by tracing changes in the graph characteristics with respect to the threshold choice. Instead of relying on rules of thumb, this allows for a threshold-selection which retains a maximum of information obtainable from primary data.
3. Classical hierarchical clustering returns a tree in which each biological entity (e.g., miRNA) is connected to another entity via an internal node. The k-means clustering results in groups of nodes without internal edges. In comparison, SICORE provides an intuitive way of understanding active or passive co-regulation relations within the groups.
4. For each identified co-regulation, it reports an empirical 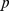-value which quantifies the likelihood of observing the given co-regulation patterns in random graphs. This is not the case for either hierarchical clustering or k-means. Therefore, SICORE makes it possible to compare the statistical significance of the co-regulations within one network as well as between different networks. Comparing significant co-regulation patterns (network motifs), instead of comparing top hits, may help in revealing the mechanisms underlying observations of interest, as pathway and network analysis has demonstrated in microarray analysis [50].

Besides these classical clustering methods, weighted correlation network analysis (WGCNA) has been proposed [51] and successfully applied in gene expression microarray analysis [52]. WGCNA assumes a scale-free topology of the underlying network, while SICORE does not make any assumption regarding the structure of the data. Thus, we believe that it offers an unbiased analysis as compared to WGCNA. A thorough comparison between SICORE and other existing approaches represents the main direction for future research. An exemplary comparison of our method with the Pearson correlation of the expression values, i.e., one of the standard methods for evaluating gene co-expression [53], showed that SICORE outperforms this on artificial data sets. Accordingly, when identifying co-regulated proteins from data sets containing 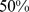 noise in form of added edges, the Pearson correlation achieves a 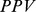 of 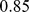, while our method has a performance of 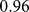.

The biological analysis presented in this article as well as the one analysing protein co-regulation patterns in the EGFR-driven cell cycle system [20] have been used to illustrate *one* context in which the SICORE algorithm can be used. An entirely different application of the algorithm on a data set of film ratings can be found in [54]. We encourage the reader to use it in other settings. The method can be applied as long as the biological system of interest can be modelled as a bipartite graph *and* the research question can be meaningfully approached in terms of co-occurrences of nodes of the same type. The statistically significant co-occurrences identified by the algorithm are expected to unravel functional groups which could be profitably analysed from this perspective.

## Supporting Information

Figure S1
**miRNAs arranged by SICORE groups and their respective protein targets.** Shown are all groups of size 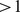 obtained from the bipartite graphs with 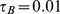, 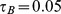, and 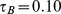, respectively. Each coloured rectangle represents a down-regulation in the bipartite graph. Colours mark SICORE groups. The top row indicates the bipartite stringency level at which a given miRNA was *first* considered for analysis: black denotes the bipartite graph with 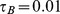, grey denotes 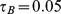, and white denotes 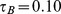. With decreasing stringency levels, miRNAs added to the analysis form new groups in some of the cases. In general however, they show no preferences when enriching existing groups.(EPS)

Figure S2
**Statistical significance of the similarity of miRNA seed sequences within the SICORE groups.** For each significance level 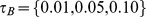, solid and dashed lines show the medians and the 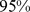 confidence intervals of the average edit distances per group when permuting members of each group for 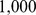 times. Dots indicate the actual distance of the miRNAs in the individual SICORE groups. Groups marked with red dots have significantly lower edit distances than expected by chance (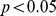). There are 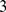 such groups for 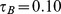, 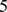 for 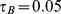, and 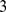 for 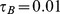.(EPS)

Table S1 **Investigated miRNAs.**
(PDF)

Table S2 **Investigated proteins.**
(PDF)

Table S3 **Groups of miRNAs defined by the SICORE algorithm and their corresponding sequences.**
(PDF)

Table S4 **List of miRNAs and their protein targets shown in** **Figure 8A** **.**
(PDF)

Table S5
**SICORE groups in which the precursor families are significantly over-represented for regulation stringency thresholds 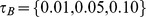.**
(PDF)

Text S1 **Graph definitions.**
(PDF)
