# Supplementary material for: A Network-Based Method to Assess the Statistical Significance of Mild Co-Regulation Effects
Source: PLoS One. 2013 Sep 9;8(9):e73413. doi: 10.1371/journal.pone.0073413 (PMC3767771; doi:10.1371/journal.pone.0073413)
Supplement: Table S1 — Investigated miRNAs. (PDF) [file pone.0073413.s003.pdf]

Protein symbol	UniProtKB	Protein name
AKT1	P31749	v-akt murine thymoma viral oncogene homolog 1
AKT2	P31751	v-akt murine thymoma viral oncogene homolog 2
Cyclin D1	P24385	cyclin D1
Cyclin D3	P30281	cyclin D3
CDK2	P24941	cyclin-dependent kinase 2
CDK4	P11802	cyclin-dependent kinase 4
p27/Kip1	P46527	cyclin-dependent kinase inhibitor 1B
DUSP6	Q16828	dual specificity phosphatase 6
EGFR	P00533	epidermal growth factor receptor
MIG-6	Q9UJM3	mitogen-inducible gene-6
mTOR	P42345	mechanistic target of rapamycin
GRB2	P62993	growth factor receptor-bound protein 2
GSK3B	P49841	glycogen synthase kinase 3 beta
KRAS	P01116	v-Ki-ras2 Kirsten rat sarcoma viral oncogene homolog
ERK2	P28482	extracellular signal-regulated kinase-2
p38	Q16539	MAP kinase p38 alpha
ERK1	P27361	extracellular signal-regulated kinase-1
JNK1	P45983	Jun N-terminal Kinase
PIK3CA	P42336	phosphoinositide-3-kinase, catalytic, alpha polypeptide
PIK3CB	P42338	phosphoinositide-3-kinase, catalytic, beta polypeptide
PLCG1	P19174	phospholipase C, gamma 1
PTEN	P60484	phosphatase and tensin homolog
PTPN11	Q06124	protein tyrosine phosphatase, non-receptor type 11
RB1	P06400	retinoblastoma 1
SHC1	P29353	SHC (Src homology 2 domain containing) transforming protein 1
STAT3	P40763	signal transducer and activator of transcription 3 (acute-phase response factor)
