# Supplementary material for: A Network-Based Method to Assess the Statistical Significance of Mild Co-Regulation Effects
Source: PLoS One. 2013 Sep 9;8(9):e73413. doi: 10.1371/journal.pone.0073413 (PMC3767771; doi:10.1371/journal.pone.0073413)
Supplement: Table S2 — Investigated proteins. (PDF) [file pone.0073413.s004.pdf]

Dharmacon miRIDIAN® microRNA Library- Human Mimic

CS-001005 Lot 08123

Plate	Well	Catalog Number	Mature Sanger ID	PreCursor Accession	Mature Sequence
Plate 1	A02	C-300474-03	hsa-let-7a	MI0000061	UGAGGUAGUAGGUUGUAUAGUU
Plate 1	A03	C-300477-03	hsa-let-7c	MI0000064	UGAGGUAGUAGGUUGUAUGGUU
Plate 1	A04	C-300482-03	hsa-miR-15a	MI0000069	UAGCAGCACAUAAUGGUUUGUG
Plate 1	A05	C-300483-03	hsa-miR-16	MI0000070	UAGCAGCACGUAAAUAUUGGCG
Plate 1	A06	C-300488-03	hsa-miR-19a	MI0000073	UGUGCAAAUCUAUGCAAAACUGA
Plate 1	A07	C-300489-03	hsa-miR-19b	MI0000074	UGUGCAAAUCCAUGCAAAACUGA
Plate 1	A08	C-300491-03	hsa-miR-20a	MI0000076	UAAAGUGCUUAUAGUGCAGGUAG
Plate 1	A09	C-300492-03	hsa-miR-21	MI0000077	UAGCUUAUCAGACUGAUGUUGA
Plate 1	A10	C-300493-03	hsa-miR-22	MI0000078	AAGCUGCCAGUUGAAGAACUGU
Plate 1	A11	C-300494-03	hsa-miR-23a	MI0000079	AUCACAUUGCCAGGGAUUUCC
Plate 1	B02	C-300497-03	hsa-miR-24	MI0000081	UGGCUCAGUUCAGCAGGAACAG
Plate 1	B03	C-300502-03	hsa-miR-27a	MI0000085	UUCACAGUGGCUAAGUUCCGC
Plate 1	B04	C-300505-03	hsa-miR-30a-5p	MI0000088	UGUAAACAUCCUCGACUGGAAG
Plate 1	B05	C-300506-03	hsa-miR-30a-3p	MI0000088	CUUUCAGUCGGAUGUUUGCAGC
Plate 1	B06	C-300516-03	hsa-miR-99a	MI0000101	AACCCGUAGAUCCGAUCUUGUG
Plate 1	B07	C-300517-03	hsa-miR-100	MI0000102	AACCCGUAGAUCCGAACUUGUG
Plate 1	B08	C-300522-03	hsa-miR-103	MI0000108	AGCAGCAUUGUACAGGGCUAUGA
Plate 1	B09	C-300523-03	hsa-miR-103	MI0000109	AGCAGCAUUGUACAGGGCUAUGA
Plate 1	B10	C-300527-03	hsa-miR-107	MI0000114	AGCAGCAUUGUACAGGGCUAUCA
Plate 1	B11	C-300528-03	hsa-miR-192	MI0000234	CUGACCUAUGAAUUGACAGCC
Plate 1	C02	C-300533-03	hsa-miR-199a	MI0000242	CCCAGUGUUCAGACUACCUGUUC
Plate 1	C03	C-300537-03	hsa-miR-208	MI0000251	AUAAGACGAGCAAAAAGCUUGU
Plate 1	C04	C-300539-03	hsa-miR-129	MI0000473	CUUUUUGCGGUCUGGGCUUGC
Plate 1	C05	C-300541-03	hsa-miR-30c	MI0000254	UGUAAACAUCCUACACUCUCAGC
Plate 1	C06	C-300543-03	hsa-miR-30d	MI0000255	UGUAAACAUCCCCGACUGGAAG
Plate 1	C07	C-300549-03	hsa-miR-10a	MI0000266	UACCCUGUAGAUCCGAAUUUGUG
Plate 1	C08	C-300556-03	hsa-miR-181c	MI0000271	AACAUUCAACCUGUCGGUGAGU
Plate 1	C09	C-300562-03	hsa-miR-203	MI0000283	GUGAAAUGUUUAGGACCACUAG
Plate 1	C10	C-300565-03	hsa-miR-210	MI0000286	CUGUGCGUGUGACAGCGGCUGA
Plate 1	C11	C-300566-03	hsa-miR-211	MI0000287	UUCCCUUUGUCAUCCUUCGCCU
Plate 1	D02	C-300567-03	hsa-miR-212	MI0000288	UAACAGUCUCCAGUCACGGCC
Plate 1	D03	C-300574-03	hsa-miR-218	MI0000295	UUGUGCUUGAUCUAACCAUGU
Plate 1	D04	C-300590-03	hsa-miR-30b	MI0000441	UGUAAACAUCCUACACUCAGCU
Plate 1	D05	C-300595-03	hsa-miR-125b	MI0000446	UCCCUGAGACCCUAACUUGUGA
Plate 1	D06	C-300596-03	hsa-miR-125b	MI0000470	UCCCUGAGACCCUAACUUGUGA
Plate 1	D07	C-300598-03	hsa-miR-130a	MI0000448	CAGUGCAAUGUUAAAAGGGCAU
Plate 1	D08	C-300599-03	hsa-miR-132	MI0000449	UAACAGUCUACAGCCAUGGUCG
Plate 1	D09	C-300602-03	hsa-miR-135a	MI0000452	UAUGGCUUUUUAUUCCUAUGUGA
Plate 1	D10	C-300608-03	hsa-miR-141	MI0000457	UAACACUGUCUGGUAAAGAUGG
Plate 1	D11	C-300610-03	hsa-miR-142-3p	MI0000458	UGUAGUGUUUCCUACUUUAUGGA
Plate 1	E02	C-300619-03	hsa-miR-9	MI0000467	UCUUUGGUUAUCUAGCUGUAUGA
Plate 1	E03	C-300620-03	hsa-miR-9	MI0000468	UCUUUGGUUAUCUAGCUGUAUGA
Plate 1	E04	C-300627-03	hsa-miR-127	MI0000472	UCGGAUCCGUCUGAGCUUGGCU
Plate 1	E05	C-300629-03	hsa-miR-136	MI0000475	ACUCCAUUUGUUUUGAUGAUGGA

Plate 1	E06	C-300630-03	hsa-miR-146a	MI0000477	UGAGAACUGAAUUCCAUGGGUU
Plate 1	E07	C-300632-03	hsa-miR-150	MI0000479	UCUCCCAACCCUUGUACCAGUG
Plate 1	E08	C-300633-03	hsa-miR-154	MI0000480	UAGGUUAUCCGUGUUGCCUUCG
Plate 1	E09	C-300634-03	hsa-miR-154*	MI0000480	AAUCAUACACGGUUGACCUAUU
Plate 1	E10	C-300635-03	hsa-miR-184	MI0000481	UGGACGGAGAACUGAUAAGGGU
Plate 1	E11	C-300639-03	hsa-miR-190	MI0000486	UGAUAUGUUUGAUAUAUUAGGU
Plate 1	F02	C-300642-03	hsa-miR-194	MI0000732	UGUAACAGCAACUCCAUGUGGA
Plate 1	F03	C-300643-03	hsa-miR-195	MI0000489	UAGCAGCACAGAAAUAUUGGC
Plate 1	F04	C-300644-03	hsa-miR-206	MI0000490	UGGAAUGUAAGGAAGUGUGUGG
Plate 1	F05	C-300654-03	hsa-miR-34b	MI0000742	UAGGCAGUGUCAUUAGCUGAUUG
Plate 1	F06	C-300655-03	hsa-miR-34c	MI0000743	AGGCAGUGUAGUUAGCUGAUUGC
Plate 1	F07	C-300656-03	hsa-miR-299-3p	MI0000744	UAUGUGGGAUGGUAAACCGCUU
Plate 1	F08	C-300657-03	hsa-miR-301	MI0000745	CAGUGCAAUAGUAUUGUCAAAGC
Plate 1	F09	C-300658-03	hsa-miR-99b	MI0000746	CACCCGUAGAACCGACCUUGCG
Plate 1	F10	C-300659-03	hsa-miR-296	MI0000747	AGGGCCCCCCCUCAAUCCUGU
Plate 1	F11	C-300666-03	hsa-miR-365	MI0000767	UAAUGCCCCUAAAAAUCCUUAU
Plate 1	G02	C-300667-03	hsa-miR-365	MI0000769	UAAUGCCCCUAAAAAUCCUUAU
Plate 1	G03	C-300675-03	hsa-miR-369-3p	MI0000777	AAUAAUACAUGGUUGAUCUUU
Plate 1	G04	C-300678-03	hsa-miR-372	MI0000780	AAAGUGCUGCGACAUUUGAGCGU
Plate 1	G05	C-300679-03	hsa-miR-373*	MI0000781	ACUCAAAAUGGGGGCGCUUUCC
Plate 1	G06	C-300680-03	hsa-miR-373	MI0000781	GAAGUGCUUCGAUUUUGGGGUGU
Plate 1	G07	C-300683-03	hsa-miR-376a	MI0000784	AUCAUAGAGGAAAAUCCACGU
Plate 1	G08	C-300857-01	hsa-miR-376a	MI0003529	AUCAUAGAGGAAAAUCCACGU
Plate 1	G09	C-300684-03	hsa-miR-377	MI0000785	AUCACACAAAGGCAACUUUUGU
Plate 1	G10	C-300685-03	hsa-miR-378	MI0000786	CUCCUGACUCCAGGUCCUGUGU
Plate 1	G11	C-300688-03	hsa-miR-380-5p	MI0000788	UGGUUGACCAUAGAACAUGCGC
Plate 1	H02	C-300689-03	hsa-miR-380-3p	MI0000788	UAUGUAAUAUGGUCCACAUCUU
Plate 1	H03	C-300690-03	hsa-miR-381	MI0000789	UAUACAAGGGCAAGCUCUCUGU
Plate 1	H04	C-300691-03	hsa-miR-382	MI0000790	GAAGUUGUUCGUGGUGGAUUCG
Plate 1	H05	C-300692-03	hsa-miR-383	MI0000791	AGAUCAGAAGGUGAUUGUGGCU
Plate 1	H06	C-300694-03	hsa-miR-330	MI0000803	GCAAAGCACACGGCCUGCAGAGA
Plate 1	H07	C-300695-03	hsa-miR-328	MI0000804	CUGGCCCUCUCUGCCCUUCCGU
Plate 1	H08	C-300699-03	hsa-miR-326	MI0000808	CCUCUGGGCCCUUCCUCCAG
Plate 1	H09	C-300702-03	hsa-miR-148b	MI0000811	UCAGUGCAUCACAGAACUUUGU
Plate 1	H10	C-300704-03	hsa-miR-324-5p	MI0000813	CGCAUCCCCUAGGGCAUUGGUGU
Plate 1	H11	C-300712-03	hsa-miR-346	MI0000826	UGUCUGCCCGCAUGCCUGCCUCU
Plate 2	A02	C-300723-03	hsa-miR-449	MI0001648	UGGCAGUGUAUUGUUAGCUGGU
Plate 2	A03	C-300726-03	hsa-miR-191*	MI0000465	GCUGCGCUUGGAUUUCGUCCCC
Plate 2	A04	C-300728-03	hsa-miR-369-5p	MI0000777	AGAUCGACCGUGUUAUAUUCGC
Plate 2	A05	C-300729-03	hsa-miR-431	MI0001721	UGUCUUGCAGGCCGUCAUGCA
Plate 2	A06	C-300730-03	hsa-miR-433	MI0001723	AUCAUGAUGGGCUCCUCGGUGU
Plate 2	A07	C-300731-03	hsa-miR-329	MI0001725	AACACACCUGGUUAACCUCUUU
Plate 2	A08	C-300732-03	hsa-miR-329	MI0001726	AACACACCUGGUUAACCUCUUU
Plate 2	A09	C-300739-03	hsa-miR-412	MI0002464	ACUUCACCUGGUCCACUAGCCGU
Plate 2	A10	C-300740-03	hsa-miR-410	MI0002465	AAUAUAACACAGAUGGCCUGU
Plate 2	A11	C-300741-03	hsa-miR-376b	MI0002466	AUCAUAGAGGAAAAUCCAUGUU
Plate 2	B02	C-300743-03	hsa-miR-484	MI0002468	UCAGGCUCAGUCCCCUCCCGAU

Plate 2	B03	C-300744-03	hsa-miR-485-5p	MI0002469	AGAGGCUGGCCGUGAUGAAUUC
Plate 2	B04	C-300745-03	hsa-miR-485-3p	MI0002469	GUCAUACACGGCUCUCCUCUCU
Plate 2	B05	C-300746-03	hsa-miR-486	MI0002470	UCCUGUACUGAGCUGCCCCGAG
Plate 2	B06	C-300747-03	hsa-miR-487	MI0002471	AAUCAUACAGGGACAUCCAGUU
Plate 2	B07	C-300752-03	hsa-miR-511	MI0003127	GUGUCUUUUGCUCUGCAGUCA
Plate 2	B08	C-300753-03	hsa-miR-511	MI0003128	GUGUCUUUUGCUCUGCAGUCA
Plate 2	B09	C-300754-03	hsa-miR-146b	MI0003129	UGAGAACUGAAUUCCAUAGGCU
Plate 2	B10	C-300758-03	hsa-miR-493	MI0003132	UUGUACAUGGUAGGCUUUCAUU
Plate 2	B11	C-300759-03	hsa-miR-432	MI0003133	UCUUGGAGUAGGUCAUUGGGUGG
Plate 2	C02	C-300760-03	hsa-miR-432*	MI0003133	CUGGAUGGCUCCUCCAUGUCU
Plate 2	C03	C-300765-03	hsa-miR-497	MI0003138	CAGCAGCACACUGUGGUUUGU
Plate 2	C04	C-300767-03	hsa-miR-512-5p	MI0003140	CACUCAGCCUUGAGGGCACUUUC
Plate 2	C05	C-300768-03	hsa-miR-512-5p	MI0003141	CACUCAGCCUUGAGGGCACUUUC
Plate 2	C06	C-300769-03	hsa-miR-512-3p	MI0003140	AAGUGCUGUCAUAGCUGAGGUC
Plate 2	C07	C-300770-03	hsa-miR-512-3p	MI0003141	AAGUGCUGUCAUAGCUGAGGUC
Plate 2	C08	C-300771-03	hsa-miR-498	MI0003142	UUUCAAGCCAGGGGGCGUUUUUC
Plate 2	C09	C-300772-03	hsa-miR-520e	MI0003143	AAAGUGCUUCCUUUUUGAGGG
Plate 2	C10	C-300773-03	hsa-miR-515-5p	MI0003144	UUCUCCAAAAGAAAGCACUUUCUG
Plate 2	C11	C-300774-03	hsa-miR-515-5p	MI0003147	UUCUCCAAAAGAAAGCACUUUCUG
Plate 2	D02	C-300777-03	hsa-miR-519e*	MI0003145	UUCUCCAAAAGGGAGCACUUUC
Plate 2	D03	C-300779-03	hsa-miR-520f	MI0003146	AAGUGCUUCCUUUUAGAGGGUU
Plate 2	D04	C-300786-03	hsa-miR-519c	MI0003148	AAAGUGCAUCUUUUUAGAGGAU
Plate 2	D05	C-300787-03	hsa-miR-520a*	MI0003149	CUCCAGAGGGAAGUACUUUCU
Plate 2	D06	C-300788-03	hsa-miR-520a	MI0003149	AAAGUGCUUCCCUUUGGACUGU
Plate 2	D07	C-300792-03	hsa-miR-525	MI0003152	CUCCAGAGGGAUGCACUUUCU
Plate 2	D08	C-300797-03	hsa-miR-520b	MI0003155	AAAGUGCUUCCUUUUAGAGGG
Plate 2	D09	C-300798-03	hsa-miR-518b	MI0003156	CAAAGCGCUCCCCUUUAGAGGU
Plate 2	D10	C-300804-03	hsa-miR-518c*	MI0003159	UCUCUGGAGGGAAGCACUUUCUG
Plate 2	D11	C-300806-03	hsa-miR-524*	MI0003160	CUACAAAGGGAAGCACUUUCUC
Plate 2	E02	C-300807-03	hsa-miR-524	MI0003160	GAAGGCGCUUCCCUUUGGAGU
Plate 2	E03	C-300808-03	hsa-miR-517*	MI0003161	CCUCUAGAUGGAAGCACUGUCU
Plate 2	E04	C-300809-03	hsa-miR-517*	MI0003165	CCUCUAGAUGGAAGCACUGUCU
Plate 2	E05	C-300810-03	hsa-miR-517*	MI0003174	CCUCUAGAUGGAAGCACUGUCU
Plate 2	E06	C-300813-03	hsa-miR-521	MI0003163	AACGCACUUCCCUUUAGAGUGU
Plate 2	E07	C-300814-03	hsa-miR-521	MI0003176	AACGCACUUCCCUUUAGAGUGU
Plate 2	E08	C-300817-03	hsa-miR-517b	MI0003165	UCGUGCAUCCCUUUAGAGUGUU
Plate 2	E09	C-300818-03	hsa-miR-520g	MI0003166	ACAAAGUGCUUCCCUUUAGAGUGU
Plate 2	E10	C-300821-03	hsa-miR-516-3p	MI0003167	UGCUUCCUUUCAGAGGGU
Plate 2	E11	C-300822-03	hsa-miR-516-3p	MI0003172	UGCUUCCUUUCAGAGGGU
Plate 2	F02	C-300823-03	hsa-miR-516-3p	MI0003180	UGCUUCCUUUCAGAGGGU
Plate 2	F03	C-300824-03	hsa-miR-516-3p	MI0003181	UGCUUCCUUUCAGAGGGU
Plate 2	F04	C-300830-03	hsa-miR-518d	MI0003171	CAAAGCGCUUCCCUUUGGAGC
Plate 2	F05	C-300832-03	hsa-miR-517c	MI0003174	AUCGUGCAUCCUUUUAGAGUGU
Plate 2	F06	C-300833-03	hsa-miR-520h	MI0003175	ACAAAGUGCUUCCCUUUAGAGU
Plate 2	F07	C-300854-03	hsa-miR-299-5p	MI0000744	UGGUUUACCGUCCCACAUACAU
Plate 2	F08	C-300859-01	hsa-miR-539	MI0003514	GGAGAAAUUAUCCUUGGUGUGU
Plate 2	F09	C-300862-01	hsa-miR-487b	MI0003530	AAUCGUACAGGGUCAUCCACUU
Plate 2	F10	C-300867-01	hsa-miR-532	MI0003205	CAUGCCUUGAGUGUAGGACCGU

Plate 2	F11	C-300868-01	hsa-miR-551a	MI0003556	GCGACCCACUCUUGGUUUCCA
Plate 2	G02	C-300869-01	hsa-miR-552	MI0003557	AACAGGUGACUGGUUAGACAA
Plate 2	G03	C-300870-01	hsa-miR-553	MI0003558	AAAACGGUGAGAUUUUGUUUU
Plate 2	G04	C-300871-01	hsa-miR-554	MI0003559	GCUAGUCCUGACUCAGCCAGU
Plate 2	G05	C-300873-01	hsa-miR-555	MI0003561	AGGGUAAGCUGAACCUCUGAU
Plate 2	G06	C-300875-01	hsa-miR-557	MI0003563	GUUUGCACGGGUGGGCCUUGUCU
Plate 2	G07	C-300876-01	hsa-miR-558	MI0003564	UGAGCUGCUGUACCAAAAU
Plate 2	G08	C-300877-01	hsa-miR-559	MI0003565	UAAAGUAAAUAUGCACCAAAA
Plate 2	G09	C-300879-01	hsa-miR-561	MI0003567	CAAAGUUUAAGAUCCUUGAAGU
Plate 2	G10	C-300880-01	hsa-miR-562	MI0003568	AAAGUAGCUGUACCAUUUGC
Plate 2	G11	C-300881-01	hsa-miR-563	MI0003569	AGGUUGACAUACGUUUCCC
Plate 2	H02	C-300882-01	hsa-miR-564	MI0003570	AGGCACGGUGUCAGCAGGC
Plate 2	H03	C-300884-01	hsa-miR-566	MI0003572	GGGCGCCUGUGAUCCCAAC
Plate 2	H04	C-300885-01	hsa-miR-567	MI0003573	AGUAUGUUCUUCCAGGACAGAAC
Plate 2	H05	C-300886-01	hsa-miR-568	MI0003574	AUGUAUAAAUGUAUACACAC
Plate 2	H06	C-300887-01	hsa-miR-551b	MI0003575	GCGACCCAUACUUGGUUUCAG
Plate 2	H07	C-300888-01	hsa-miR-569	MI0003576	AGUUAAUGAAUCCUGGAAAGU
Plate 2	H08	C-300890-01	hsa-miR-571	MI0003578	UGAGUUGGCCAUCUGAGUGAG
Plate 2	H09	C-300891-01	hsa-miR-572	MI0003579	GUCCGCUCGGCGGUGGCCCA
Plate 2	H10	C-300892-01	hsa-miR-573	MI0003580	CUGAAGUGAUGUGUAACUGAUCAG
Plate 2	H11	C-300894-01	hsa-miR-575	MI0003582	GAGCCAGUUGGACAGGAGC
Plate 3	A02	C-300896-01	hsa-miR-577	MI0003584	UAGAUAAAAUAUUGGUACCUG
Plate 3	A03	C-300897-01	hsa-miR-578	MI0003585	CUUCUUGUGCUCUAGGAUUGU
Plate 3	A04	C-300899-01	hsa-miR-580	MI0003587	UUGAGAAUGAUGAAUCAUUAGG
Plate 3	A05	C-300900-01	hsa-miR-581	MI0003588	UCUUGUGUUCUCUAGAUCAGU
Plate 3	A06	C-300901-01	hsa-miR-582	MI0003589	UUACAGUUGUUCAACCAGUUACU
Plate 3	A07	C-300902-01	hsa-miR-583	MI0003590	CAAAGAGGAAGGUCCCAUUAC
Plate 3	A08	C-300903-01	hsa-miR-584	MI0003591	UUAUGGUUUGCCUGGGACUGAG
Plate 3	A09	C-300904-01	hsa-miR-585	MI0003592	UGGGCGUAUCUGUAUGCUA
Plate 3	A10	C-300905-01	hsa-miR-548a	MI0003593	CAAAACUGGCAAUUACUUUUGC
Plate 3	A11	C-300906-01	hsa-miR-586	MI0003594	UAUGCAUUGUAUUUUUAGGUCC
Plate 3	B02	C-300907-01	hsa-miR-587	MI0003595	UUUCCAUAGGUGAUGAGUCAC
Plate 3	B03	C-300908-01	hsa-miR-548b	MI0003596	CAAGAACCUCAGUUGCUUUUGU
Plate 3	B04	C-300909-01	hsa-miR-588	MI0003597	UUGGCCACAAUGGGUUAGAAC
Plate 3	B05	C-300910-01	hsa-miR-548a	MI0003598	CAAAACUGGCAAUUACUUUUGC
Plate 3	B06	C-300911-01	hsa-miR-589	MI0003599	UCAGAACAAAUGCCGGUUCCCAGA
Plate 3	B07	C-300912-01	hsa-miR-550	MI0003600	UGUCUUACUCCCUCAGGCACAU
Plate 3	B08	C-300913-01	hsa-miR-550	MI0003601	UGUCUUACUCCCUCAGGCACAU
Plate 3	B09	C-300914-01	hsa-miR-590	MI0003602	GAGCUUAUUCAUAAAAGUGCAG
Plate 3	B10	C-300915-01	hsa-miR-591	MI0003603	AGACCAUGGGUUCUCAUUGU
Plate 3	B11	C-300916-01	hsa-miR-592	MI0003604	UUGUGUCAAUAUGCGAUGAUGU
Plate 3	C02	C-300917-01	hsa-miR-593	MI0003605	AGGCACCAGCCAGGCAUUGCUCAGC
Plate 3	C03	C-300919-01	hsa-miR-595	MI0003607	GAAGUGUGCCGUGGUGUGUCU
Plate 3	C04	C-300920-01	hsa-miR-596	MI0003608	AAGCCUGCCCGGCUCCUCGGG
Plate 3	C05	C-300921-01	hsa-miR-597	MI0003609	UGUGUCACUCGAUGACCACUGU
Plate 3	C06	C-300922-01	hsa-miR-598	MI0003610	UACGUCAUCGUUGUCAUCGUCA
Plate 3	C07	C-300923-01	hsa-miR-599	MI0003611	GUUGUGUCAGUUUAUCAAAC

Plate 3	C08	C-300924-01	hsa-miR-548a	MI0003612	CAAAACUGGCAAUUACUUUUGC
Plate 3	C09	C-300925-01	hsa-miR-600	MI0003613	ACUUACAGACAAGAGCCUUGCUC
Plate 3	C10	C-300926-01	hsa-miR-601	MI0003614	UGGUCUAGGAUUGUUGGAGGAG
Plate 3	C11	C-301017-01	hsa-let-7e*	MI0000066	CUAUACGGCCUCCUAGCUUUCC
Plate 3	D02	C-301018-01	hsa-miR-15a*	MI0000069	CAGGCCAUAUUGUGCUGCCUCA
Plate 3	D03	C-301019-01	hsa-miR-16-1*	MI0000070	CCAGUAUUAACUGUGCUGCUGA
Plate 3	D04	C-301016-01	hsa-let-7c*	MI0000064	UAGAGUUACACCCUGGGAGUUA
Plate 3	D05	C-301020-01	hsa-miR-19a*	MI0000073	AGUUUUGCAUAGUUGCACUACA
Plate 3	D06	C-301021-01	hsa-miR-19b-1*	MI0000074	AGUUUUGCAGGUUUGCAUCCAGC
Plate 3	D07	C-301022-01	hsa-miR-20a*	MI0000076	ACUGCAUUAUGAGCACUUAAAG
Plate 3	D08	C-301023-01	hsa-miR-21*	MI0000077	CAACACCAGUCGAUGGGCUGU
Plate 3	D09	C-301024-01	hsa-miR-22*	MI0000078	AGUUCUUCAGUGGCAAGCUUUA
Plate 3	D10	C-301025-01	hsa-miR-23a*	MI0000079	GGGGUUCCUGGGGAUGGGAUUU
Plate 3	D11	C-301026-01	hsa-miR-24-2*	MI0000081	UGCCUACUGAGCUGAAACACAG
Plate 3	E02	C-301027-01	hsa-miR-26a-1*	MI0000083	CCUAUUCUUGGUUACUUGCACG
Plate 3	E03	C-301028-01	hsa-miR-27a*	MI0000085	AGGGCUUAGCUGCUUGUGAGCA
Plate 3	E04	C-301029-01	hsa-miR-31*	MI0000089	UGCUAUGCCAACAUAUUGCCAU
Plate 3	E05	C-301030-01	hsa-miR-92a-1*	MI0000093	AGGUUGGGAUCGGUUGCAAUGCU
Plate 3	E06	C-301031-01	hsa-miR-99a*	MI0000101	CAAGCUCGCUUCUAUGGGUCUG
Plate 3	E07	C-301032-01	hsa-miR-100*	MI0000102	CAAGCUUGUAUCUAUAGGUAUG
Plate 3	E08	C-301033-01	hsa-miR-192*	MI0000234	CUGCCAAUUCCAUAGGUCACAG
Plate 3	E09	C-301034-01	hsa-miR-30c-2*	MI0000254	CUGGGAGAAGGCUGUUUACUCU
Plate 3	E10	C-301035-01	hsa-miR-30d*	MI0000255	CUUUCAGUCAGAUGUUUGCUGC
Plate 3	E11	C-301036-01	hsa-miR-139-3p	MI0000261	GGAGACGCGGCCCUGUUGGAGU
Plate 3	F02	C-301037-01	hsa-miR-7-2*	MI0000264	CAACAAAUCCCAGUCUACCUAA
Plate 3	F03	C-301038-01	hsa-miR-10a*	MI0000266	CAAAUUCGUAUCUAGGGGAAUA
Plate 3	F04	C-300927-01	hsa-miR-602	MI0003615	GACACGGGCGACAGCUGCGGCCC
Plate 3	F05	C-300928-01	hsa-miR-603	MI0003616	CACACACUGCAAUUACUUUUGC
Plate 3	F06	C-300929-01	hsa-miR-604	MI0003617	AGGCUGCGGAAUUCAGGAC
Plate 3	F07	C-300930-01	hsa-miR-605	MI0003618	UAAAUCCCAUGGUGCCUUCUCCU
Plate 3	F08	C-300931-01	hsa-miR-606	MI0003619	AAACUACUGAAAAUCAAAGAU
Plate 3	F09	C-300932-01	hsa-miR-607	MI0003620	GUUCAAAUCCAGAUCUAUAAC
Plate 3	F10	C-300933-01	hsa-miR-608	MI0003621	AGGGGUGGUGUUGGGACAGCUCCGU
Plate 3	F11	C-300934-01	hsa-miR-609	MI0003622	AGGGUGUUUCUCUCAUCUCU
Plate 3	G02	C-300935-01	hsa-miR-610	MI0003623	UGAGCUAAAUGUGUGCUGGGA
Plate 3	G03	C-300936-01	hsa-miR-611	MI0003624	GCGAGGACCCCUCGGGGUCUGAC
Plate 3	G04	C-300937-01	hsa-miR-612	MI0003625	GCUGGGCAGGGCUUCUGAGCUCCUU
Plate 3	G05	C-300938-01	hsa-miR-613	MI0003626	AGGAAUGUUCCUUCUUUGCC
Plate 3	G06	C-300939-01	hsa-miR-614	MI0003627	GAACGCCUGUUCUUGCCAGGUGG
Plate 3	G07	C-300941-01	hsa-miR-616	MI0003629	ACUCAAAACCCUUCAGUGACUU
Plate 3	G08	C-300942-01	hsa-miR-548c	MI0003630	CAAAAAUCUCAAUUACUUUUGC
Plate 3	G09	C-300943-01	hsa-miR-617	MI0003631	AGACUUCCCAUUUGAAGGUGGC
Plate 3	G10	C-300944-01	hsa-miR-618	MI0003632	AAACUCUACUUGUCCUUCUGAGU
Plate 3	G11	C-300945-01	hsa-miR-619	MI0003633	GACCUGGACAUGUUUGUGCCCAGU
Plate 3	H02	C-300946-01	hsa-miR-620	MI0003634	AUGGAGAUAGAUAUAGAAAU
Plate 3	H03	C-300947-01	hsa-miR-621	MI0003635	GGCUAGCAACAGCGCUUACCU
Plate 3	H04	C-300948-01	hsa-miR-622	MI0003636	ACAGUCUGCUGAGGUUGGAGC
Plate 3	H05	C-300949-01	hsa-miR-623	MI0003637	AUCCCUUGCAGGGGCUGUUGGGU

Plate 3	H06	C-300950-01	hsa-miR-624	MI0003638	UAGUACCAGUACCUUGUGUUCA
Plate 3	H07	C-300952-01	hsa-miR-626	MI0003640	AGCUGUCUGAAAAUGUCUU
Plate 3	H08	C-300953-01	hsa-miR-627	MI0003641	GUGAGUCUCUAAGAAAAGAGGA
Plate 3	H09	C-300955-01	hsa-miR-629	MI0003643	GUUCUCCCAACGUAAGCCCAGC
Plate 3	H10	C-300956-01	hsa-miR-630	MI0003644	AGUAUUCUGUACCAGGGAAGGU
Plate 3	H11	C-300957-01	hsa-miR-631	MI0003645	AGACCUGGCCCAGACCUCAGC
Plate 4	A02	C-300959-01	hsa-miR-632	MI0003647	GUGUCUGCUUCCUGUGGGA
Plate 4	A03	C-300960-01	hsa-miR-633	MI0003648	CUAAUAGUAUCUACCACAAUAAA
Plate 4	A04	C-300961-01	hsa-miR-634	MI0003649	AACCAGCACCCCAACUUUGGAC
Plate 4	A05	C-300962-01	hsa-miR-635	MI0003650	ACUUGGGCACUGAAACAAUGUCC
Plate 4	A06	C-300964-01	hsa-miR-637	MI0003652	ACUGGGGGCUUUCGGGCUCUGCGU
Plate 4	A07	C-300965-01	hsa-miR-638	MI0003653	AGGGAUCGCGGGCGGGUGGCGGCCU
Plate 4	A08	C-300966-01	hsa-miR-639	MI0003654	AUCGCUGCGGUUGCGAGCGCUGU
Plate 4	A09	C-300967-01	hsa-miR-640	MI0003655	AUGAUCCAGGAACCUGCCUCU
Plate 4	A10	C-300968-01	hsa-miR-641	MI0003656	AAAGACAUAGGAUAGAGUCACCUC
Plate 4	A11	C-300969-01	hsa-miR-642	MI0003657	GUCCCUCUCCAAAUGUGUCUUG
Plate 4	B02	C-300970-01	hsa-miR-643	MI0003658	ACUUGUAUGCUAGCUCAGGUAG
Plate 4	B03	C-300971-01	hsa-miR-644	MI0003659	AGUGUGGCUUUCUUAGAGC
Plate 4	B04	C-300972-01	hsa-miR-645	MI0003660	UCUAGGCUGGUACUGCUGA
Plate 4	B05	C-300973-01	hsa-miR-646	MI0003661	AAGCAGCUGCCUCUGAGGC
Plate 4	B06	C-300974-01	hsa-miR-647	MI0003662	GUGGCUGCACUCACUUCCUUC
Plate 4	B07	C-300975-01	hsa-miR-648	MI0003663	AAGUGUGCAGGGCACUGGU
Plate 4	B08	C-300976-01	hsa-miR-649	MI0003664	AAACCUGUGUUGUUCAAGAGUC
Plate 4	B09	C-300977-01	hsa-miR-650	MI0003665	AGGAGGCAGCGCUCUCAGGAC
Plate 4	B10	C-300978-01	hsa-miR-651	MI0003666	UUUAGGAUAAGCUUGACUUUUG
Plate 4	B11	C-300980-01	hsa-miR-548d	MI0003668	CAAAAACCACAGUUUCUUUUGC
Plate 4	C02	C-300981-01	hsa-miR-661	MI0003669	UGCCUGGGUCUCUGGCCUGCGCGU
Plate 4	C03	C-300982-01	hsa-miR-662	MI0003670	UCCCACGUUGUGGCCCAGCAG
Plate 4	C04	C-300983-01	hsa-miR-548d	MI0003671	CAAAAACCACAGUUUCUUUUGC
Plate 4	C05	C-300984-01	hsa-miR-663	MI0003672	AGGCGGGGCGCCGCGGGACCGC
Plate 4	C06	C-300985-01	hsa-miR-449b	MI0003673	AGGCAGUGUAUUGUUAGCUGGC
Plate 4	C07	C-300987-01	hsa-miR-411	MI0003675	UAGUAGACCGUAUAGCGUACG
Plate 4	C08	C-300988-01	hsa-miR-654	MI0003676	UGGUGGGCCGCAGAACAUGUGC
Plate 4	C09	C-300989-01	hsa-miR-655	MI0003677	AUAAUACAUGGUUAACCUCUUU
Plate 4	C10	C-300990-01	hsa-miR-656	MI0003678	AAUAUUAUACAGUCAACCUCU
Plate 4	C11	C-300991-01	hsa-miR-549	MI0003679	UGACAACUAUGGAUGAGCUCU
Plate 4	D02	C-300992-01	hsa-miR-657	MI0003681	GGCAGGUUCUCACCCUCUCUAGG
Plate 4	D03	C-300993-01	hsa-miR-658	MI0003682	GGCGGAGGGAAGUAGGUCCGUUGGU
Plate 4	D04	C-300994-01	hsa-miR-659	MI0003683	CUUGGUUCAGGGAGGGUCCCCA
Plate 4	D05	C-300995-01	hsa-miR-660	MI0003684	UACCCAUUGCAUAUCGGAGUUG
Plate 4	D06	C-300996-01	hsa-miR-421	MI0003685	AUCAACAGACAUUAAUUGGGCGC
Plate 4	D07	C-300997-01	hsa-miR-425-5p	MI0001448	AAUGACACGAUCACUCCCGUUGA
Plate 4	D08	C-300496-05	hsa-miR-24	MI0000080	UGGCUCAGUUCAGCAGGAACAG
Plate 4	D09	C-300573-05	hsa-miR-218	MI0000294	UUGUGCUUGAUCUAACCAUGU
Plate 4	D10	C-300490-05	hsa-miR-19b	MI0000075	UGUGCAAAUCCAUGCAAAACUGA
Plate 4	D11	C-300481-05	hsa-let-7f	MI0000068	UGAGGUAGUAGAUUGUAUAGUU
Plate 4	E02	C-300475-05	hsa-let-7a	MI0000062	UGAGGUAGUAGGUUGUAUAGUU

Plate 4	E03	C-300473-05	hsa-let-7a	MI0000060	UGAGGUAGUAGGUUGUAUAGUU
Plate 4	E04	C-300576-05	hsa-miR-219	MI0000740	UGAUUGUCCAAACGCAAUUCU
Plate 4	E05	C-300727-05	hsa-miR-200a*	MI0000737	CAUCUUACCGGACAGUGCUGGA
Plate 4	E06	C-300651-05	hsa-miR-200a	MI0000737	UAACACUGUCUGGUAACGAUGU
Plate 4	E07	C-300515-05	hsa-miR-98	MI0000100	UGAGGUAGUAAGUUGUAUUGUU
Plate 4	E08	C-300587-05	hsa-miR-15b	MI0000438	UAGCAGCACAUCAUGGUUUACA
Plate 4	E09	C-300521-05	hsa-miR-29b	MI0000107	UAGCACCAUUUGAAAUCAGUGUU
Plate 4	E10	C-300520-05	hsa-miR-29b	MI0000105	UAGCACCAUUUGAAAUCAGUGUU
Plate 4	E11	C-300545-05	hsa-miR-147	MI0000262	GUGUGUGGAAAUGCUUCUGC
Plate 4	F02	C-300682-05	hsa-miR-375	MI0000783	UUUGUUCGUUCGGCUCGCGUGA
Plate 4	F03	C-300570-05	hsa-miR-215	MI0000291	AUGACCUAUGAAUUGACAGAC
Plate 4	F04	C-300589-05	hsa-miR-27b	MI0000440	UUCACAGUGGCUAAGUUCUGC
Plate 4	F05	C-300625-05	hsa-miR-126*	MI0000471	CAUUAUUACUUUUGGUACGCG
Plate 4	F06	C-300717-05	hsa-miR-424	MI0001446	CAGCAGCAAUUCAUGUUUUGAA
Plate 4	F07	C-300563-05	hsa-miR-204	MI0000284	UUCCCUUUGUCAUCCUAUGCCU
Plate 4	F08	C-300564-05	hsa-miR-205	MI0000285	UCCUUCAUUCCACCGGAGUCUG
Plate 4	F09	C-300663-05	hsa-miR-361	MI0000760	UUAUCAGAAUCUCCAGGGGUAC
Plate 4	F10	C-300673-05	hsa-miR-367	MI0000775	AAUUGCACUUUAGCAAUGGUGA
Plate 4	F11	C-300662-05	hsa-miR-30e-3p	MI0000749	CUUUCAGUCGGAUGUUUACAGC
Plate 4	G02	C-300681-05	hsa-miR-374	MI0000782	UUAUAAUACAACCUGAUAAGUG
Plate 4	G03	C-300708-05	hsa-miR-335	MI0000816	UCAAGAGCAAUAACGAAAAAUGU
Plate 4	G04	C-300703-05	hsa-miR-331	MI0000812	GCCCCUGGGCCUAUCCUAGAA
Plate 4	G05	C-300618-05	hsa-miR-9	MI0000466	UCUUUGGUUAUCUAGCUGUAUGA
Plate 4	G06	C-300578-05	hsa-miR-221	MI0000298	AGCUACAUUGUCUGCUGGGUUUC
Plate 4	G07	C-300672-05	hsa-miR-302d	MI0000774	UAAGUGCUUCCAUGUUUGAGUGU
Plate 4	G08	C-300671-05	hsa-miR-302c	MI0000773	UAAGUGCUUCCAUGUUUCAGUGG
Plate 4	G09	C-300670-05	hsa-miR-302c*	MI0000773	UUUAACAUGGGGGUACCUGCUG
Plate 4	G10	C-300669-05	hsa-miR-302b	MI0000772	UAAGUGCUUCCAUGUUUUAGUAG
Plate 4	G11	C-300653-05	hsa-miR-302a	MI0000738	UAAGUGCUUCCAUGUUUUGGUGA
Plate 4	H02	C-300840-05	hsa-miR-502	MI0003186	AUCCUUGCUAUCUGGGUGCUA
Plate 4	H03	C-300841-05	hsa-miR-503	MI0003188	UAGCAGCGGGAACAGUUCUGCAG
Plate 4	H04	C-300722-05	hsa-miR-429	MI0001641	UAAUACUGUCUGGUAAAACCGU
Plate 4	H05	C-300839-05	hsa-miR-501	MI0003185	AAUCCUUUGUCCCUGGGUGAGA
Plate 4	H06	C-300838-05	hsa-miR-500	MI0003184	AUGCACCUGGGCAAGGAUUCUG
Plate 4	H07	C-300847-05	hsa-miR-507	MI0003194	UUUUGCACCUUUUGGAGUGAA
Plate 4	H08	C-300846-05	hsa-miR-506	MI0003193	UAAGGCACCCUUCUGAGUAGA
Plate 4	H09	C-300588-05	hsa-miR-23b	MI0000439	AUCACAUUGCCAGGGAUUACC
Plate 4	H10	C-300848-05	hsa-miR-508	MI0003195	UGAUUGUAGCCUUUUGGAGUAGA
Plate 4	H11	C-300856-05	hsa-miR-455	MI0003513	UAUGUGCCUUUGGACUACAUCG
Plate 5	A02	C-300720-05	hsa-miR-20b	MI0001519	CAAAGUGCUCAUAGUGCAGGUAG
Plate 5	A03	C-300649-05	hsa-miR-106b	MI0000734	UAAAGUGCUGACAGUGCAGAU
Plate 5	A04	C-300561-05	hsa-miR-199b	MI0000282	CCCAGUGUUUAGACUAUCUGUUC
Plate 5	A05	C-300484-05	hsa-miR-16	MI0000115	UAGCAGCACGUAAAUAUUGGCG
Plate 5	A06	C-300665-05	hsa-miR-363	MI0000764	AAUUGCACGGUAUCCAUCUGUA
Plate 5	A07	C-300864-03	hsa-miR-363*	MI0000764	CGGGUGGAUCACGAUGCAAUUU
Plate 5	A08	C-300710-05	hsa-miR-325	MI0000824	CCUAGUAGGUGUCCAGUAAGUGU
Plate 5	A09	C-300531-05	hsa-miR-197	MI0000239	UUCACCACCUUCUCCACCCAGC

Plate 5	A10	C-300721-05	hsa-miR-448	MI0001637	UUGCAUAUGUAGGAUGUCCCAU
Plate 5	A11	C-300498-05	hsa-miR-25	MI0000082	CAUUGCACUUGUCUCGGUCUGA
Plate 5	B02	C-300866-03	hsa-miR-542-3p	MI0003686	UGUGACAGAUUGAUAACUGAAA
Plate 5	B03	C-300503-05	hsa-miR-28	MI0000086	AAGGAGCUCACAGUCUAUUGAG
Plate 5	B04	C-300757-05	hsa-miR-492	MI0003131	AGGACCUGCGGGACAAGAUUCUU
Plate 5	B05	C-300750-05	hsa-miR-490	MI0003125	CAACCUGGAGGACUCCAUGCUG
Plate 5	B06	C-300748-05	hsa-miR-488	MI0003123	CCCAGAUAAUGGCACUCUCAA
Plate 5	B07	C-300558-05	hsa-miR-182*	MI0000272	UGGUUCUAGACUUGCCAACUA
Plate 5	B08	C-300664-05	hsa-miR-362	MI0000762	AAUCCUUGGAACCUAGGUGUGAGU
Plate 5	B09	C-300603-05	hsa-miR-135a	MI0000453	UAUGGCUUUUUAUUCCUAUGUGA
Plate 5	B10	C-300713-05	hsa-miR-384	MI0001145	AUUCCUAGAAAUUGUUCAUA
Plate 5	B11	C-300660-05	hsa-miR-130b	MI0000748	CAGUGCAAUGAUGAAAGGGCAU
Plate 5	C02	C-300476-05	hsa-let-7b	MI0000063	UGAGGUAGUAGGUUGUGUGGUU
Plate 5	C03	C-300542-05	hsa-miR-30c	MI0000736	UGUAAACAUCCUACACUCUCAGC
Plate 5	C04	C-300641-05	hsa-miR-194	MI0000488	UGUAACAGCAACUCCAUGUGGA
Plate 5	C05	C-300538-05	hsa-miR-129	MI0000252	CUUUUUGCGGUCUGGGCUUGC
Plate 5	C06	C-300534-05	hsa-miR-199a	MI0000281	CCCAGUGUUCAGACUACCUGUUC
Plate 5	C07	C-300552-05	hsa-miR-181a	MI0000269	AACAUUCAACGCUGUCGGUGAGU
Plate 5	C08	C-300553-05	hsa-miR-181a	MI0000289	AACAUUCAACGCUGUCGGUGAGU
Plate 5	C09	C-300568-05	hsa-miR-213	MI0000289	ACCAUCGACCGUUGAUUGUACC
Plate 5	C10	C-300480-05	hsa-let-7f	MI0000067	UGAGGUAGUAGAUUGUAUAGUU
Plate 5	C11	C-300540-05	hsa-miR-148a	MI0000253	UCAGUGCACUACAGAACUUUGU
Plate 5	D02	C-300513-05	hsa-miR-95	MI0000097	UUCAACGGGUAUUUAUUGAGCA
Plate 5	D03	C-300577-05	hsa-miR-220	MI0000297	CCACACCGUAUCUGACACUUU
Plate 5	D04	C-300575-05	hsa-miR-219	MI0000296	UGAUUGUCCAAACGCAAUUCU
Plate 5	D05	C-300999-01	hsa-miR-758	MI0003757	UUUGUGACCUGGUCCACUAACC
Plate 5	D06	C-301001-01	hsa-miR-668	MI0003761	UGUCACUCGGCUCGGCCCACUAC
Plate 5	D07	C-301002-01	hsa-miR-767-5p	MI0003763	UGCACCAUGGUUGUCUGAGCAUG
Plate 5	D08	C-301003-01	hsa-miR-767-3p	MI0003763	UCUGCUCAUACCCCAUGGUUUCU
Plate 5	D09	C-301005-01	hsa-miR-454-5p	MI0003820	ACCCUAUCAAUAUUGUCUCUGC
Plate 5	D10	C-301006-01	hsa-miR-769-3p	MI0003834	CUGGGAUCUCCGGGGUCUUGGUU
Plate 5	D11	C-301007-01	hsa-miR-769-5p	MI0003834	UGAGACCUCUGGGUUCUGAGCU
Plate 5	E02	C-301008-01	hsa-miR-766	MI0003836	ACUCCAGCCCCACAGCCUCAGC
Plate 5	E03	C-301009-01	hsa-miR-802	MI0003906	CAGUAACAAAGAUUCAUCCUUGU
Plate 5	E04	C-301010-01	hsa-miR-765	MI0005116	UGGAGGAGAAGGAAGGUGAUG
Plate 5	E05	C-301011-01	hsa-miR-768-3p	MI0005117	UCACAAUGCUGACACUCAAACUGCUGAC
Plate 5	E06	C-301012-01	hsa-miR-768-5p	MI0005117	GUUGGAGGAUGAAAGUACGGAGUGAU
Plate 5	E07	C-301013-01	hsa-miR-770-5p	MI0005118	UCCAGUACCACGUGUCAGGGCCA
Plate 5	E08	C-301014-01	hsa-miR-801	MI0005202	GAUUGCUCUGCGUGCGGAAUCGAC
Plate 5	E09	C-300479-05	hsa-let-7e	MI0000066	UGAGGUAGGAGGUUGUAUAGUU
Plate 5	E10	C-300485-05	hsa-miR-17-5p	MI0000071	CAAAGUGCUUACAGUGCAGGUAG
Plate 5	E11	C-300486-05	hsa-miR-17-3p	MI0000071	ACUGCAGUGAAGGCACUUGUAG
Plate 5	F02	C-300487-05	hsa-miR-18a	MI0000072	UAAGGUGCAUCUAGUGCAGAUAG
Plate 5	F03	C-300499-05	hsa-miR-26a	MI0000083	UUCAAGUAAUCCAGGAUAGGCU
Plate 5	F04	C-300507-05	hsa-miR-31	MI0000089	AGGCAAGAUGCUGGCAUAGCU
Plate 5	F05	C-300510-05	hsa-miR-92	MI0000093	UAUUGCACUUGUCCCGGCCUGU
Plate 5	F06	C-300529-05	hsa-miR-196a	MI0000238	UAGGUAGUUUCAUGUUGUUGGG
Plate 5	F07	C-300532-05	hsa-miR-198	MI0000240	GGUCCAGAGGGGAGAUAGGUUC

Plate 5	F08	C-300535-05	hsa-miR-199a*	MI0000242	ACAGUAGUCUGCACAUUGGUUA
Plate 5	F09	C-300544-05	hsa-miR-139	MI0000261	UCUACAGUGCACGUGUCUCCAG
Plate 5	F10	C-300547-05	hsa-miR-7	MI0000264	UGGAAGACUAGUGAUUUUGUUGU
Plate 5	F11	C-300548-05	hsa-miR-7	MI0000265	UGGAAGACUAGUGAUUUUGUUGU
Plate 5	G02	C-300855-05	hsa-miR-18a*	MI0000072	ACUGCCCUAAGUGCUCCUUCUGG
Plate 5	G03	C-301039-01	hsa-miR-181c*	MI0000271	AACCAUCGACCGUUGAGUGGAC
Plate 5	G04	C-301040-01	hsa-miR-187*	MI0000274	GGCUACAACACAGGACCCGGGC
Plate 5	G05	C-301041-01	hsa-miR-196a*	MI0000279	CGGCAACAAGAAACUGCCUGAG
Plate 5	G06	C-301042-01	hsa-miR-218-2*	MI0000295	CAUGGUUCUGUCAAGCACCGCG
Plate 5	G07	C-301043-01	hsa-let-7g*	MI0000433	CUGUACAGGCCACUGCCUUGC
Plate 5	G08	C-301044-01	hsa-let-7i*	MI0000434	CUGCGCAAGCUACUGCCUUGCU
Plate 5	G09	C-301045-01	hsa-miR-30b*	MI0000441	CUGGGAGGUGGAUGUUUACUUC
Plate 5	G10	C-301046-01	hsa-miR-122*	MI0000442	AACGCCAUUAUCACACUAAAUA
Plate 5	G11	C-301047-01	hsa-miR-124*	MI0000443	CGUGUUCACAGCGGACCUUGAU
Plate 5	H02	C-301049-01	hsa-miR-124*	MI0000445	CGUGUUCACAGCGGACCUUGAU
Plate 5	H03	C-301050-01	hsa-miR-125b-1*	MI0000446	ACGGGUUAGGCUCUUGGGAGCU
Plate 5	H04	C-301051-01	hsa-miR-130a*	MI0000448	UUCACAUUGUGCUACUGUCUGC
Plate 5	H05	C-301052-01	hsa-miR-132*	MI0000449	ACCGUGGCUUUCGAUUGUUACU
Plate 5	H06	C-301053-01	hsa-miR-135a*	MI0000452	UAUAGGGAUUGGAGCCGUGGCG
Plate 5	H07	C-301054-01	hsa-miR-138-2*	MI0000455	GCUAUUUCACGACACCAGGGUU
Plate 5	H08	C-301055-01	hsa-miR-140-3p	MI0000456	UACCACAGGGUAGAACCACGG
Plate 5	H09	C-301056-01	hsa-miR-141*	MI0000457	CAUCUUCCAGUACAGUGUUGGA
Plate 5	H10	C-301057-01	hsa-miR-143*	MI0000459	GGUGCAGUGCUGCAUCUCUGGU
Plate 5	H11	C-301058-01	hsa-miR-144*	MI0000460	GGAUAUCAUCAUAUACUGUAAG
Plate 6	A02	C-301059-01	hsa-miR-145*	MI0000461	GGAUUCCUGGAAAUACUGUUCU
Plate 6	A03	C-301048-01	hsa-miR-124*	MI0000444	CGUGUUCACAGCGGACCUUGAU
Plate 6	A04	C-301060-01	hsa-miR-125a-3p	MI0000469	ACAGGUGAGGUUCUUGGGAGCC
Plate 6	A05	C-301061-01	hsa-miR-125b-2*	MI0000470	UCACAAGUCAGGCUCUUGGGAC
Plate 6	A06	C-301062-01	hsa-miR-127-5p	MI0000472	CUGAAGCUCAGAGGGCUCUGAU
Plate 6	A07	C-301063-01	hsa-miR-129-3p	MI0000473	AAGCCCUUACCCCAAAAAGCAU
Plate 6	A08	C-301065-01	hsa-miR-138-1*	MI0000476	GCUACUUCACAACACCAGGGCC
Plate 6	A09	C-301066-01	hsa-miR-146a*	MI0000477	CCUCUGAAAUUCAGUUCUUCAG
Plate 6	A10	C-301067-01	hsa-miR-150*	MI0000479	CUGGUACAGGCCUGGGGGACAG
Plate 6	A11	C-301068-01	hsa-miR-193a-5p	MI0000487	UGGGUCUUUGCGGGCGAGAUGA
Plate 6	B02	C-301069-01	hsa-miR-195*	MI0000489	CCAAUAUUGGCUGUGCUGCUCC
Plate 6	B03	C-301064-01	hsa-miR-136*	MI0000475	CAUCAUCGUCUCAAAUGAGUCU
Plate 6	B04	C-301070-01	hsa-miR-200c*	MI0000650	CGUCUUACCCAGCAGUGUUUGG
Plate 6	B05	C-301073-01	hsa-miR-34b	MI0000742	CAAUCACUAACUCCACUGCCAU
Plate 6	B06	C-301074-01	hsa-miR-34c-3p	MI0000743	AAUCACUAACCACACGGCCAGG
Plate 6	B07	C-301075-01	hsa-miR-99b*	MI0000746	CAAGCUCGUGUCUGUGGGUCCG
Plate 6	B08	C-301076-01	hsa-miR-296-3p	MI0000747	GAGGGUUGGGUGGAGGCUCUCC
Plate 6	B09	C-301077-01	hsa-miR-26a-2*	MI0000750	CCUAUUCUUGAUUACUUGUUUC
Plate 6	B10	C-301071-01	hsa-miR-155*	MI0000681	CUCCUACAUAUUAGCAUUAACA
Plate 6	B11	C-301072-01	hsa-miR-128	MI0000727	UCACAGUGAACCGGUCUCUUU
Plate 6	C02	C-301078-01	hsa-miR-371-5p	MI0000779	ACUCAAACUGUGGGGGCACU
Plate 6	C03	C-301079-01	hsa-miR-377*	MI0000785	AGAGGUUGCCCUUGGUGAAUUC
Plate 6	C04	C-301080-01	hsa-miR-379*	MI0000787	UAUGUAACAUGGUCCACUAACU

Plate 6	C05	C-301081-01	hsa-miR-340	MI0000802	UUAUAAAGCAAUGAGACUGAUU
Plate 6	C06	C-301082-01	hsa-miR-330-5p	MI0000803	UCUCUGGGCCUGUGUCUUAGGC
Plate 6	C07	C-301083-01	hsa-miR-342-5p	MI0000805	AGGGGUGCUAUCUGUGAUUGA
Plate 6	C08	C-301084-01	hsa-miR-337-5p	MI0000806	GAACGGCUUCAUACAGGAGUU
Plate 6	C09	C-301085-01	hsa-miR-323-5p	MI0000807	AGGUGGUCCGUGGCGCGUUCGC
Plate 6	C10	C-301086-01	hsa-miR-151-5p	MI0000809	UCGAGGAGCUCACAGUCUAGU
Plate 6	C11	C-301087-01	hsa-miR-148b*	MI0000811	AAGUUCUGUUAUACACUCAGGC
Plate 6	D02	C-301088-01	hsa-miR-338-5p	MI0000814	AACAAUAUCCUGGUGCUGAGUG
Plate 6	D03	C-301089-01	hsa-miR-423-5p	MI0001445	UGAGGGGCAGAGAGCGAGACUUU
Plate 6	D04	C-301090-01	hsa-miR-431*	MI0001721	CAGGUCGUCUUGCAGGGCUUCU
Plate 6	D05	C-301093-01	hsa-miR-193b*	MI0003137	CGGGGUUUUGAGGGCGAGAUGA
Plate 6	D06	C-301094-01	hsa-miR-497*	MI0003138	CAAACCACACUGUGGUGUUAGA
Plate 6	D07	C-301091-01	hsa-miR-491-3p	MI0003126	CUUAUGCAAGAUUCCCUUCUAC
Plate 6	D08	C-301092-01	hsa-miR-146b-3p	MI0003129	UGCCCUGUGGACUCAGUUCUGG
Plate 6	D09	C-301095-01	hsa-miR-519b-5p	MI0003151	CUCUAGAGGGAAGCGCUUUCUG
Plate 6	D10	C-301096-01	hsa-miR-523*	MI0003153	CUCUAGAGGGAAGCGCUUUCUG
Plate 6	D11	C-301097-01	hsa-miR-520c-5p	MI0003158	CUCUAGAGGGAAGCACUUUCUG
Plate 6	E02	C-301098-01	hsa-miR-518e*	MI0003169	CUCUAGAGGGAAGCGCUUUCUG
Plate 6	E03	C-301099-01	hsa-miR-518a-5p	MI0003170	CUGCAAAGGGAAGCCCUUUC
Plate 6	E04	C-301100-01	hsa-miR-518d-5p	MI0003171	CUCUAGAGGGAAGCACUUUCUG
Plate 6	E05	C-301101-01	hsa-miR-518a-5p	MI0003173	CUGCAAAGGGAAGCCCUUUC
Plate 6	E06	C-301104-01	hsa-miR-516a-5p	MI0003180	UUCUCGAGGAAAGAAGCACUUUC
Plate 6	E07	C-301105-01	hsa-miR-516a-5p	MI0003181	UUCUCGAGGAAAGAAGCACUUUC
Plate 6	E08	C-301102-01	hsa-miR-522*	MI0003177	CUCUAGAGGGAAGCGCUUUCUG
Plate 6	E09	C-301103-01	hsa-miR-519a*	MI0003178	CUCUAGAGGGAAGCGCUUUCUG
Plate 6	E10	C-301106-01	hsa-miR-499-3p	MI0003183	AACAUCACAGCAAGUCUGUGCU
Plate 6	E11	C-301108-01	hsa-miR-194*	MI0000732	CCAGUGGGGCUGCUGUUAUCUG
Plate 6	F02	C-301107-01	hsa-miR-483-5p	MI0002467	AAGACGGGAGGAAAGAAGGGAG
Plate 6	F03	C-301109-01	hsa-miR-532-3p	MI0003205	CCUCCCACACCCAAGGCUUGCA
Plate 6	F04	C-301111-01	hsa-miR-556-3p	MI0003562	AUAUUACCAUUAGCUCAUCUUU
Plate 6	F05	C-301112-01	hsa-miR-551b*	MI0003575	GAAAUCAAGCGUGGGUGAGACC
Plate 6	F06	C-301110-01	hsa-miR-92b*	MI0003560	AGGGACGGGACGCGGUGCAGUG
Plate 6	F07	C-301113-01	hsa-miR-574-5p	MI0003581	UGAGUGUGUGUGUGUGAGUGUGU
Plate 6	F08	C-301115-01	hsa-miR-582-3p	MI0003589	UAACUGGUUGAACAACUGAACC
Plate 6	F09	C-301116-01	hsa-miR-548b-5p	MI0003596	AAAAGUAAUUGUGGUUUUGGCC
Plate 6	F10	C-301117-01	hsa-miR-589	MI0003599	UGAGAACCACGUCUGCUCUGAG
Plate 6	F11	C-301118-01	hsa-miR-550	MI0003600	AGUGCCUGAGGGAGUAAGAGCCC
Plate 6	G02	C-301119-01	hsa-miR-550	MI0003601	AGUGCCUGAGGGAGUAAGAGCCC
Plate 6	G03	C-301120-01	hsa-miR-590-3p	MI0003602	UAAUUUUAUGUAUAAGCUAGU
Plate 6	G04	C-301121-01	hsa-miR-593	MI0003605	UGUCUCUGCUGGGGUUUCU
Plate 6	G05	C-301122-01	hsa-miR-548a-5p	MI0003612	AAAAGUAAUUGCGAGUUUUACC
Plate 6	G06	C-301123-01	hsa-miR-615-5p	MI0003628	GGGGGUCCCCGGUGCUCGGAUC
Plate 6	G07	C-301124-01	hsa-miR-616	MI0003629	AGUCAUUGGAGGGUUUGAGCAG
Plate 6	G08	C-301125-01	hsa-miR-548c-5p	MI0003630	AAAAGUAAUUGCGGUUUUUGCC
Plate 6	G09	C-301126-01	hsa-miR-624	MI0003638	CACAAGGUAUUGGUAUUACCU
Plate 6	G10	C-301127-01	hsa-miR-625*	MI0003639	GACUAUAGAACUUUCCCCCUCA
Plate 6	G11	C-301114-01	hsa-miR-576-3p	MI0003583	AAGAUGUGGAAAAAUUGGAAUC
Plate 6	H02	C-301128-01	hsa-miR-628-5p	MI0003642	AUGCUGACAUAUUUACUAGAGG

Plate 6	H03	C-301129-01	hsa-miR-629	MI0003643	UGGGUUUACGUUGGGAGAACU
Plate 6	H04	C-301130-01	hsa-miR-33b*	MI0003646	CAGUGCCUCGGCAGUGCAGCCC
Plate 6	H05	C-301131-01	hsa-miR-548d-5p	MI0003668	AAAAGUAAUUGUGGUUUUUGCC
Plate 6	H06	C-301132-01	hsa-miR-548d-5p	MI0003671	AAAAGUAAUUGUGGUUUUUGCC
Plate 6	H07	C-301133-01	hsa-miR-411*	MI0003675	UAUGUAACACGGUCCACUAACC
Plate 6	H08	C-301134-01	hsa-miR-654-3p	MI0003676	UAUGUCUGCUGACCAUCACCUU
Plate 6	H09	C-301138-01	hsa-miR-218-1*	MI0000294	AUGGUUCCGUCAAGCACCAUGG
Plate 6	H10	C-301139-01	hsa-miR-19b-2*	MI0000075	AGUUUUGCAGGUUUGCAUUUCA
Plate 6	H11	C-301140-01	hsa-let-7f-2*	MI0000068	CUAUACAGUCUACUGUCUUUCC
Plate 7	A02	C-301141-01	hsa-let-7a*	MI0000062	CUAUACAAUCUACUGUCUUUC
Plate 7	A03	C-301142-01	hsa-let-7a*	MI0000060	CUAUACAAUCUACUGUCUUUC
Plate 7	A04	C-301143-01	hsa-miR-219-2-3p	MI0000740	AGAAUUGUGGCUGGACAUCUGU
Plate 7	A05	C-301135-01	hsa-miR-101*	MI0000103	CAGUUAUCACAGUGCUGAUGCU
Plate 7	A06	C-301136-01	hsa-miR-33a*	MI0000091	CAAUGUUUCCACAGUGCAUCAC
Plate 7	A07	C-301137-01	hsa-miR-92a-2*	MI0000094	GGGUGGGGAUUUGUUGCAUUAC
Plate 7	A08	C-301145-01	hsa-miR-34a*	MI0000268	CAAUCAGCAAGUAUACUGCCCU
Plate 7	A09	C-301146-01	hsa-miR-15b*	MI0000438	CGAAUCAUUAUUUGCUGCUCUA
Plate 7	A10	C-301147-01	hsa-miR-513a-3p	MI0003191	UAAAUUUCACCUUUCUGAGAAGG
Plate 7	A11	C-301144-01	hsa-miR-200b*	MI0000342	CAUCUUACUGGGCAGCAUUGGA
Plate 7	B02	C-301148-01	hsa-miR-513a-3p	MI0003192	UAAAUUUCACCUUUCUGAGAAGG
Plate 7	B03	C-301149-01	hsa-miR-29b-2*	MI0000107	CUGGUUUCACAUGGUGGCUUAG
Plate 7	B04	C-301150-01	hsa-miR-29b-1*	MI0000105	GCUGGUUUCAUAUGGUGGUUUAGA
Plate 7	B05	C-301151-01	hsa-miR-149*	MI0000478	AGGGAGGGACGGGGGCUGUGC
Plate 7	B06	C-301152-01	hsa-miR-7-1*	MI0000263	CAACAAAUCACAGUCUGCCAUA
Plate 7	B07	C-301153-01	hsa-miR-214*	MI0000290	UGCCUGUCUACACUUGCUGUGC
Plate 7	B08	C-301155-01	hsa-miR-424*	MI0001446	CAAAACGUGAGGCGCUGCUAU
Plate 7	B09	C-301156-01	hsa-miR-26b*	MI0000084	CCUGUUCUCCAUUACUUGGCUC
Plate 7	B10	C-301157-01	hsa-miR-361-3p	MI0000760	UCCCCCAGGUGUGAUUCUGAUUU
Plate 7	B11	C-301158-01	hsa-miR-367*	MI0000775	ACUGUUGCUAAUAUGCAACUCU
Plate 7	C02	C-301154-01	hsa-miR-27b*	MI0000440	AGAGCUUAGCUGAUUGGUGAAC
Plate 7	C03	C-301159-01	hsa-miR-106a*	MI0000113	CUGCAAUGUAAGCACUUCUUAC
Plate 7	C04	C-301160-01	hsa-miR-374a*	MI0000782	CUUAUCAGAUUGUAUUGUAAUU
Plate 7	C05	C-301161-01	hsa-miR-335*	MI0000816	UUUUUCAUUAUUGCUCCUGACC
Plate 7	C06	C-301162-01	hsa-miR-331-5p	MI0000812	CUAGGUAUGGUCCCAGGGAUCC
Plate 7	C07	C-301163-01	hsa-miR-221*	MI0000298	ACCUGGCAUACAAUGUAGAUUU
Plate 7	C08	C-301164-01	hsa-miR-302d*	MI0000774	ACUUUAACAUGGAGGCACUUGC
Plate 7	C09	C-301165-01	hsa-miR-502-3p	MI0003186	AAUGCACCUGGGCAAGGAUUCA
Plate 7	C10	C-301166-01	hsa-miR-509-5p	MI0003196	UACUGCAGACAGUGGCAAUCA
Plate 7	C11	C-301167-01	hsa-miR-501-3p	MI0003185	AAUGCACCCGGGCAAGGAUUCU
Plate 7	D02	C-301168-01	hsa-miR-500	MI0003184	UAAUCCUUGCUACCUGGGUGAGA
Plate 7	D03	C-301170-01	hsa-miR-23b*	MI0000439	UGGGUUCCUGGCAUGCUGAUUU
Plate 7	D04	C-301171-01	hsa-miR-508-5p	MI0003195	UACUCCAGAGGGCGUCACUCAUG
Plate 7	D05	C-301172-01	hsa-miR-455-3p	MI0003513	GCAGUCCAUGGGCAUAUACAC
Plate 7	D06	C-301173-01	hsa-miR-10b*	MI0000267	ACAGAUUCGAUUCUAGGGGAAU
Plate 7	D07	C-301174-01	hsa-miR-20b*	MI0001519	ACUGUAGUAUGGGCACUUCCAG
Plate 7	D08	C-301175-01	hsa-miR-106b*	MI0000734	CCGCACUGUGGGUACUUGCUGC
Plate 7	D09	C-301176-01	hsa-miR-222*	MI0000299	CUCAGUAGCCAGUGUAGAUCCU

Plate 7	D10	C-301177-01	hsa-miR-199b-3p	MI0000282	ACAGUAGUCUGCACAUUGGUUA
Plate 7	D11	C-301178-01	hsa-miR-29a*	MI0000087	ACUGAUUUCUUUUGGUGUUCAG
Plate 7	E02	C-301179-01	hsa-miR-29c*	MI0000735	UGACCGAUUUCUCCUGGUGUUC
Plate 7	E03	C-301180-01	hsa-miR-16-2*	MI0000115	CCAAUAUUACUGUGCUGCUUUA
Plate 7	E04	C-301181-01	hsa-miR-32*	MI0000090	CAAUUUAGUGUGUGUGAUAUUU
Plate 7	E05	C-301169-01	hsa-miR-505*	MI0003190	GGGAGCCAGGAAGUAUUGAUGU
Plate 7	E06	C-301182-01	hsa-miR-545*	MI0003516	UCAGUAAAUGUUUAUUAGAUGA
Plate 7	E07	C-301183-01	hsa-miR-25*	MI0000082	AGGCGGAGACUUGGGCAAUUG
Plate 7	E08	C-301184-01	hsa-miR-28-3p	MI0000086	CACUAGAUUGUGAGCUCCUGGA
Plate 7	E09	C-301185-01	hsa-miR-339-3p	MI0000815	UGAGCGCCUCGACGACAGAGCCG
Plate 7	E10	C-301186-01	hsa-miR-490-5p	MI0003125	CCAUGGAUCUCCAGGUGGGU
Plate 7	E11	C-301187-01	hsa-miR-18b*	MI0001518	UGCCCUAAAUGCCCCUUCUGGC
Plate 7	F02	C-301188-01	hsa-let-7d*	MI0000065	CUAUACGACCUGCUGCCUUUCU
Plate 7	F03	C-301189-01	hsa-miR-488	MI0003123	UUGAAAGGCUAUUUCUUGGUC
Plate 7	F04	C-301190-01	hsa-miR-188-3p	MI0000484	CUCCCACAUGCAGGGUUUGCA
Plate 7	F05	C-301191-01	hsa-miR-186*	MI0000483	GCCCAAAGGUGAAUUUUUUGGG
Plate 7	F06	C-301192-01	hsa-miR-185*	MI0000482	AGGGGCUGGCUUUCCUCUGGUC
Plate 7	F07	C-301193-01	hsa-miR-183*	MI0000273	GUGAAUUACCGAAGGGCCAUAA
Plate 7	F08	C-301194-01	hsa-miR-362-3p	MI0000762	AACACACCUAUUCAAGGAUUCA
Plate 7	F09	C-301195-01	hsa-miR-105*	MI0000111	ACGGAUGUUUGAGCAUGUGCUA
Plate 7	F10	C-301196-01	hsa-miR-130b*	MI0000748	ACUCUUUCCCUGUUGCACUAC
Plate 7	F11	C-301197-01	hsa-miR-223*	MI0000300	CGUGUAUUUGACAAGCUGAGUU
Plate 7	G02	C-301198-01	hsa-let-7b*	MI0000063	CUAUACAACCUACUGCCUUCCC
Plate 7	G03	C-301199-01	hsa-miR-30c-1*	MI0000736	CUGGGAGAGGGUUGUUUACUCC
Plate 7	G04	C-301200-01	hsa-miR-135b*	MI0000810	AUGUAGGGCUAAAAGCCAUGGG
Plate 7	G05	C-301201-01	hsa-miR-129*	MI0000252	AAGCCCUUACCCCAAAAAGUAU
Plate 7	G06	C-301202-01	hsa-miR-181a-2*	MI0000269	ACCACUGACCGUUGACUGUACC
Plate 7	G07	C-301203-01	hsa-miR-105*	MI0000112	ACGGAUGUUUGAGCAUGUGCUA
Plate 7	G08	C-301205-01	hsa-miR-148a*	MI0000253	AAAGUUCUGAGACACUCCGACU
Plate 7	G09	C-301206-01	hsa-miR-96*	MI0000098	AAUCAUGUGCAGUGCCAAUAUG
Plate 7	G10	C-301015-03	hsa-miR-675	MI0005416	UGGUGCGGAGAGGGCCCACAGUG
Plate 7	G11	C-301210-01	hsa-miR-297	MI0005775	AUGUAUGUGUGCAUGUGCAUG
Plate 7	H02	C-301211-01	hsa-miR-486-3p	MI0002470	CGGGGCAGCUCAGUACAGGAU
Plate 7	H03	C-301212-01	hsa-miR-298	MI0005523	AGCAGAAGCAGGGAGGUUCUCCCA
Plate 7	H04	C-301213-01	hsa-miR-891a	MI0005524	UGCAACGAACCUGAGCCACUGA
Plate 7	H05	C-301214-01	hsa-miR-300	MI0005525	UAUACAAGGGCAGACUCUCUCU
Plate 7	H06	C-301215-01	hsa-miR-886-3p	MI0005527	CGCGGGUGCUUACUGACCCUU
Plate 7	H07	C-301216-01	hsa-miR-886-5p	MI0005527	CGGGUCGGAGUUAGCUCAAGCGG
Plate 7	H08	C-301217-01	hsa-miR-892a	MI0005528	CACUGUGUCCUUUCUGCGUAG
Plate 7	H09	C-301218-01	hsa-miR-220b	MI0005529	CCACCACCGUGUCUGACACUU
Plate 7	H10	C-301219-01	hsa-miR-509-5p	MI0005530	UACUGCAGACAGUGGCAAUCA
Plate 7	H11	C-301220-01	hsa-miR-509-3p	MI0005530	UGAUUGGUACGUCUGUGGGUAG
Plate 8	A02	C-301221-01	hsa-miR-450b-5p	MI0005531	UUUUGCAAUAUGUUCCUGAAUA
Plate 8	A03	C-301222-01	hsa-miR-450b-3p	MI0005531	UUGGGAUCAUUUUGCAUCCAUA
Plate 8	A04	C-301223-01	hsa-miR-874	MI0005532	CUGCCCUGGCCCGAGGGACCGA
Plate 8	A05	C-301224-01	hsa-miR-890	MI0005533	UACUUGGAAAGGCAUCAGUUG
Plate 8	A06	C-301225-01	hsa-miR-891b	MI0005534	UGCAACUUACCUGAGUCAUUGA

Plate 8	A07	C-301226-01	hsa-miR-220c	MI0005536	ACACAGGGCUGUUGUGAAGACU
Plate 8	A08	C-301227-01	hsa-miR-888*	MI0005537	GACUGACACCUCUUUGGGUGAA
Plate 8	A09	C-301228-01	hsa-miR-888	MI0005537	UACUCAAAAAGCUGUCAGUCA
Plate 8	A10	C-301229-01	hsa-miR-892b	MI0005538	CACUGGCUCCUUUCUGGGUAGA
Plate 8	A11	C-301230-01	hsa-miR-541	MI0005539	UGGUGGGCACAGAAUCUGGACU
Plate 8	B02	C-301231-01	hsa-miR-541*	MI0005539	AAAGGAUUCUGCUGUCGGUCCCACU
Plate 8	B03	C-301232-01	hsa-miR-889	MI0005540	UUAAUAUCGGACAACCAUUGU
Plate 8	B04	C-301233-01	hsa-miR-875-5p	MI0005541	UAUACCUCAGUUUUAUCAGGUG
Plate 8	B05	C-301234-01	hsa-miR-875-3p	MI0005541	CCUGGAAACACUGAGGUUGUG
Plate 8	B06	C-301235-01	hsa-miR-876-5p	MI0005542	UGGAUUUCUUUGUGAAUCACCA
Plate 8	B07	C-301236-01	hsa-miR-876-3p	MI0005542	UGGUGGUUUACAAAGUAAUUCA
Plate 8	B08	C-301237-01	hsa-miR-708*	MI0005543	CAACUAGACUGUGAGCUUCUAG
Plate 8	B09	C-301238-01	hsa-miR-708	MI0005543	AAGGAGCUUACAAUCUAGCUGGG
Plate 8	B10	C-301239-01	hsa-miR-147b	MI0005544	GUGUGCGGAAAUGCUUCUGCUA
Plate 8	B11	C-301240-01	hsa-miR-190b	MI0005545	UGAUAUGUUUGAUAUUGGGUU
Plate 8	C02	C-301241-01	hsa-miR-744*	MI0005559	CUGUUGCCACUAACCUCAACCU
Plate 8	C03	C-301242-01	hsa-miR-744	MI0005559	UGCGGGGCUAGGGCUAACAGCA
Plate 8	C04	C-301243-01	hsa-miR-885-5p	MI0005560	UCCAUUACACUACCCUGCCUCU
Plate 8	C05	C-301244-01	hsa-miR-885-3p	MI0005560	AGGCAGCGGGGUGUAGUGGAUA
Plate 8	C06	C-301245-01	hsa-miR-887	MI0005562	GUGAACGGGCGCCAUCCCGAGG
Plate 8	C07	C-301246-01	hsa-miR-665	MI0005563	ACCAGGAGGCUGAGGCCCCU
Plate 8	C08	C-301247-01	hsa-miR-873	MI0005564	GCAGGAACUUGUGAGUCUCCU
Plate 8	C09	C-301248-01	hsa-miR-543	MI0005565	AAACAUUCGCGGUGCACUUCUU
Plate 8	C10	C-301249-01	hsa-miR-374b*	MI0005566	CUUAGCAGGUUGUAUUAUCAUU
Plate 8	C11	C-301250-01	hsa-miR-374b	MI0005566	AUAUAAUACAACCUGCUAAGUG
Plate 8	D02	C-301251-01	hsa-miR-760	MI0005567	CGGCUCUGGGUCUGUGGGGA
Plate 8	D03	C-301252-01	hsa-miR-301b	MI0005568	CAGUGCAAUGAUAUUGUCAAAGC
Plate 8	D04	C-301207-01	hsa-miR-93*	MI0000095	ACUGCUGAGCUAGCACUUCCCG
Plate 8	D05	C-301208-01	hsa-miR-219-1-3p	MI0000296	AGAGUUGAGUCUGGACGUCCCG
Plate 8	D06	C-301209-01	hsa-miR-671-3p	MI0003760	UCCGGUUCUCAGGGCUCCACC
Plate 8	D07	C-301204-01	hsa-let-7f-1*	MI0000067	CUAUACAAUCUAUUGCCUUCCC
Plate 8	D08	C-300500-05	hsa-miR-26a	MI0000750	UUCAAGUAAUCCAGGAUAGGCU
Plate 8	D09	C-300519-05	hsa-miR-101	MI0000739	UACAGUACUGUGAUAACUGAA
Plate 8	D10	C-300530-05	hsa-miR-196a	MI0000279	UAGGUAGUUUCAUGUUGUUGGG
Plate 8	D11	C-300560-05	hsa-miR-187	MI0000274	UCGUGUCUUGUGUUGCAGCCGG
Plate 8	E02	C-300583-05	hsa-let-7g	MI0000433	UGAGGUAGUAGUUUGUACAGUU
Plate 8	E03	C-300584-05	hsa-let-7i	MI0000434	UGAGGUAGUAGUUUGUGCUGUU
Plate 8	E04	C-300585-05	hsa-miR-1	MI0000437	UGGAAUGUAAAGAAGUAUGUAU
Plate 8	E05	C-300586-05	hsa-miR-1	MI0000651	UGGAAUGUAAAGAAGUAUGUAU
Plate 8	E06	C-300591-05	hsa-miR-122a	MI0000442	UGGAGUGUGACAAUGGUGUUUG
Plate 8	E07	C-300592-05	hsa-miR-124a	MI0000443	UAAGGCACGCGGUGAAUGCC
Plate 8	E08	C-300593-05	hsa-miR-124a	MI0000444	UAAGGCACGCGGUGAAUGCC
Plate 8	E09	C-300594-05	hsa-miR-124a	MI0000445	UAAGGCACGCGGUGAAUGCC
Plate 8	E10	C-300600-05	hsa-miR-133a	MI0000450	UUUGGUCCCCUUCAACCAGCUG
Plate 8	E11	C-300601-05	hsa-miR-133a	MI0000451	UUUGGUCCCCUUCAACCAGCUG
Plate 8	F02	C-300605-05	hsa-miR-138	MI0000455	AGCUGGUGUUGUGAAUCAGGCCG
Plate 8	F03	C-300606-05	hsa-miR-138	MI0000476	AGCUGGUGUUGUGAAUCAGGCCG
Plate 8	F04	C-300607-05	hsa-miR-140	MI0000456	CAGUGGUUUUACCCUAUGGUAG

Plate 8	F05	C-300609-05	hsa-miR-142-5p	MI0000458	CAUAAAGUAGAAAGCACUACU
Plate 8	F06	C-300611-05	hsa-miR-143	MI0000459	UGAGAUGAAGCACUGUAGCUC
Plate 8	F07	C-300612-05	hsa-miR-144	MI0000460	UACAGUAUAGAUGAUGUACU
Plate 8	F08	C-300613-05	hsa-miR-145	MI0000461	GUCCAGUUUUCCCAGGAAUCCCU
Plate 8	F09	C-300614-05	hsa-miR-152	MI0000462	UCAGUGCAUGACAGAACUUGG
Plate 8	F10	C-300617-05	hsa-miR-191	MI0000465	CAACGGAAUCCCAAAAGCAGCUG
Plate 8	F11	C-300622-05	hsa-miR-9*	MI0000467	AUAAAGCUAGAUAACCGAAAGU
Plate 8	G02	C-300623-05	hsa-miR-9*	MI0000468	AUAAAGCUAGAUAACCGAAAGU
Plate 8	G03	C-300624-05	hsa-miR-125a	MI0000469	UCCCUGAGACCCUUUAACCUGUGA
Plate 8	G04	C-300628-05	hsa-miR-134	MI0000474	UGUGACUGGUUGACCAGAGGGG
Plate 8	G05	C-300640-05	hsa-miR-193a	MI0000487	AACUGGCCUACAAAGUCCCAGU
Plate 8	G06	C-300645-05	hsa-miR-320	MI0000542	AAAAGCUGGGUUGAGAGGGCGA
Plate 8	G07	C-300646-05	hsa-miR-200c	MI0000650	UAAUACUGCCGGGUAAUGAUGGA
Plate 8	G08	C-300647-05	hsa-miR-155	MI0000681	UUAAUGCUAAUCGUGAUAGGGGU
Plate 8	G09	C-300674-05	hsa-miR-368	MI0000776	AACAUAGAGGAAAUUCCACGU
Plate 8	G10	C-300676-05	hsa-miR-370	MI0000778	GCCUGCUGGGGUGGAACCUGGU
Plate 8	G11	C-300677-05	hsa-miR-371	MI0000779	AAGUGCCGCCAUCUUUUGAGUGU
Plate 8	H02	C-300686-05	hsa-miR-422b	MI0000786	ACUGGACUUGGAGUCAGAAGG
Plate 8	H03	C-300687-05	hsa-miR-379	MI0000787	UGGUAGACUAUGGAACGUAGG
Plate 8	H04	C-300693-05	hsa-miR-340	MI0000802	UCCGUCUCAGUUACUUUAUAGC
Plate 8	H05	C-300696-05	hsa-miR-342	MI0000805	UCUCACACAGAAAUCGCACCCGU
Plate 8	H06	C-300697-05	hsa-miR-337	MI0000806	CUCCUAUAUGAUGCCUUUCUUC
Plate 8	H07	C-300698-05	hsa-miR-323	MI0000807	CACAUUACACGGUCGACCUCU
Plate 8	H08	C-300700-05	hsa-miR-151	MI0000809	CUAGACUGAAGCUCCUUGAGG
Plate 8	H09	C-300705-05	hsa-miR-324-3p	MI0000813	ACUGCCCCAGGUGCUGCUGG
Plate 8	H10	C-300706-05	hsa-miR-338	MI0000814	UCCAGCAUCAGUGAUUUUGUUG
Plate 8	H11	C-300709-05	hsa-miR-133b	MI0000822	UUUGGUCCCCUUCAACCAGCUA
Plate 9	A02	C-300711-05	hsa-miR-345	MI0000825	GCUGACUCCUAGUCCAGGGCUC
Plate 9	A03	C-300715-05	hsa-miR-422a	MI0001444	ACUGGACUUAGGGUCAGAAGGC
Plate 9	A04	C-300716-05	hsa-miR-423	MI0001445	AGCUCGGUCUGAGGCCCCUCAGU
Plate 9	A05	C-300733-05	hsa-miR-453	MI0001727	AGGUUGUCCGUGGUGAGUUCGCA
Plate 9	A06	C-300737-05	hsa-miR-409-5p	MI0001735	AGGUUACCCGAGCAACUUUGCAU
Plate 9	A07	C-300738-05	hsa-miR-409-3p	MI0001735	GAAUGUUGCUCGGUGAACCCCU
Plate 9	A08	C-300742-05	hsa-miR-483	MI0002467	UCACUCCUCUCCUCCCGUCUU
Plate 9	A09	C-300751-05	hsa-miR-491	MI0003126	AGUGGGGAACCCUUCCAUGAGG
Plate 9	A10	C-300761-05	hsa-miR-494	MI0003134	UGAAACAUACACGGGAAACCUC
Plate 9	A11	C-300762-05	hsa-miR-495	MI0003135	AAACAAACAUGGUGCACUUCUU
Plate 9	B02	C-300763-05	hsa-miR-496	MI0003136	UGAGUAUUACAUGGCCAAUCUC
Plate 9	B03	C-300764-05	hsa-miR-193b	MI0003137	AACUGGCCCUCAAAGUCCCGCU
Plate 9	B04	C-300766-05	hsa-miR-181d	MI0003139	AACAUUCAUUGUUGUCGGUGGGU
Plate 9	B05	C-300775-05	hsa-miR-515-3p	MI0003144	GAGUGCCUUCUUUUGGAGCGUU
Plate 9	B06	C-300776-05	hsa-miR-515-3p	MI0003147	GAGUGCCUUCUUUUGGAGCGUU
Plate 9	B07	C-300778-05	hsa-miR-519e	MI0003145	AAGUGCCUCCUUUUAGAGUGUU
Plate 9	B08	C-300780-05	hsa-miR-526c	MI0003148	CUCUAGAGGGAAGCGCUUUCUG
Plate 9	B09	C-300789-05	hsa-miR-526b	MI0003150	CUCUUGAGGGAAGCACUUUCUGU
Plate 9	B10	C-300790-05	hsa-miR-526b*	MI0003150	GAAAGUGCUUCCUUUUAGAGGC
Plate 9	B11	C-300791-05	hsa-miR-519b	MI0003151	AAAGUGCAUCCUUUUAGAGGUU

Plate 9	C02	C-300793-05	hsa-miR-525*	MI0003152	GAAGGCGCUUCCCUUUAGAGCG
Plate 9	C03	C-300794-05	hsa-miR-523	MI0003153	GAACGCGCUUCCCUAUAGAGGGU
Plate 9	C04	C-300795-05	hsa-miR-518f*	MI0003154	CUCUAGAGGGAAGCACUUUCUC
Plate 9	C05	C-300796-05	hsa-miR-518f	MI0003154	GAAAGCGCUUCUCUUUAGAGG
Plate 9	C06	C-300799-05	hsa-miR-526a	MI0003157	CUCUAGAGGGAAGCACUUUCUG
Plate 9	C07	C-300801-05	hsa-miR-526a	MI0003168	CUCUAGAGGGAAGCACUUUCUG
Plate 9	C08	C-300803-05	hsa-miR-520c	MI0003158	AAAGUGCUUCCUUUUAGAGGGU
Plate 9	C09	C-300805-05	hsa-miR-518c	MI0003159	CAAAGCGCUUCUCUUUAGAGUGU
Plate 9	C10	C-300811-05	hsa-miR-517a	MI0003161	AUCGUGCAUCCCUUUAGAGUGU
Plate 9	C11	C-300812-05	hsa-miR-519d	MI0003162	CAAAGUGCCUCCCUUUAGAGUG
Plate 9	D02	C-300815-05	hsa-miR-520d*	MI0003164	CUACAAAGGGAAGCCCUUUC
Plate 9	D03	C-300816-05	hsa-miR-520d	MI0003164	AAAGUGCUUCUCUUUGGUGGGU
Plate 9	D04	C-300819-05	hsa-miR-516-5p	MI0003167	AUCUGGAGGUAAGAAGCACUUU
Plate 9	D05	C-300820-05	hsa-miR-516-5p	MI0003172	AUCUGGAGGUAAGAAGCACUUU
Plate 9	D06	C-300825-05	hsa-miR-518e	MI0003169	AAAGCGCUUCCCUUCAGAGUG
Plate 9	D07	C-300827-05	hsa-miR-527	MI0003179	CUGCAAAGGGAAGCCCUUUC
Plate 9	D08	C-300828-05	hsa-miR-518a	MI0003170	GAAAGCGCUUCCCUUUGCUGGA
Plate 9	D09	C-300829-05	hsa-miR-518a	MI0003173	GAAAGCGCUUCCCUUUGCUGGA
Plate 9	D10	C-300834-05	hsa-miR-522	MI0003177	AAAAUGGUUCCCUUUAGAGUGU
Plate 9	D11	C-300835-05	hsa-miR-519a	MI0003178	AAAGUGCAUCCUUUUAGAGUGU
Plate 9	E02	C-300836-05	hsa-miR-519a	MI0003182	AAAGUGCAUCCUUUUAGAGUGU
Plate 9	E03	C-300837-05	hsa-miR-499	MI0003183	UUAAGACUUGCAGUGAUGUUU
Plate 9	E04	C-300858-03	hsa-miR-493-3p	MI0003132	UGAAGGUCUACUGUGUGCCAGG
Plate 9	E05	C-300860-03	hsa-miR-544	MI0003515	AUUCUGCAUUUUUAGCAAGUUC
Plate 9	E06	C-300865-03	hsa-miR-376a*	MI0000784	GUAGAUUCUCCUUCUAUGAGUA
Plate 9	E07	C-300851-07	hsa-miR-514	MI0003198	AUUGACACUUCUGUGAGUAGA
Plate 9	E08	C-300852-07	hsa-miR-514	MI0003199	AUUGACACUUCUGUGAGUAGA
Plate 9	E09	C-300853-07	hsa-miR-514	MI0003200	AUUGACACUUCUGUGAGUAGA
Plate 9	E10	C-300872-03	hsa-miR-92b	MI0003560	UAUUGCACUCGUCCCGGCCUCC
Plate 9	E11	C-300874-03	hsa-miR-556	MI0003562	GAUGAGCUCAUUGUAAUAUGAG
Plate 9	F02	C-300889-03	hsa-miR-570	MI0003577	CGAAAACAGCAAUUACCUUUGC
Plate 9	F03	C-300893-03	hsa-miR-574	MI0003581	CACGCUCAUGCACACACCCACA
Plate 9	F04	C-300895-03	hsa-miR-576	MI0003583	AUUCUAAUUUCUCCACGUCUUU
Plate 9	F05	C-300898-03	hsa-miR-579	MI0003586	UUCAUUUGGUAUAAACCGCGAUU
Plate 9	F06	C-300940-03	hsa-miR-615	MI0003628	UCCGAGCCUGGGUCUCCCUCUU
Plate 9	F07	C-300951-03	hsa-miR-625	MI0003639	AGGGGGAAAGUUCUAUAGUCC
Plate 9	F08	C-300954-03	hsa-miR-628	MI0003642	UCUAGUAAGAGUGGCAGUCGA
Plate 9	F09	C-300958-03	hsa-miR-33b	MI0003646	GUGCAUUGCUGUUGCAUUGC
Plate 9	F10	C-300963-03	hsa-miR-636	MI0003651	UGUGCUUGCUCGUCCCGCCCGCA
Plate 9	F11	C-300979-03	hsa-miR-652	MI0003667	AAUGGCGCCACUAGGGUUGUG
Plate 9	G02	C-300986-03	hsa-miR-653	MI0003674	GUGUUGAAACAAUCUCUACUG
Plate 9	G03	C-300718-07	hsa-miR-425	MI0001448	AUCGGGAAUGUCGUGUCCGCCC
Plate 9	G04	C-300518-07	hsa-miR-101	MI0000103	UACAGUACUGUGAUAACUGAA
Plate 9	G05	C-300509-07	hsa-miR-33	MI0000091	GUGCAUUGUAGUUGCAUUGCA
Plate 9	G06	C-300511-07	hsa-miR-92	MI0000094	UAUUGCACUUGUCCCGGCCUGU
Plate 9	G07	C-300495-07	hsa-miR-189	MI0000080	UGCCUACUGAGCUGAUAUCAGU
Plate 9	G08	C-300725-07	hsa-miR-450	MI0003187	UUUUGCGAUGUGUUCCUAAUAU
Plate 9	G09	C-300724-07	hsa-miR-450	MI0001652	UUUUGCGAUGUGUUCCUAAUAU

Plate 9	G10	C-300582-07	hsa-miR-200b	MI0000342	UAAUACUGCCUGGUAAUGAUGA
Plate 9	G11	C-300551-07	hsa-miR-34a	MI0000268	UGGCAGUGUCUUAGCUGGUUGU
Plate 9	H02	C-300844-07	hsa-miR-513	MI0003191	UUCACAGGGAGGUGUCAU
Plate 9	H03	C-300845-07	hsa-miR-513	MI0003192	UUCACAGGGAGGUGUCAU
Plate 9	H04	C-300631-07	hsa-miR-149	MI0000478	UCUGGCUCCGUGUCUUCACUCCC
Plate 9	H05	C-300546-07	hsa-miR-7	MI0000263	UGGAAGACUAGUGAUUUUGUUGU
Plate 9	H06	C-300569-07	hsa-miR-214	MI0000290	ACAGCAGGCACAGACAGGCAGU
Plate 9	H07	C-300572-07	hsa-miR-217	MI0000293	UACUGCAUCAGGAACUGAUUGGA
Plate 9	H08	C-300626-07	hsa-miR-126	MI0000471	UCGUACCGUGAGUAAUAAUGCG
Plate 9	H09	C-300756-07	hsa-miR-202	MI0003130	AGAGGUAUAGGGCAUGGGAA
Plate 9	H10	C-300755-07	hsa-miR-202*	MI0003130	UUCCUAUGCAUAUACUUCUUUG
Plate 9	H11	C-300850-07	hsa-miR-510	MI0003197	UACUCAGGAGAGUGGCAAUCAC
Plate 10	A02	C-300501-07	hsa-miR-26b	MI0000084	UUCAAGUAAUUCAGGAUAGGU
Plate 10	A03	C-300604-07	hsa-miR-137	MI0000454	UUAUUGCUUAAGAAUACGCGUAG
Plate 10	A04	C-300526-07	hsa-miR-106a	MI0000113	AAAAGUGCUUACAGUGCAGGUAG
Plate 10	A05	C-300661-07	hsa-miR-30e-5p	MI0000749	UGUAAACAUCCUUGACUGGAAG
Plate 10	A06	C-300621-07	hsa-miR-9*	MI0000466	AUAAAGCUAGAUAACCGAAAGU
Plate 10	A07	C-300668-07	hsa-miR-302b*	MI0000772	ACUUUAACAUGGAAGUGCUUUC
Plate 10	A08	C-300652-07	hsa-miR-302a*	MI0000738	ACUUAAACGUGGAUGUACUUGCU
Plate 10	A09	C-300849-07	hsa-miR-509	MI0003196	UGAUUGGUACGUCUGUGGGUAG
Plate 10	A10	C-300843-07	hsa-miR-505	MI0003190	CGUCAACACUUGCUGGUUUCCU
Plate 10	A11	C-300842-07	hsa-miR-504	MI0003189	AGACCCUGGUCUGCACUCUAUC
Plate 10	B02	C-300550-07	hsa-miR-10b	MI0000267	UACCCUGUAGAACCGAAUUUGUG
Plate 10	B03	C-300571-07	hsa-miR-216	MI0000292	UAAUCUCAGCUGGCAACUGUGA
Plate 10	B04	C-300581-07	hsa-miR-224	MI0000301	CAAGUCACUAGUGGUUCCGUU
Plate 10	B05	C-300579-07	hsa-miR-222	MI0000299	AGCUACAUCUGGCUACUGGGU
Plate 10	B06	C-300504-07	hsa-miR-29a	MI0000087	UAGCACCAUCUGAAAUCGGUUA
Plate 10	B07	C-300650-07	hsa-miR-29c	MI0000735	UAGCACCAUUUGAAAUCGGUUA
Plate 10	B08	C-300508-07	hsa-miR-32	MI0000090	UAUUGCACAUUACUAAGUUGCA
Plate 10	B09	C-300735-07	hsa-miR-452	MI0001733	AACUGUUUGCAGAGGAAACUGA
Plate 10	B10	C-300736-07	hsa-miR-452*	MI0001733	CUCAUCUGCAAAGAAGUAAGUG
Plate 10	B11	C-300861-05	hsa-miR-545	MI0003516	UCAGCAAACAUUUAUUGUGUGC
Plate 10	C02	C-300863-05	hsa-miR-542-5p	MI0003686	UCGGGGAUCAUCAUGUCACGAGA
Plate 10	C03	C-300707-07	hsa-miR-339	MI0000815	UCCCUGUCCUCCAGGAGCUCACG
Plate 10	C04	C-300719-07	hsa-miR-18b	MI0001518	UAAGGUGCAUCUAGUGCAGUUAG
Plate 10	C05	C-300478-07	hsa-let-7d	MI0000065	AGAGGUAGUAGGUUGCAUAGUU
Plate 10	C06	C-300749-07	hsa-miR-489	MI0003124	GUGACAUCACAUAUACGGCAGC
Plate 10	C07	C-300638-07	hsa-miR-188	MI0000484	CAUCCCUUGCAUGGUGGAGGG
Plate 10	C08	C-300637-07	hsa-miR-186	MI0000483	CAAAGAAUUCUCCUUUUGGGCU
Plate 10	C09	C-300636-07	hsa-miR-185	MI0000482	UGGAGAGAAAGGCAGUUCCUGA
Plate 10	C10	C-300559-07	hsa-miR-183	MI0000273	UAUGGCACUGGUAGAAUUCACU
Plate 10	C11	C-300557-07	hsa-miR-182	MI0000272	UUUGGCAAUGGUAGAACUCACACU
Plate 10	D02	C-300615-07	hsa-miR-153	MI0000463	UUGCAUAGUCACAAAAGUGAUC
Plate 10	D03	C-300616-07	hsa-miR-153	MI0000464	UUGCAUAGUCACAAAAGUGAUC
Plate 10	D04	C-300524-07	hsa-miR-105	MI0000111	UCAAAUGCUCAGACUCCUGUGGU
Plate 10	D05	C-300580-07	hsa-miR-223	MI0000300	UGUCAGUUUGUCAAAUACCCCA
Plate 10	D06	C-300701-07	hsa-miR-135b	MI0000810	UAUGGCUUUUCAUUCCUAUGUGA

Plate 10	D07	C-300714-07	hsa-miR-196b	MI0001150	UAGGUAGUUUCCUGUUGUUGGG
Plate 10	D08	C-300536-07	hsa-miR-199a*	MI0000281	ACAGUAGUCUGCACAUUGGUUA
Plate 10	D09	C-300554-07	hsa-miR-181b	MI0000270	AACAUUCAUUGCUGUCGGUGGGU
Plate 10	D10	C-300555-07	hsa-miR-181b	MI0000683	AACAUUCAUUGCUGUCGGUGGGU
Plate 10	D11	C-300525-07	hsa-miR-105	MI0000112	UCAAAUGCUCAGACUCCUGUGGU
Plate 10	E02	C-300597-07	hsa-miR-128a	MI0000447	UCACAGUGAACCGGUCUCUUU
Plate 10	E03	C-300514-07	hsa-miR-96	MI0000098	UUUGGCACUAGCACAUUUUUGCU
Plate 10	E04	C-300512-07	hsa-miR-93	MI0000095	CAAAGUGCUGUUCGUGCAGGUAG
Plate 10	E05	C-301000-03	hsa-miR-671	MI0003760	AGGAAGCCCUGGAGGGGCUGGAG
Plate 10	E06	C-301004-03	hsa-miR-454-3p	MI0003820	UAGUGCAAUAUUGCUUAUAGGGU
Plate 10	E07	C-301253-01	hsa-miR-216b	MI0005569	AAAUCUCUGCAGGCAAAUGUGA
Plate 10	E08	C-301254-01	hsa-miR-208b	MI0005570	AUAAGACGAACAAAAGGUUUGU
Plate 10	E09	C-301255-01	hsa-miR-920	MI0005712	GGGGAGCUGUGGAAGCAGUA
Plate 10	E10	C-301256-01	hsa-miR-921	MI0005713	CUAGUGAGGGACAGAACCAGGAUUC
Plate 10	E11	C-301257-01	hsa-miR-922	MI0005714	GCAGCAGAGAAUAGGACUACGUC
Plate 10	F02	C-301258-01	hsa-miR-923	MI0005715	GUCAGCGGAGGAAAAGAAACU
Plate 10	F03	C-301259-01	hsa-miR-924	MI0005716	AGAGUCUUGUGAUGUCUUGC
Plate 10	F04	C-301260-01	hsa-miR-509-3-5p	MI0005717	UACUGCAGACGUGGCAAUCAUG
Plate 10	F05	C-301261-01	hsa-miR-509-3p	MI0005717	UGAUUGGUACGUCUGUGGGUAG
Plate 10	F06	C-301262-01	hsa-miR-933	MI0005755	UGUGCGCAGGGAGACCUCUCCC
Plate 10	F07	C-301263-01	hsa-miR-934	MI0005756	UGUCUACUACUGGAGACACUGG
Plate 10	F08	C-301264-01	hsa-miR-935	MI0005757	CCAGUUACCGCUUCCGCUACCGC
Plate 10	F09	C-301265-01	hsa-miR-936	MI0005758	ACAGUAGAGGGAGGAAUCGCAG
Plate 10	F10	C-301266-01	hsa-miR-937	MI0005759	AUCCGCGCUCUGACUCUCUGCC
Plate 10	F11	C-301267-01	hsa-miR-938	MI0005760	UGCCCUUAAAGGUGAACCCAGU
Plate 10	G02	C-301268-01	hsa-miR-939	MI0005761	UGGGGAGCUGAGGCUCUGGGGGUG
Plate 10	G03	C-301269-01	hsa-miR-940	MI0005762	AAGGCAGGGCCCCCGCUCCCC
Plate 10	G04	C-301270-01	hsa-miR-941	MI0005763	CACCCGGCUGUGUGCACAUGUGC
Plate 10	G05	C-301271-01	hsa-miR-941	MI0005764	CACCCGGCUGUGUGCACAUGUGC
Plate 10	G06	C-301272-01	hsa-miR-941	MI0005765	CACCCGGCUGUGUGCACAUGUGC
Plate 10	G07	C-301273-01	hsa-miR-941	MI0005766	CACCCGGCUGUGUGCACAUGUGC
Plate 10	G08	C-301274-01	hsa-miR-942	MI0005767	UCUUCUCUGUUUUGGCCAUGUG
Plate 10	G09	C-301275-01	hsa-miR-943	MI0005768	CUGACUGUUGCCGUCCUCCAG
Plate 10	G10	C-301276-01	hsa-miR-944	MI0005769	AAAUUAUUGUACAUCGGAUGAG
Plate 10	G11	C-301277-01	hsa-miR-1224-5p	MI0003764	GUGAGGACUCGGGAGGUGG
Plate 10	H02	C-301278-01	hsa-miR-1224-3p	MI0003764	CCCCACCUCCUCUCUCCUCAG
Plate 10	H03	C-301279-01	hsa-miR-877	MI0005561	GUAGAGGAGAUGGCGCAGGG
Plate 10	H04	C-301280-01	hsa-miR-877*	MI0005561	UCCUCUUCUCCCUCCUCCCAG
Plate 10	H05	C-301281-01	hsa-miR-1225-5p	MI0006311	GUGGGUACGGCCCAGUGGGGGG
Plate 10	H06	C-301282-01	hsa-miR-1225-3p	MI0006311	UGAGCCCCUGUGCCGCCCCCAG
Plate 10	H07	C-301283-01	hsa-miR-1226*	MI0006313	GUGAGGGCAUGCAGGCCUGGAUGGGG
Plate 10	H08	C-301284-01	hsa-miR-1226	MI0006313	UCACCAGCCCUGUGUUCCCUAG
Plate 10	H09	C-301285-01	hsa-miR-1227	MI0006316	CGUGCCACCCUUUUCCCCAG
Plate 10	H10	C-301286-01	hsa-miR-1228	MI0006318	UCACACCUGCCUCGCCCCCC
Plate 10	H11	C-301287-01	hsa-miR-1228*	MI0006318	GUGGGCGGGGGCAGGUGUGUG
Plate 11	A02	C-301288-01	hsa-miR-1229	MI0006319	CUCUCACCACUGCCCUCCCACAG
Plate 11	A03	C-301289-01	hsa-miR-1231	MI0006321	GUGUCUGGGCGGACAGCUGC

Plate 11	A04	C-301290-01	hsa-miR-1233	MI0006323	UGAGCCCUGUCCUCCCGCAG
Plate 11	A05	C-301291-01	hsa-miR-1234	MI0006324	UCGGCCUGACCACCCACCCCAC
Plate 11	A06	C-301292-01	hsa-miR-1236	MI0006326	CCUCUUCCCCUUGUCUCUCCAG
Plate 11	A07	C-301293-01	hsa-miR-1237	MI0006327	UCCUUCUGCUCCGUCCCCCAG
Plate 11	A08	C-301294-01	hsa-miR-1238	MI0006328	CUUCCUCGUCUGUCUGCCCC
Plate 11	A09	C-301295-01	hsa-miR-513b	MI0006648	UUCACAAGGAGGUGUCAUUUAU
Plate 11	A10	C-301296-01	hsa-miR-513c	MI0006649	UUCUCAAGGAGGUGUCGUUUAU
Plate 11	A11	C-300734-05	hsa-miR-451	MI0001729	AAACCGUUACCAUUACUGAGUU
