# Supplementary material for: A Network-Based Method to Assess the Statistical Significance of Mild Co-Regulation Effects
Source: PLoS One. 2013 Sep 9;8(9):e73413. doi: 10.1371/journal.pone.0073413 (PMC3767771; doi:10.1371/journal.pone.0073413)
Supplement: Table S3 — Groups of miRNAs defined by the SICORE algorithm and their corresponding sequences. (PDF) [file pone.0073413.s005.pdf]

τ_B=0.01 and τ_P = 0.0509

miRNA	SICORE group	mature sequence	seed sequence
hsa-miR-99a	1	AACCCGUAGAUCCGAUCUUGUG	ACCCGU
hsa-miR-767-3p	1	UCUGCUCAUACCCCAUGGUUUCU	CUGCUC
hsa-miR-496	1	UGAGUAUUACAUGGCCAAUCUC	GAGUAU
hsa-miR-421	1	AUCAACAGACAUUAAUUGGGCGC	UCAACA
hsa-miR-202	1	AGAGGUAUAGGGCAUGGGAA	GAGGUA
hsa-miR-127-5p	1	CUGAAGCUCAGAGGGCUCUGAU	UGAAGC
hsa-miR-100	1	AACCCGUAGAUCCGAACUUGUG	ACCCGU
hsa-miR-940	4	AAGGCAGGGCCCCCGCUCCCC	AGGCAG
hsa-miR-527	4	CUGCAAAGGGAAGCCCUUUC	UGCAAA
hsa-miR-449	4	UGGCAGUGUAUUGUUAGCUGGU	GGCAGU
hsa-miR-34b*	4	UAGGCAGUGUCAUUAGCUGAUUG	AGGCAG
hsa-miR-34a	4	UGGCAGUGUCUUAGCUGGUUGU	GGCAGU
hsa-miR-938	5	UGCCCUUAAAGGUGAACCCAGU	GCCCUU
hsa-miR-610	5	UGAGCUAAAUGUGUGCUGGGA	GAGCUA
hsa-miR-521	5	AACGCACUUCCCUUUAGAGUGU	ACGCAC
hsa-miR-519a	5	AAAGUGCAUCCUUUUAGAGUGU	AAGUGC
hsa-miR-31	5	AGGCAAGAUGCUGGCAUAGCU	GGCAAG
hsa-miR-196a*	5	CGGCAACAAGAAACUGCCUGAG	GGCAAC
hsa-miR-18a	5	UAAGGUGCAUCUAGUGCAGAUAG	AAGGUG
hsa-miR-936	6	ACAGUAGAGGGAGGAAUCGCAG	CAGUAG
hsa-miR-520a	6	AAAGUGCUUCCCUUUGGACUGU	AAGUGC
hsa-miR-220c	6	ACACAGGGCUGUUGUGAAGACU	CACAGG
hsa-miR-144	6	UACAGUAUAGAUGAUGUACU	ACAGUA
hsa-miR-924	7	AGAGUCUUGUGAUGUCUUGC	GAGUCU
hsa-miR-627	7	GUGAGUCUCUAAGAAAAGAGGA	UGAGUC
hsa-miR-499-3p	7	AACAUCACAGCAAGUCUGUGCU	ACAUCA
hsa-miR-429	7	UAAUACUGUCUGGUAAAACCGU	AAUACU
hsa-miR-331	7	GCCCCUGGGCCUAUCCUAGAA	CCCCUG
hsa-miR-200c	7	UAAUACUGCCGGGUAAUGAUGGA	AAUACU
hsa-miR-200b	7	UAAUACUGCCUGGUAAUGAUGA	AAUACU
hsa-miR-922	8	GCAGCAGAGAAUAGGACUACGUC	CAGCAG
hsa-miR-632	8	GUGUCUGCUUCCUGUGGGA	UGUCUG
hsa-miR-631	8	AGACCUGGCCCAGACCUCAGC	GACCUG
hsa-miR-453	8	AGGUUGUCCGUGGUGAGUUCGCA	GGUUGU
hsa-miR-452	8	AACUGUUUGCAGAGGAAACUGA	ACUGUU
hsa-miR-214	8	ACAGCAGGCACAGACAGGCAGU	CAGCAG
hsa-miR-19b-1*	8	AGUUUUGCAGGUUUGCAUCCAGC	GUUUUG
hsa-miR-1231	8	GUGUCUGGGCGGACAGCUGC	UGUCUG
hsa-let-7f-1*	8	CUAUACAAUCUAUUGCCUUCCC	UAUACA
hsa-let-7c*	8	UAGAGUUACACCCUGGGAGUUA	AGAGUU
hsa-let-7b*	8	CUAUACAACCUACUGCCUUCCC	UAUACA
hsa-miR-892a	10	CACUGUGUCCUUUCUGCGUAG	ACUGUG
hsa-miR-28-3p	10	CACUAGAUUGUGAGCUCCUGGA	ACUAGA
hsa-miR-148a*	10	AAAGUUCUGAGACACUCCGACU	AAGUUC
hsa-miR-106b*	10	CCGCACUGUGGGUACUUGCUGC	CGCACU
hsa-miR-891b	11	UGCAACUUACCUGAGUCAUUGA	GCAACU
hsa-miR-548d	11	CAAAAACCACAGUUUCUUUUGC	AAAAAC
hsa-miR-18b	11	UAAGGUGCAUCUAGUGCAGUUAG	AAGGUG
hsa-miR-101	11	UACAGUACUGUGAUAACUGAA	ACAGUA
hsa-let-7i*	11	CUGCGCAAGCUACUGCCUUGCU	UGCGCA
hsa-miR-885-3p	12	AGGCAGCGGGGUGUAGUGGAUA	GGCAGC
hsa-miR-624*	12	UAGUACCAGUACCUUGUGUUCA	AGUACC
hsa-miR-491	12	AGUGGGGAACCCUUCCAUGAGG	GUGGGG
hsa-miR-17-3p	12	ACUGCAGUGAAGGCACUUGUAG	CUGCAG
hsa-miR-151	12	CUAGACUGAAGCUCCUUGAGG	UAGACU

hsa-miR-137	12	UUAUUGCUUAAGAAUACGCGUAG	UAUUGC
hsa-miR-126	12	UCGUACCGUGAGUAAUAAUGCG	CGUACC
hsa-miR-874	13	CUGCCCUGGCCCGAGGGACCGA	UGCCCU
hsa-miR-634	13	AACCAGCACCCCAACUUUGGAC	ACCAGC
hsa-miR-612	13	GCUGGGCAGGGCUUCUGAGCUCCUU	CUGGGC
hsa-miR-593*	13	AGGCACCAGCCAGGCAUUGCUCAGC	GGCACC
hsa-miR-566	13	GGGCGCCUGUGAUCCCAAC	GGCGCC
hsa-miR-376a*	13	GUAGAUUCUCCUUCUAUGAGUA	UAGAUU
hsa-miR-323	13	CACAUUACACGGUCGACCUCU	ACAUUA
hsa-miR-29c	13	UAGCACCAUUUGAAAUCGGUUA	AGCACC
hsa-miR-29a	13	UAGCACCAUCUGAAAUCGGUUA	AGCACC
hsa-miR-298	13	AGCAGAAGCAGGGAGGUUCUCCCA	GCAGAA
hsa-miR-15a*	13	CAGGCCAUAUUGUGCUGCCUCA	AGGCCA
hsa-miR-671-3p	16	UCCGGUUCUCAGGGCUCCACC	CCGGUU
hsa-miR-636	16	UGUGCUUGCUCGUCCCGCCCGCA	GUGCUU
hsa-miR-185	16	UGGAGAGAAAGGCAGUUCCUGA	GGAGAG
hsa-miR-671	17	AGGAAGCCCUGGAGGGGCUGGAG	GGAAGC
hsa-miR-644	17	AGUGUGGCUUUCUUAGAGC	GUGUGG
hsa-miR-625	17	AGGGGGAAAGUUCUAUAGUCC	GGGGGA
hsa-miR-490-5p	17	CCAUGGAUCUCCAGGUGGGU	CAUGGA
hsa-miR-187	17	UCGUGUCUUGUGUUGCAGCCGG	CGUGUC
hsa-miR-184	17	UGGACGGAGAACUGAUAAGGGU	GGACGG
hsa-miR-151-5p	17	UCGAGGAGCUCACAGUCUAGU	CGAGGA
hsa-miR-661	18	UGCCUGGGUCUCUGGCCUGCGCGU	GCCUGG
hsa-miR-1	18	UGGAAUGUAAAGAAGUAUGUAU	GGAAUG
hsa-miR-642	20	GUCCCUCUCCAAAUGUGUCUUG	UCCCUC
hsa-miR-582-3p	20	UAACUGGUUGAACAACUGAACC	AACUGG
hsa-miR-556-3p	20	AUAUUACCAUUAGCUCAUCUUU	UAUUAC
hsa-miR-519e	20	AAGUGCCUCCUUUUAGAGUGUU	AGUGCC
hsa-miR-515-3p	20	GAGUGCCUUCUUUUGGAGCGUU	AGUGCC
hsa-miR-511	20	GUGUCUUUUGCUCUGCAGUCA	UGUCUU
hsa-miR-30e-3p	20	CUUUCAGUCGGAUGUUUACAGC	UUUCAG
hsa-miR-1227	20	CGUGCCACCCUUUUCCCCAG	GUGCCA
hsa-miR-637	21	ACUGGGGGCUUUCGGGCUCUGCGU	CUGGGG
hsa-miR-517*	21	CCUCUAGAUGGAAGCACUGUCU	CUCUAG
hsa-miR-21	21	UAGCUUAUCAGACUGAUGUUGA	AGCUUA
hsa-miR-15b	21	UAGCAGCACAUCAUGGUUUACA	AGCAGC
hsa-miR-613	25	AGGAAUGUUCCUUCUUUGCC	GGAAUG
hsa-miR-544	25	AUUCUGCAUUUUUAGCAAGUUC	UUCUGC
hsa-miR-524*	25	CUACAAAGGGAAGCACUUUCUC	UACAAA
hsa-miR-520d*	25	CUACAAAGGGAAGCCCUUUC	UACAAA
hsa-miR-493	25	UUGUACAUGGUAGGCUUUCAUU	UGUACA
hsa-miR-206	25	UGGAAUGUAAGGAAGUGUGUGG	GGAAUG
hsa-miR-155	25	UUAAUGCUAAUCGUGAUAGGGGU	UAAUGC
hsa-miR-146b	25	UGAGAACUGAAUUCCAUAGGCU	GAGAAC
hsa-miR-146a	25	UGAGAACUGAAUUCCAUGGGUU	GAGAAC
hsa-miR-140	25	CAGUGGUUUUACCCUAUGGUAG	AGUGGU
hsa-let-7g*	25	CUGUACAGGCCACUGCCUUGC	UGUACA
hsa-miR-582	29	UUACAGUUGUUCAACCAGUUACU	UACAGU
hsa-miR-181a-2*	29	ACCACUGACCGUUGACUGUACC	CCACUG
hsa-miR-578	30	CUUCUUGUGCUCUAGGAUUGU	UUCUUG
hsa-miR-297	30	AUGUAUGUGUGCAUGUGCAUG	UGUAUG
hsa-miR-223*	30	CGUGUAUUUGACAAGCUGAGUU	GUGUAU
hsa-miR-194*	30	CCAGUGGGGCUGCUGUUAUCUG	CAGUGG
hsa-miR-132*	30	ACCGUGGCUUUCGAUUGUUACU	CCGUGG
hsa-miR-573	31	CUGAAGUGAUGUGUAACUGAUCAG	UGAAGU
hsa-miR-450	31	UUUUGCGAUGUGUUCCUAAUAU	UUUGCG
hsa-miR-380-5p	31	UGGUUGACCAUAGAACAUGCGC	GGUUGA
hsa-miR-558	32	UGAGCUGCUGUACCAAAAU	GAGCUG
hsa-miR-299-5p	32	UGGUUUACCGUCCCACAUACAU	GGUUUA

hsa-miR-552	33	AACAGGUGACUGGUUAGACAA	ACAGGU
hsa-miR-542-3p	33	UGUGACAGAUUGAUAACUGAAA	GUGACA
hsa-miR-512-3p	33	AAGUGCUGUCAUAGCUGAGGUC	AGUGCU
hsa-miR-497	33	CAGCAGCACACUGUGGUUUGU	AGCAGC
hsa-miR-191	33	CAACGGAAUCCCAAAAGCAGCUG	AACGGA
hsa-miR-138	33	AGCUGGUGUUGUGAAUCAGGCCG	GCUGGU
hsa-miR-550	34	UGUCUUACUCCCUCAGGCACAU	GUCUUA
hsa-miR-541	34	UGGUGGGCACAGAAUCUGGACU	GGUGGG
hsa-miR-526b	34	CUCUUGAGGGAAGCACUUUCUGU	UCUUGA
hsa-miR-323-5p	34	AGGUGGUCCGUGGCGCGUUCGC	GGUGGU
hsa-miR-525	36	CUCCAGAGGGAUGCACUUUCU	UCCAGA
hsa-miR-193b	36	AACUGGCCCUCAAAGUCCCGCU	ACUGGC
hsa-miR-520a*	39	CUCCAGAGGGAAGUACUUUCU	UCCAGA
hsa-miR-181c	39	AACAUUCAACCUGUCGGUGAGU	ACAUUC
hsa-miR-519b-5p	41	CUCUAGAGGGAAGCGCUUUCUG	UCUAGA
hsa-let-7f	41	UGAGGUAGUAGAUUGUAUAGUU	GAGGUA
hsa-miR-513	43	UUCACAGGGAGGUGUCAU	UCACAG
hsa-miR-489	43	GUGACAUCACAUAUACGGCAGC	UGACAU
hsa-miR-450b-3p	43	UUGGGAUCAUUUUGCAUCCAUA	UGGGAU
hsa-miR-346	43	UGUCUGCCCGCAUGCCUGCCUCU	GUCUGC
hsa-miR-18b*	43	UGCCCUAAAUGCCCCUUCUGGC	GCCCUA
hsa-let-7e*	43	CUAUACGGCCUCCUAGCUUUCC	UAUACG
hsa-miR-509-5p	44	UACUGCAGACAGUGGCAAUCA	ACUGCA
hsa-miR-328	44	CUGGCCCUCUCUGCCCUUCCGU	UGGCCC
hsa-miR-136*	44	CAUCAUCGUCUCAAAUGAGUCU	AUCAUC
hsa-miR-135b*	44	AUGUAGGGCUAAAAGCCAUGGG	UGUAGG
hsa-miR-130a*	44	UUCACAUUGUGCUACUGUCUGC	UCACAU
hsa-miR-509-3p	45	UGAUUGGUACGUCUGUGGGUAG	GAUUGG
hsa-miR-509	45	UGAUUGGUACGUCUGUGGGUAG	GAUUGG
hsa-miR-26a	45	UUCAAGUAAUCCAGGAUAGGCU	UCAAGU
hsa-miR-502	46	AUCCUUGCUAUCUGGGUGCUA	UCCUUG
hsa-miR-141*	46	CAUCUUCCAGUACAGUGUUGGA	AUCUUC
hsa-miR-449b	48	AGGCAGUGUAUUGUUAGCUGGC	GGCAGU
hsa-miR-30b*	48	CUGGGAGGUGGAUGUUUACUUC	UGGGAG
hsa-miR-326	54	CCUCUGGGCCCUUCCUCCAG	CUCUGG
hsa-miR-218-1*	54	AUGGUUCCGUCAAGCACCAUGG	UGGUUC

**Number of miRNAs**	**Number of groups**
151	31

τ_B=0.05 and τ_P = 0.0459

miRNA	SICORE group	mature sequence	seed sequence
hsa-miR-99b	1	CACCCGUAGAACCGACCUUGCG	ACCCGU
hsa-miR-99a	1	AACCCGUAGAUCCGAUCUUGUG	ACCCGU
hsa-miR-767-3p	1	UCUGCUCAUACCCCAUGGUUUCU	CUGCUC
hsa-miR-616	1	AGUCAUUGGAGGGUUUGAGCAG	GUCAUU
hsa-miR-517c	1	AUCGUGCAUCCUUUUAGAGUGU	UCGUGC
hsa-miR-517b	1	UCGUGCAUCCCUUUAGAGUGUU	CGUGCA
hsa-miR-517a	1	AUCGUGCAUCCCUUUAGAGUGU	UCGUGC
hsa-miR-515-5p	1	UUCUCCAAAAGAAAGCACUUUCUG	UCUCCA
hsa-miR-34c-3p	1	AAUCACUAACCACACGGCCAGG	AUCACU
hsa-miR-150*	1	CUGGUACAGGCCUGGGGGACAG	UGGUAC
hsa-miR-127	1	UCGGAUCCGUCUGAGCUUGGCU	CGGAUC
hsa-miR-100	1	AACCCGUAGAUCCGAACUUGUG	ACCCGU
hsa-miR-99a*	2	CAAGCUCGCUUCUAUGGGUCUG	AAGCUC
hsa-miR-922	2	GCAGCAGAGAAUAGGACUACGUC	CAGCAG
hsa-miR-802	2	CAGUAACAAAGAUUCAUCCUUGU	AGUAAC
hsa-miR-632	2	GUGUCUGCUUCCUGUGGGA	UGUCUG
hsa-miR-583	2	CAAAGAGGAAGGUCCCAUUAC	AAAGAG
hsa-miR-507	2	UUUUGCACCUUUUGGAGUGAA	UUUGCA
hsa-miR-452	2	AACUGUUUGCAGAGGAAACUGA	ACUGUU
hsa-miR-24	2	UGGCUCAGUUCAGCAGGAACAG	GGCUCA
hsa-miR-215	2	AUGACCUAUGAAUUGACAGAC	UGACCU
hsa-miR-214	2	ACAGCAGGCACAGACAGGCAGU	CAGCAG
hsa-miR-212	2	UAACAGUCUCCAGUCACGGCC	AACAGU
hsa-miR-19b-1*	2	AGUUUUGCAGGUUUGCAUCCAGC	GUUUUG
hsa-miR-1231	2	GUGUCUGGGCGGACAGCUGC	UGUCUG
hsa-miR-96	3	UUUGGCACUAGCACAUUUUUGCU	UUGGCA
hsa-miR-202	3	AGAGGUAUAGGGCAUGGGAA	GAGGUA
hsa-miR-940	5	AAGGCAGGGCCCCCGCUCCCC	AGGCAG
hsa-miR-892b	5	CACUGGCUCCUUUCUGGGUAGA	ACUGGC
hsa-miR-885-3p	5	AGGCAGCGGGGUGUAGUGGAUA	GGCAGC
hsa-miR-877*	5	UCCUCUUCUCCCUCCUCCCAG	CCUCUU
hsa-miR-769-5p	5	UGAGACCUCUGGGUUCUGAGCU	GAGACC
hsa-miR-766	5	ACUCCAGCCCCACAGCCUCAGC	CUCCAG
hsa-miR-644	5	AGUGUGGCUUUCUUAGAGC	GUGUGG
hsa-miR-642	5	GUCCCUCUCCAAAUGUGUCUUG	UCCCUC
hsa-miR-640	5	AUGAUCCAGGAACCUGCCUCU	UGAUCC
hsa-miR-637	5	ACUGGGGGCUUUCGGGCUCUGCGU	CUGGGG
hsa-miR-634	5	AACCAGCACCCCAACUUUGGAC	ACCAGC
hsa-miR-631	5	AGACCUGGCCCAGACCUCAGC	GACCUG
hsa-miR-629*	5	GUUCUCCCAACGUAAGCCCAGC	UUCUCC
hsa-miR-624*	5	UAGUACCAGUACCUUGUGUUCA	AGUACC
hsa-miR-624	5	CACAAGGUAUUGGUAUUACCU	ACAAGG
hsa-miR-608	5	AGGGGUGGUGUUGGGACAGCUCCGU	GGGGUG
hsa-miR-582	5	UUACAGUUGUUCAACCAGUUACU	UACAGU
hsa-miR-569	5	AGUUAAUGAAUCCUGGAAAGU	GUUAAU
hsa-miR-566	5	GGGCGCCUGUGAUCCCAAC	GGCGCC
hsa-miR-562	5	AAAGUAGCUGUACCAUUUGC	AAGUAG
hsa-miR-558	5	UGAGCUGCUGUACCAAAAU	GAGCUG
hsa-miR-555	5	AGGGUAAGCUGAACCUCUGAU	GGGUAA
hsa-miR-548d	5	CAAAAACCACAGUUUCUUUUGC	AAAAAC
hsa-miR-542-3p	5	UGUGACAGAUUGAUAACUGAAA	GUGACA
hsa-miR-541	5	UGGUGGGCACAGAAUCUGGACU	GGUGGG
hsa-miR-532	5	CAUGCCUUGAGUGUAGGACCGU	AUGCCU

hsa-miR-525	5	CUCCAGAGGGAUGCACUUUCU	UCCAGA
hsa-miR-524*	5	CUACAAAGGGAAGCACUUUCUC	UACAAA
hsa-miR-520e	5	AAAGUGCUUCCUUUUUGAGGG	AAGUGC
hsa-miR-520d	5	AAAGUGCUUCUCUUUGGUGGGU	AAGUGC
hsa-miR-520c	5	AAAGUGCUUCCUUUUAGAGGGU	AAGUGC
hsa-miR-520b	5	AAAGUGCUUCCUUUUAGAGGG	AAGUGC
hsa-miR-520a*	5	CUCCAGAGGGAAGUACUUUCU	UCCAGA
hsa-miR-520a	5	AAAGUGCUUCCCUUUGGACUGU	AAGUGC
hsa-miR-519b-5p	5	CUCUAGAGGGAAGCGCUUUCUG	UCUAGA
hsa-miR-519b	5	AAAGUGCAUCCUUUUAGAGGUU	AAGUGC
hsa-miR-519a*	5	CUCUAGAGGGAAGCGCUUUCUG	UCUAGA
hsa-miR-519a	5	AAAGUGCAUCCUUUUAGAGUGU	AAGUGC
hsa-miR-518e*	5	CUCUAGAGGGAAGCGCUUUCUG	UCUAGA
hsa-miR-518a-5p	5	CUGCAAAGGGAAGCCCUUUC	UGCAAA
hsa-miR-511	5	GUGUCUUUUGCUCUGCAGUCA	UGUCUU
hsa-miR-509-3p	5	UGAUUGGUACGUCUGUGGGUAG	GAUUGG
hsa-miR-509	5	UGAUUGGUACGUCUGUGGGUAG	GAUUGG
hsa-miR-499-3p	5	AACAUCACAGCAAGUCUGUGCU	ACAUCA
hsa-miR-486	5	UCCUGUACUGAGCUGCCCCGAG	CCUGUA
hsa-miR-449b	5	AGGCAGUGUAUUGUUAGCUGGC	GGCAGU
hsa-miR-449	5	UGGCAGUGUAUUGUUAGCUGGU	GGCAGU
hsa-miR-433	5	AUCAUGAUGGGCUCCUCGGUGU	UCAUGA
hsa-miR-372	5	AAAGUGCUGCGACAUUUGAGCGU	AAGUGC
hsa-miR-34b*	5	UAGGCAGUGUCAUUAGCUGAUUG	AGGCAG
hsa-miR-34a*	5	CAAUCAGCAAGUAUACUGCCCU	AAUCAG
hsa-miR-34a	5	UGGCAGUGUCUUAGCUGGUUGU	GGCAGU
hsa-miR-346	5	UGUCUGCCCGCAUGCCUGCCUCU	GUCUGC
hsa-miR-342-5p	5	AGGGGUGCUAUCUGUGAUUGA	GGGGUG
hsa-miR-338	5	UCCAGCAUCAGUGAUUUUGUUG	CCAGCA
hsa-miR-329	5	AACACACCUGGUUAACCUCUUU	ACACAC
hsa-miR-326	5	CCUCUGGGCCCUUCCUCCAG	CUCUGG
hsa-miR-323-5p	5	AGGUGGUCCGUGGCGCGUUCGC	GGUGGU
hsa-miR-323	5	CACAUUACACGGUCGACCUCU	ACAUUA
hsa-miR-31	5	AGGCAAGAUGCUGGCAUAGCU	GGCAAG
hsa-miR-30b*	5	CUGGGAGGUGGAUGUUUACUUC	UGGGAG
hsa-miR-302a	5	UAAGUGCUUCCAUGUUUUGGUGA	AAGUGC
hsa-miR-299-5p	5	UGGUUUACCGUCCCACAUACAU	GGUUUA
hsa-miR-26a	5	UUCAAGUAAUCCAGGAUAGGCU	UCAAGU
hsa-miR-218-1*	5	AUGGUUCCGUCAAGCACCAUGG	UGGUUC
hsa-miR-208b	5	AUAAGACGAACAAAAGGUUUGU	UAAGAC
hsa-miR-199b-3p	5	ACAGUAGUCUGCACAUUGGUUA	CAGUAG
hsa-miR-193b	5	AACUGGCCCUCAAAGUCCCGCU	ACUGGC
hsa-miR-193a	5	AACUGGCCUACAAAGUCCCAGU	ACUGGC
hsa-miR-18b	5	UAAGGUGCAUCUAGUGCAGUUAG	AAGGUG
hsa-miR-181c	5	AACAUUCAACCUGUCGGUGAGU	ACAUUC
hsa-miR-17-3p	5	ACUGCAGUGAAGGCACUUGUAG	CUGCAG
hsa-miR-16	5	UAGCAGCACGUAAAUAUUGGCG	AGCAGC
hsa-miR-155	5	UUAAUGCUAAUCGUGAUAGGGGU	UAAUGC
hsa-miR-151	5	CUAGACUGAAGCUCCUUGAGG	UAGACU
hsa-miR-148a	5	UCAGUGCACUACAGAACUUUGU	CAGUGC
hsa-miR-147	5	GUGUGUGGAAAUGCUUCUGC	UGUGUG
hsa-miR-145*	5	GGAUUCCUGGAAAUACUGUUCU	GAUUCC
hsa-miR-144*	5	GGAUAUCAUCAUAUACUGUAAG	GAUAUC
hsa-miR-138-2*	5	GCUAUUUCACGACACCAGGGUU	CUAUUU
hsa-miR-137	5	UUAUUGCUUAAGAAUACGCGUAG	UAUUGC
hsa-miR-132	5	UAACAGUCUACAGCCAUGGUCG	AACAGU

hsa-miR-126	5	UCGUACCGUGAGUAAUAAUGCG	CGUACC
hsa-miR-105*	5	ACGGAUGUUUGAGCAUGUGCUA	CGGAUG
hsa-let-7i*	5	CUGCGCAAGCUACUGCCUUGCU	UGCGCA
hsa-let-7g*	5	CUGUACAGGCCACUGCCUUGC	UGUACA
hsa-let-7f-2*	5	CUAUACAGUCUACUGUCUUUCC	UAUACA
hsa-let-7f-1*	5	CUAUACAAUCUAUUGCCUUCCC	UAUACA
hsa-let-7c*	5	UAGAGUUACACCCUGGGAGUUA	AGAGUU
hsa-let-7f	5	UGAGGUAGUAGAUUGUAUAGUU	GAGGUA
hsa-miR-938	6	UGCCCUUAAAGGUGAACCCAGU	GCCCUU
hsa-miR-765	6	UGGAGGAGAAGGAAGGUGAUG	GGAGGA
hsa-miR-610	6	UGAGCUAAAUGUGUGCUGGGA	GAGCUA
hsa-miR-521	6	AACGCACUUCCCUUUAGAGUGU	ACGCAC
hsa-miR-455	6	UAUGUGCCUUUGGACUACAUCG	AUGUGC
hsa-miR-340*	6	UCCGUCUCAGUUACUUUAUAGC	CCGUCU
hsa-miR-196a*	6	CGGCAACAAGAAACUGCCUGAG	GGCAAC
hsa-miR-18a	6	UAAGGUGCAUCUAGUGCAGAUAG	AAGGUG
hsa-miR-936	7	ACAGUAGAGGGAGGAAUCGCAG	CAGUAG
hsa-miR-935	7	CCAGUUACCGCUUCCGCUACCGC	CAGUUA
hsa-miR-876-5p	7	UGGAUUUCUUUGUGAAUCACCA	GGAUUU
hsa-miR-768-3p	7	UCACAAUGCUGACACUCAAACUGCUGAC	CACAAU
hsa-miR-621	7	GGCUAGCAACAGCGCUUACCU	GCUAGC
hsa-miR-584	7	UUAUGGUUUGCCUGGGACUGAG	UAUGGU
hsa-miR-520g	7	ACAAAGUGCUUCCCUUUAGAGUGU	CAAAGU
hsa-miR-518c*	7	UCUCUGGAGGGAAGCACUUUCUG	CUCUGG
hsa-miR-330-5p	7	UCUCUGGGCCUGUGUCUUAGGC	CUCUGG
hsa-miR-1226	7	UCACCAGCCCUGUGUUCCCUAG	CACCAG
hsa-miR-106b	7	UAAAGUGCUGACAGUGCAGAU	AAAGUG
hsa-miR-93	8	CAAAGUGCUGUUCGUGCAGGUAG	AAAGUG
hsa-miR-888	8	UACUCAAAAAGCUGUCAGUCA	ACUCAA
hsa-miR-491-3p	8	CUUAUGCAAGAUUCCCUUCUAC	UUAUGC
hsa-miR-490	8	CAACCUGGAGGACUCCAUGCUG	AACCUG
hsa-miR-219-1-3p	8	AGAGUUGAGUCUGGACGUCCCG	GAGUUG
hsa-miR-155*	8	CUCCUACAUAUUAGCAUUAACA	UCCUAC
hsa-miR-1228	8	UCACACCUGCCUCGCCCCCC	CACACC
hsa-miR-92b	9	UAUUGCACUCGUCCCGGCCUCC	AUUGCA
hsa-miR-575	9	GAGCCAGUUGGACAGGAGC	AGCCAG
hsa-miR-545	9	UCAGCAAACAUUUAUUGUGUGC	CAGCAA
hsa-miR-520h	9	ACAAAGUGCUUCCCUUUAGAGU	CAAAGU
hsa-miR-520c-5p	9	CUCUAGAGGGAAGCACUUUCUG	UCUAGA
hsa-miR-924	10	AGAGUCUUGUGAUGUCUUGC	GAGUCU
hsa-miR-760	10	CGGCUCUGGGUCUGUGGGGA	GGCUCU
hsa-miR-627	10	GUGAGUCUCUAAGAAAAGAGGA	UGAGUC
hsa-miR-429	10	UAAUACUGUCUGGUAAAACCGU	AAUACU
hsa-miR-378	10	CUCCUGACUCCAGGUCCUGUGU	UCCUGA
hsa-miR-33b*	10	CAGUGCCUCGGCAGUGCAGCCC	AGUGCC
hsa-miR-331	10	GCCCCUGGGCCUAUCCUAGAA	CCCCUG
hsa-miR-211	10	UUCCCUUUGUCAUCCUUCGCCU	UCCCUU
hsa-miR-921	11	CUAGUGAGGGACAGAACCAGGAUUC	UAGUGA
hsa-miR-554	11	GCUAGUCCUGACUCAGCCAGU	CUAGUC
hsa-miR-376a	11	AUCAUAGAGGAAAAUCCACGU	UCAUAG
hsa-miR-369-5p	11	AGAUCGACCGUGUUAUAUUCGC	GAUCGA
hsa-miR-103	11	AGCAGCAUUGUACAGGGCUAUGA	GCAGCA
hsa-miR-9	12	UCUUUGGUUAUCUAGCUGUAUGA	CUUUGG
hsa-miR-502	12	AUCCUUGCUAUCUGGGUGCUA	UCCUUG
hsa-miR-141*	12	CAUCUUCCAGUACAGUGUUGGA	AUCUUC
hsa-miR-133b	12	UUUGGUCCCCUUCAACCAGCUA	UUGGUC

hsa-miR-892a	13	CACUGUGUCCUUUCUGCGUAG	ACUGUG
hsa-miR-28-3p	13	CACUAGAUUGUGAGCUCCUGGA	ACUAGA
hsa-miR-199a	13	CCCAGUGUUCAGACUACCUGUUC	CCAGUG
hsa-miR-181a	13	AACAUUCAACGCUGUCGGUGAGU	ACAUUC
hsa-miR-148a*	13	AAAGUUCUGAGACACUCCGACU	AAGUUC
hsa-miR-106b*	13	CCGCACUGUGGGUACUUGCUGC	CGCACU
hsa-let-7a*	13	CUAUACAAUCUACUGUCUUUC	UAUACA
hsa-miR-889	15	UUAAUAUCGGACAACCAUUGU	UAAUAU
hsa-miR-502-3p	15	AAUGCACCUGGGCAAGGAUUCA	AUGCAC
hsa-miR-487	15	AAUCAUACAGGGACAUCCAGUU	AUCAUA
hsa-miR-32	15	UAUUGCACAUUACUAAGUUGCA	AUUGCA
hsa-miR-224	15	CAAGUCACUAGUGGUUCCGUU	AAGUCA
hsa-miR-197	15	UUCACCACCUUCUCCACCCAGC	UCACCA
hsa-miR-181a-2*	15	ACCACUGACCGUUGACUGUACC	CCACUG
hsa-miR-888*	16	GACUGACACCUCUUUGGGUGAA	ACUGAC
hsa-miR-551b	16	GCGACCCAUACUUGGUUUCAG	CGACCC
hsa-miR-526b*	16	GAAAGUGCUUCCUUUUAGAGGC	AAAGUG
hsa-miR-526b	16	CUCUUGAGGGAAGCACUUUCUGU	UCUUGA
hsa-miR-886-3p	17	CGCGGGUGCUUACUGACCCUU	GCGGGU
hsa-miR-626	17	AGCUGUCUGAAAAUGUCUU	GCUGUC
hsa-miR-579	17	UUCAUUUGGUAUAAACCGCGAUU	UCAUUU
hsa-miR-574	17	CACGCUCAUGCACACACCCACA	ACGCUC
hsa-miR-493	17	UUGUACAUGGUAGGCUUUCAUU	UGUACA
hsa-miR-206	17	UGGAAUGUAAGGAAGUGUGUGG	GGAAUG
hsa-miR-193a-5p	17	UGGGUCUUUGCGGGCGAGAUGA	GGGUCU
hsa-miR-146b	17	UGAGAACUGAAUUCCAUAGGCU	GAGAAC
hsa-miR-146a	17	UGAGAACUGAAUUCCAUGGGUU	GAGAAC
hsa-miR-140	17	CAGUGGUUUUACCCUAUGGUAG	AGUGGU
hsa-miR-135a	17	UAUGGCUUUUUAUUCCUAUGUGA	AUGGCU
hsa-miR-876-3p	18	UGGUGGUUUACAAAGUAAUUCA	GGUGGU
hsa-miR-619	18	GACCUGGACAUGUUUGUGCCCAGU	ACCUGG
hsa-miR-452*	18	CUCAUCUGCAAAGAAGUAAGUG	UCAUCU
hsa-miR-450b-3p	18	UUGGGAUCAUUUUGCAUCCAUA	UGGGAU
hsa-miR-18b*	18	UGCCCUAAAUGCCCCUUCUGGC	GCCCUA
hsa-miR-128	18	UCACAGUGAACCGGUCUCUUU	CACAGU
hsa-let-7e*	18	CUAUACGGCCUCCUAGCUUUCC	UAUACG
hsa-miR-874	19	CUGCCCUGGCCCGAGGGACCGA	UGCCCU
hsa-miR-665	19	ACCAGGAGGCUGAGGCCCCU	CCAGGA
hsa-miR-648	19	AAGUGUGCAGGGCACUGGU	AGUGUG
hsa-miR-612	19	GCUGGGCAGGGCUUCUGAGCUCCUU	CUGGGC
hsa-miR-532-3p	19	CCUCCCACACCCAAGGCUUGCA	CUCCCA
hsa-miR-516-5p	19	AUCUGGAGGUAAGAAGCACUUU	UCUGGA
hsa-miR-376a*	19	GUAGAUUCUCCUUCUAUGAGUA	UAGAUU
hsa-miR-29c	19	UAGCACCAUUUGAAAUCGGUUA	AGCACC
hsa-miR-29a	19	UAGCACCAUCUGAAAUCGGUUA	AGCACC
hsa-miR-298	19	AGCAGAAGCAGGGAGGUUCUCCCA	GCAGAA
hsa-miR-222	19	AGCUACAUCUGGCUACUGGGU	GCUACA
hsa-miR-221	19	AGCUACAUUGUCUGCUGGGUUUC	GCUACA
hsa-miR-21*	19	CAACACCAGUCGAUGGGCUGU	AACACC
hsa-miR-202*	19	UUCCUAUGCAUAUACUUCUUUG	UCCUAU
hsa-miR-15a*	19	CAGGCCAUAUUGUGCUGCCUCA	AGGCCA
hsa-miR-138-1*	19	GCUACUUCACAACACCAGGGCC	CUACUU
hsa-miR-768-5p	20	GUUGGAGGAUGAAAGUACGGAGUGAU	UUGGAG
hsa-miR-516-3p	20	UGCUUCCUUUCAGAGGGU	GCUUCC
hsa-miR-373	20	GAAGUGCUUCGAUUUUGGGGUGU	AAGUGC

hsa-miR-302d	20	UAAGUGCUUCCAUGUUUGAGUGU	AAGUGC
hsa-miR-101	20	UACAGUACUGUGAUAACUGAA	ACAGUA
hsa-miR-744*	21	CUGUUGCCACUAACCUCAACCU	UGUUGC
hsa-miR-582-3p	21	UAACUGGUUGAACAACUGAACC	AACUGG
hsa-miR-556-3p	21	AUAUUACCAUUAGCUCAUCUUU	UAUUAC
hsa-miR-519e	21	AAGUGCCUCCUUUUAGAGUGUU	AGUGCC
hsa-miR-371	21	AAGUGCCGCCAUCUUUUGAGUGU	AGUGCC
hsa-miR-30e-3p	21	CUUUCAGUCGGAUGUUUACAGC	UUUCAG
hsa-miR-25	21	CAUUGCACUUGUCUCGGUCUGA	AUUGCA
hsa-miR-19b	21	UGUGCAAAUCCAUGCAAAACUGA	GUGCAA
hsa-miR-188	21	CAUCCCUUGCAUGGUGGAGGG	AUCCCU
hsa-miR-1227	21	CGUGCCACCCUUUUCCCCAG	GUGCCA
hsa-miR-744	22	UGCGGGGCUAGGGCUAACAGCA	GCGGGG
hsa-miR-552	22	AACAGGUGACUGGUUAGACAA	ACAGGU
hsa-miR-297	22	AUGUAUGUGUGCAUGUGCAUG	UGUAUG
hsa-miR-671-3p	24	UCCGGUUCUCAGGGCUCCACC	CCGGUU
hsa-miR-636	24	UGUGCUUGCUCGUCCCGCCCGCA	GUGCUU
hsa-miR-495	24	AAACAAACAUGGUGCACUUCUU	AACAAA
hsa-miR-431*	24	CAGGUCGUCUUGCAGGGCUUCU	AGGUCG
hsa-miR-27a*	24	AGGGCUUAGCUGCUUGUGAGCA	GGGCUU
hsa-miR-222*	24	CUCAGUAGCCAGUGUAGAUCCU	UCAGUA
hsa-miR-196b	24	UAGGUAGUUUCCUGUUGUUGGG	AGGUAG
hsa-miR-185	24	UGGAGAGAAAGGCAGUUCCUGA	GGAGAG
hsa-miR-129-3p	24	AAGCCCUUACCCCAAAAAGCAU	AGCCCU
hsa-miR-122a	24	UGGAGUGUGACAAUGGUGUUUG	GGAGUG
hsa-miR-671	25	AGGAAGCCCUGGAGGGGCUGGAG	GGAAGC
hsa-miR-650	25	AGGAGGCAGCGCUCUCAGGAC	GGAGGC
hsa-miR-625	25	AGGGGGAAAGUUCUAUAGUCC	GGGGGA
hsa-miR-564	25	AGGCACGGUGUCAGCAGGC	GGCACG
hsa-miR-485-5p	25	AGAGGCUGGCCGUGAUGAAUUC	GAGGCU
hsa-miR-187	25	UCGUGUCUUGUGUUGCAGCCGG	CGUGUC
hsa-miR-184	25	UGGACGGAGAACUGAUAAGGGU	GGACGG
hsa-miR-151-5p	25	UCGAGGAGCUCACAGUCUAGU	CGAGGA
hsa-miR-661	26	UGCCUGGGUCUCUGGCCUGCGCGU	GCCUGG
hsa-miR-520d*	26	CUACAAAGGGAAGCCCUUUC	UACAAA
hsa-miR-635	28	ACUUGGGCACUGAAACAAUGUCC	CUUGGG
hsa-miR-588	28	UUGGCCACAAUGGGUUAGAAC	UGGCCA
hsa-miR-628-5p	29	AUGCUGACAUAUUUACUAGAGG	UGCUGA
hsa-miR-527	29	CUGCAAAGGGAAGCCCUUUC	UGCAAA
hsa-miR-425	29	AUCGGGAAUGUCGUGUCCGCCC	UCGGGA
hsa-miR-34c	29	AGGCAGUGUAGUUAGCUGAUUGC	GGCAGU
hsa-miR-198	29	GGUCCAGAGGGGAGAUAGGUUC	GUCCAG
hsa-miR-149*	29	AGGGAGGGACGGGGGCUGUGC	GGGAGG
hsa-miR-623	30	AUCCCUUGCAGGGGCUGUUGGGU	UCCCUU
hsa-miR-187*	30	GGCUACAACACAGGACCCGGGC	GCUACA
hsa-miR-605	33	UAAAUCCCAUGGUGCCUUCUCCU	AAAUCC
hsa-miR-601	33	UGGUCUAGGAUUGUUGGAGGAG	GGUCUA
hsa-miR-510	33	UACUCAGGAGAGUGGCAAUCAC	ACUCAG
hsa-miR-509-3-5p	33	UACUGCAGACGUGGCAAUCAUG	ACUGCA
hsa-miR-361-3p	33	UCCCCCAGGUGUGAUUCUGAUUU	CCCCCA
hsa-miR-328	33	CUGGCCCUCUCUGCCCUUCCGU	UGGCCC
hsa-miR-136*	33	CAUCAUCGUCUCAAAUGAGUCU	AUCAUC
hsa-miR-135b*	33	AUGUAGGGCUAAAAGCCAUGGG	UGUAGG
hsa-miR-130a*	33	UUCACAUUGUGCUACUGUCUGC	UCACAU
hsa-miR-593*	35	AGGCACCAGCCAGGCAUUGCUCAGC	GGCACC
hsa-miR-590	35	GAGCUUAUUCAUAAAAGUGCAG	AGCUUA

hsa-miR-592	36	UUGUGUCAAUAUGCGAUGAUGU	UGUGUC
hsa-miR-544	36	AUUCUGCAUUUUUAGCAAGUUC	UUCUGC
hsa-miR-491	36	AGUGGGGAACCCUUCCAUGAGG	GUGGGG
hsa-miR-421	36	AUCAACAGACAUUAAUUGGGCGC	UCAACA
hsa-miR-127-5p	36	CUGAAGCUCAGAGGGCUCUGAU	UGAAGC
hsa-miR-589	37	UGAGAACCACGUCUGCUCUGAG	GAGAAC
hsa-miR-422a	37	ACUGGACUUAGGGUCAGAAGGC	CUGGAC
hsa-miR-223	37	UGUCAGUUUGUCAAAUACCCCA	GUCAGU
hsa-miR-15a	37	UAGCAGCACAUAAUGGUUUGUG	AGCAGC
hsa-miR-581	38	UCUUGUGUUCUCUAGAUCAGU	CUUGUG
hsa-miR-148b*	38	AAGUUCUGUUAUACACUCAGGC	AGUUCU
hsa-miR-567	41	AGUAUGUUCUUCCAGGACAGAAC	GUAUGU
hsa-miR-512-5p	41	CACUCAGCCUUGAGGGCACUUUC	ACUCAG
hsa-miR-422b	41	ACUGGACUUGGAGUCAGAAGG	CUGGAC
hsa-miR-363*	41	CGGGUGGAUCACGAUGCAAUUU	GGGUGG
hsa-miR-302d*	41	ACUUUAACAUGGAGGCACUUGC	CUUUAA
hsa-miR-223*	41	CGUGUAUUUGACAAGCUGAGUU	GUGUAU
hsa-miR-221*	41	ACCUGGCAUACAAUGUAGAUUU	CCUGGC
hsa-miR-200a	41	UAACACUGUCUGGUAACGAUGU	AACACU
hsa-miR-194*	41	CCAGUGGGGCUGCUGUUAUCUG	CAGUGG
hsa-miR-146b-3p	41	UGCCCUGUGGACUCAGUUCUGG	GCCCUG
hsa-miR-132*	41	ACCGUGGCUUUCGAUUGUUACU	CCGUGG
hsa-miR-550	42	UGUCUUACUCCCUCAGGCACAU	GUCUUA
hsa-miR-522	42	AAAAUGGUUCCCUUUAGAGUGU	AAAUGG
hsa-miR-489	42	GUGACAUCACAUAUACGGCAGC	UGACAU
hsa-miR-200c	42	UAAUACUGCCGGGUAAUGAUGGA	AAUACU
hsa-miR-200b	42	UAAUACUGCCUGGUAAUGAUGA	AAUACU
hsa-miR-523*	44	CUCUAGAGGGAAGCGCUUUCUG	UCUAGA
hsa-miR-496	44	UGAGUAUUACAUGGCCAAUCUC	GAGUAU
hsa-miR-490-5p	44	CCAUGGAUCUCCAGGUGGGU	CAUGGA
hsa-miR-520f	45	AAGUGCUUCCUUUUAGAGGGUU	AGUGCU
hsa-miR-515-3p	45	GAGUGCCUUCUUUUGGAGCGUU	AGUGCC
hsa-miR-519c	46	AAAGUGCAUCUUUUUAGAGGAU	AAGUGC
hsa-miR-483-5p	46	AAGACGGGAGGAAAGAAGGGAG	AGACGG
hsa-miR-517*	47	CCUCUAGAUGGAAGCACUGUCU	CUCUAG
hsa-miR-15b	47	UAGCAGCACAUCAUGGUUUACA	AGCAGC
hsa-miR-450	54	UUUUGCGAUGUGUUCCUAAUAU	UUUGCG
hsa-miR-191	54	CAACGGAAUCCCAAAAGCAGCUG	AACGGA
hsa-miR-412	55	ACUUCACCUGGUCCACUAGCCGU	CUUCAC
hsa-miR-380-5p	55	UGGUUGACCAUAGAACAUGCGC	GGUUGA
hsa-miR-365	56	UAAUGCCCCUAAAAAUCCUUAU	AAUGCC
hsa-miR-26a-2*	56	CCUAUUCUUGAUUACUUGUUUC	CUAUUC
hsa-miR-342	57	UCUCACACAGAAAUCGCACCCGU	CUCACA
hsa-miR-192	57	CUGACCUAUGAAUUGACAGCC	UGACCU
hsa-miR-154	69	UAGGUUAUCCGUGUUGCCUUCG	AGGUUA
hsa-miR-147b	69	GUGUGCGGAAAUGCUUCUGCUA	UGUGCG

**Number of miRNAs**	**Number of groups**
322	42

τ_B=0.1 and τ_P = 0.0440

miRNA	SICORE group	mature sequence	seed sequence
hsa-miR-99b*	1	CAAGCUCGUGUCUGUGGGUCCG	AAGCUC
hsa-miR-99a*	1	CAAGCUCGCUUCUAUGGGUCUG	AAGCUC
hsa-miR-802	1	CAGUAACAAAGAUUCAUCCUUGU	AGUAAC
hsa-miR-632	1	GUGUCUGCUUCCUGUGGGA	UGUCUG
hsa-miR-583	1	CAAAGAGGAAGGUCCCAUUAC	AAAGAG
hsa-miR-522*	1	CUCUAGAGGGAAGCGCUUUCUG	UCUAGA
hsa-miR-507	1	UUUUGCACCUUUUGGAGUGAA	UUUGCA
hsa-miR-452	1	AACUGUUUGCAGAGGAAACUGA	ACUGUU
hsa-miR-24	1	UGGCUCAGUUCAGCAGGAACAG	GGCUCA
hsa-miR-1238	1	CUUCCUCGUCUGUCUGCCCC	UUCCUC
hsa-miR-1231	1	GUGUCUGGGCGGACAGCUGC	UGUCUG
hsa-miR-99b	2	CACCCGUAGAACCGACCUUGCG	ACCCGU
hsa-miR-625*	2	GACUAUAGAACUUUCCCCCUCA	ACUAUA
hsa-miR-616	2	AGUCAUUGGAGGGUUUGAGCAG	GUCAUU
hsa-miR-572	2	GUCCGCUCGGCGGUGGCCCA	UCCGCU
hsa-miR-517b	2	UCGUGCAUCCCUUUAGAGUGUU	CGUGCA
hsa-miR-150*	2	CUGGUACAGGCCUGGGGGACAG	UGGUAC
hsa-miR-127	2	UCGGAUCCGUCUGAGCUUGGCU	CGGAUC
hsa-miR-100	2	AACCCGUAGAUCCGAACUUGUG	ACCCGU
hsa-miR-99a	3	AACCCGUAGAUCCGAUCUUGUG	ACCCGU
hsa-miR-767-3p	3	UCUGCUCAUACCCCAUGGUUUCU	CUGCUC
hsa-miR-96	4	UUUGGCACUAGCACAUUUUUGCU	UUGGCA
hsa-miR-942	4	UCUUCUCUGUUUUGGCCAUGUG	CUUCUC
hsa-miR-940	4	AAGGCAGGGCCCCCGCUCCCC	AGGCAG
hsa-miR-938	4	UGCCCUUAAAGGUGAACCCAGU	GCCCUU
hsa-miR-934	4	UGUCUACUACUGGAGACACUGG	GUCUAC
hsa-miR-922	4	GCAGCAGAGAAUAGGACUACGUC	CAGCAG
hsa-miR-921	4	CUAGUGAGGGACAGAACCAGGAUUC	UAGUGA
hsa-miR-892b	4	CACUGGCUCCUUUCUGGGUAGA	ACUGGC
hsa-miR-885-3p	4	AGGCAGCGGGGUGUAGUGGAUA	GGCAGC
hsa-miR-877*	4	UCCUCUUCUCCCUCCUCCCAG	CCUCUU
hsa-miR-769-5p	4	UGAGACCUCUGGGUUCUGAGCU	GAGACC
hsa-miR-768-5p	4	GUUGGAGGAUGAAAGUACGGAGUGAU	UUGGAG
hsa-miR-766	4	ACUCCAGCCCCACAGCCUCAGC	CUCCAG
hsa-miR-744	4	UGCGGGGCUAGGGCUAACAGCA	GCGGGG
hsa-miR-675	4	UGGUGCGGAGAGGGCCCACAGUG	GGUGCG
hsa-miR-671-3p	4	UCCGGUUCUCAGGGCUCCACC	CCGGUU
hsa-miR-665	4	ACCAGGAGGCUGAGGCCCCU	CCAGGA
hsa-miR-661	4	UGCCUGGGUCUCUGGCCUGCGCGU	GCCUGG
hsa-miR-646	4	AAGCAGCUGCCUCUGAGGC	AGCAGC
hsa-miR-644	4	AGUGUGGCUUUCUUAGAGC	GUGUGG
hsa-miR-642	4	GUCCCUCUCCAAAUGUGUCUUG	UCCCUC
hsa-miR-640	4	AUGAUCCAGGAACCUGCCUCU	UGAUCC
hsa-miR-637	4	ACUGGGGGCUUUCGGGCUCUGCGU	CUGGGG
hsa-miR-635	4	ACUUGGGCACUGAAACAAUGUCC	CUUGGG
hsa-miR-634	4	AACCAGCACCCCAACUUUGGAC	ACCAGC
hsa-miR-631	4	AGACCUGGCCCAGACCUCAGC	GACCUG
hsa-miR-629*	4	GUUCUCCCAACGUAAGCCCAGC	UUCUCC
hsa-miR-628-5p	4	AUGCUGACAUAUUUACUAGAGG	UGCUGA
hsa-miR-624*	4	UAGUACCAGUACCUUGUGUUCA	AGUACC
hsa-miR-624	4	CACAAGGUAUUGGUAUUACCU	ACAAGG
hsa-miR-622	4	ACAGUCUGCUGAGGUUGGAGC	CAGUCU
hsa-miR-617	4	AGACUUCCCAUUUGAAGGUGGC	GACUUC

hsa-miR-612	4	GCUGGGCAGGGCUUCUGAGCUCCUU	CUGGGC
hsa-miR-601	4	UGGUCUAGGAUUGUUGGAGGAG	GGUCUA
hsa-miR-593*	4	AGGCACCAGCCAGGCAUUGCUCAGC	GGCACC
hsa-miR-593	4	UGUCUCUGCUGGGGUUUCU	GUCUCU
hsa-miR-592	4	UUGUGUCAAUAUGCGAUGAUGU	UGUGUC
hsa-miR-590	4	GAGCUUAUUCAUAAAAGUGCAG	AGCUUA
hsa-miR-589	4	UGAGAACCACGUCUGCUCUGAG	GAGAAC
hsa-miR-588	4	UUGGCCACAAUGGGUUAGAAC	UGGCCA
hsa-miR-582	4	UUACAGUUGUUCAACCAGUUACU	UACAGU
hsa-miR-581	4	UCUUGUGUUCUCUAGAUCAGU	CUUGUG
hsa-miR-575	4	GAGCCAGUUGGACAGGAGC	AGCCAG
hsa-miR-573	4	CUGAAGUGAUGUGUAACUGAUCAG	UGAAGU
hsa-miR-571	4	UGAGUUGGCCAUCUGAGUGAG	GAGUUG
hsa-miR-569	4	AGUUAAUGAAUCCUGGAAAGU	GUUAAU
hsa-miR-567	4	AGUAUGUUCUUCCAGGACAGAAC	GUAUGU
hsa-miR-566	4	GGGCGCCUGUGAUCCCAAC	GGCGCC
hsa-miR-564	4	AGGCACGGUGUCAGCAGGC	GGCACG
hsa-miR-562	4	AAAGUAGCUGUACCAUUUGC	AAGUAG
hsa-miR-558	4	UGAGCUGCUGUACCAAAAU	GAGCUG
hsa-miR-555	4	AGGGUAAGCUGAACCUCUGAU	GGGUAA
hsa-miR-552	4	AACAGGUGACUGGUUAGACAA	ACAGGU
hsa-miR-551b	4	GCGACCCAUACUUGGUUUCAG	CGACCC
hsa-miR-550	4	UGUCUUACUCCCUCAGGCACAU	GUCUUA
hsa-miR-548d	4	CAAAAACCACAGUUUCUUUUGC	AAAAAC
hsa-miR-548b-5p	4	AAAAGUAAUUGUGGUUUUGGCC	AAAGUA
hsa-miR-545	4	UCAGCAAACAUUUAUUGUGUGC	CAGCAA
hsa-miR-544	4	AUUCUGCAUUUUUAGCAAGUUC	UUCUGC
hsa-miR-542-3p	4	UGUGACAGAUUGAUAACUGAAA	GUGACA
hsa-miR-541	4	UGGUGGGCACAGAAUCUGGACU	GGUGGG
hsa-miR-532-3p	4	CCUCCCACACCCAAGGCUUGCA	CUCCCA
hsa-miR-532	4	CAUGCCUUGAGUGUAGGACCGU	AUGCCU
hsa-miR-527	4	CUGCAAAGGGAAGCCCUUUC	UGCAAA
hsa-miR-526b*	4	GAAAGUGCUUCCUUUUAGAGGC	AAAGUG
hsa-miR-526b	4	CUCUUGAGGGAAGCACUUUCUGU	UCUUGA
hsa-miR-525	4	CUCCAGAGGGAUGCACUUUCU	UCCAGA
hsa-miR-523*	4	CUCUAGAGGGAAGCGCUUUCUG	UCUAGA
hsa-miR-522	4	AAAAUGGUUCCCUUUAGAGUGU	AAAUGG
hsa-miR-521	4	AACGCACUUCCCUUUAGAGUGU	ACGCAC
hsa-miR-520h	4	ACAAAGUGCUUCCCUUUAGAGU	CAAAGU
hsa-miR-520f	4	AAGUGCUUCCUUUUAGAGGGUU	AGUGCU
hsa-miR-520e	4	AAAGUGCUUCCUUUUUGAGGG	AAGUGC
hsa-miR-520d*	4	CUACAAAGGGAAGCCCUUUC	UACAAA
hsa-miR-520d	4	AAAGUGCUUCUCUUUGGUGGGU	AAGUGC
hsa-miR-520c	4	AAAGUGCUUCCUUUUAGAGGGU	AAGUGC
hsa-miR-520b	4	AAAGUGCUUCCUUUUAGAGGG	AAGUGC
hsa-miR-520a*	4	CUCCAGAGGGAAGUACUUUCU	UCCAGA
hsa-miR-520a	4	AAAGUGCUUCCCUUUGGACUGU	AAGUGC
hsa-miR-519e	4	AAGUGCCUCCUUUUAGAGUGUU	AGUGCC
hsa-miR-519c	4	AAAGUGCAUCUUUUUAGAGGAU	AAGUGC
hsa-miR-519b-5p	4	CUCUAGAGGGAAGCGCUUUCUG	UCUAGA
hsa-miR-519b	4	AAAGUGCAUCCUUUUAGAGGUU	AAGUGC
hsa-miR-519a*	4	CUCUAGAGGGAAGCGCUUUCUG	UCUAGA
hsa-miR-519a	4	AAAGUGCAUCCUUUUAGAGUGU	AAGUGC
hsa-miR-518e*	4	CUCUAGAGGGAAGCGCUUUCUG	UCUAGA
hsa-miR-518a-5p	4	CUGCAAAGGGAAGCCCUUUC	UGCAAA
hsa-miR-517c	4	AUCGUGCAUCCUUUUAGAGUGU	UCGUGC

hsa-miR-517a	4	AUCGUGCAUCCCUUUAGAGUGU	UCGUGC
hsa-miR-516-5p	4	AUCUGGAGGUAAGAAGCACUUU	UCUGGA
hsa-miR-516-3p	4	UGCUUCCUUUCAGAGGGU	GCUUCC
hsa-miR-515-3p	4	GAGUGCCUUCUUUUGGAGCGUU	AGUGCC
hsa-miR-514	4	AUUGACACUUCUGUGAGUAGA	UUGACA
hsa-miR-513a-3p	4	UAAAUUUCACCUUUCUGAGAAGG	AAAUUU
hsa-miR-513	4	UUCACAGGGAGGUGUCAU	UCACAG
hsa-miR-512-3p	4	AAGUGCUGUCAUAGCUGAGGUC	AGUGCU
hsa-miR-511	4	GUGUCUUUUGCUCUGCAGUCA	UGUCUU
hsa-miR-509-5p	4	UACUGCAGACAGUGGCAAUCA	ACUGCA
hsa-miR-509-3p	4	UGAUUGGUACGUCUGUGGGUAG	GAUUGG
hsa-miR-509	4	UGAUUGGUACGUCUGUGGGUAG	GAUUGG
hsa-miR-499-3p	4	AACAUCACAGCAAGUCUGUGCU	ACAUCA
hsa-miR-497	4	CAGCAGCACACUGUGGUUUGU	AGCAGC
hsa-miR-496	4	UGAGUAUUACAUGGCCAAUCUC	GAGUAU
hsa-miR-493-3p	4	UGAAGGUCUACUGUGUGCCAGG	GAAGGU
hsa-miR-493	4	UUGUACAUGGUAGGCUUUCAUU	UGUACA
hsa-miR-491-3p	4	CUUAUGCAAGAUUCCCUUCUAC	UUAUGC
hsa-miR-491	4	AGUGGGGAACCCUUCCAUGAGG	GUGGGG
hsa-miR-490-5p	4	CCAUGGAUCUCCAGGUGGGU	CAUGGA
hsa-miR-489	4	GUGACAUCACAUAUACGGCAGC	UGACAU
hsa-miR-486	4	UCCUGUACUGAGCUGCCCCGAG	CCUGUA
hsa-miR-483-5p	4	AAGACGGGAGGAAAGAAGGGAG	AGACGG
hsa-miR-454-3p	4	UAGUGCAAUAUUGCUUAUAGGGU	AGUGCA
hsa-miR-453	4	AGGUUGUCCGUGGUGAGUUCGCA	GGUUGU
hsa-miR-452*	4	CUCAUCUGCAAAGAAGUAAGUG	UCAUCU
hsa-miR-450	4	UUUUGCGAUGUGUUCCUAAUAU	UUUGCG
hsa-miR-449b	4	AGGCAGUGUAUUGUUAGCUGGC	GGCAGU
hsa-miR-449	4	UGGCAGUGUAUUGUUAGCUGGU	GGCAGU
hsa-miR-433	4	AUCAUGAUGGGCUCCUCGGUGU	UCAUGA
hsa-miR-422a	4	ACUGGACUUAGGGUCAGAAGGC	CUGGAC
hsa-miR-421	4	AUCAACAGACAUUAAUUGGGCGC	UCAACA
hsa-miR-383	4	AGAUCAGAAGGUGAUUGUGGCU	GAUCAG
hsa-miR-376a	4	AUCAUAGAGGAAAAUCCACGU	UCAUAG
hsa-miR-374b*	4	CUUAGCAGGUUGUAUUAUCAUU	UUAGCA
hsa-miR-373	4	GAAGUGCUUCGAUUUUGGGGUGU	AAGUGC
hsa-miR-372	4	AAAGUGCUGCGACAUUUGAGCGU	AAGUGC
hsa-miR-371	4	AAGUGCCGCCAUCUUUUGAGUGU	AGUGCC
hsa-miR-365	4	UAAUGCCCCUAAAAAUCCUUAU	AAUGCC
hsa-miR-34c-3p	4	AAUCACUAACCACACGGCCAGG	AUCACU
hsa-miR-34b*	4	UAGGCAGUGUCAUUAGCUGAUUG	AGGCAG
hsa-miR-34a*	4	CAAUCAGCAAGUAUACUGCCCU	AAUCAG
hsa-miR-34a	4	UGGCAGUGUCUUAGCUGGUUGU	GGCAGU
hsa-miR-346	4	UGUCUGCCCGCAUGCCUGCCUCU	GUCUGC
hsa-miR-342-5p	4	AGGGGUGCUAUCUGUGAUUGA	GGGGUG
hsa-miR-342	4	UCUCACACAGAAAUCGCACCCGU	CUCACA
hsa-miR-339-3p	4	UGAGCGCCUCGACGACAGAGCCG	GAGCGC
hsa-miR-338	4	UCCAGCAUCAGUGAUUUUGUUG	CCAGCA
hsa-miR-337	4	CUCCUAUAUGAUGCCUUUCUUC	UCCUAU
hsa-miR-329	4	AACACACCUGGUUAACCUCUUU	ACACAC
hsa-miR-326	4	CCUCUGGGCCCUUCCUCCAG	CUCUGG
hsa-miR-323-5p	4	AGGUGGUCCGUGGCGCGUUCGC	GGUGGU
hsa-miR-323	4	CACAUUACACGGUCGACCUCU	ACAUUA
hsa-miR-32*	4	CAAUUUAGUGUGUGUGAUAUUU	AAUUUA
hsa-miR-31	4	AGGCAAGAUGCUGGCAUAGCU	GGCAAG
hsa-miR-30e-3p	4	CUUUCAGUCGGAUGUUUACAGC	UUUCAG

hsa-miR-30b*	4	CUGGGAGGUGGAUGUUUACUUC	UGGGAG
hsa-miR-30b	4	UGUAAACAUCCUACACUCAGCU	GUAAAC
hsa-miR-302a	4	UAAGUGCUUCCAUGUUUUGGUGA	AAGUGC
hsa-miR-29c	4	UAGCACCAUUUGAAAUCGGUUA	AGCACC
hsa-miR-299-5p	4	UGGUUUACCGUCCCACAUACAU	GGUUUA
hsa-miR-299-3p	4	UAUGUGGGAUGGUAAACCGCUU	AUGUGG
hsa-miR-297	4	AUGUAUGUGUGCAUGUGCAUG	UGUAUG
hsa-miR-27a*	4	AGGGCUUAGCUGCUUGUGAGCA	GGGCUU
hsa-miR-26a	4	UUCAAGUAAUCCAGGAUAGGCU	UCAAGU
hsa-miR-223*	4	CGUGUAUUUGACAAGCUGAGUU	GUGUAU
hsa-miR-223	4	UGUCAGUUUGUCAAAUACCCCA	GUCAGU
hsa-miR-222*	4	CUCAGUAGCCAGUGUAGAUCCU	UCAGUA
hsa-miR-221*	4	ACCUGGCAUACAAUGUAGAUUU	CCUGGC
hsa-miR-220c	4	ACACAGGGCUGUUGUGAAGACU	CACAGG
hsa-miR-218-1*	4	AUGGUUCCGUCAAGCACCAUGG	UGGUUC
hsa-miR-218	4	UUGUGCUUGAUCUAACCAUGU	UGUGCU
hsa-miR-215	4	AUGACCUAUGAAUUGACAGAC	UGACCU
hsa-miR-212	4	UAACAGUCUCCAGUCACGGCC	AACAGU
hsa-miR-211	4	UUCCCUUUGUCAUCCUUCGCCU	UCCCUU
hsa-miR-20a	4	UAAAGUGCUUAUAGUGCAGGUAG	AAAGUG
hsa-miR-208b	4	AUAAGACGAACAAAAGGUUUGU	UAAGAC
hsa-miR-206	4	UGGAAUGUAAGGAAGUGUGUGG	GGAAUG
hsa-miR-202	4	AGAGGUAUAGGGCAUGGGAA	GAGGUA
hsa-miR-200c	4	UAAUACUGCCGGGUAAUGAUGGA	AAUACU
hsa-miR-200b	4	UAAUACUGCCUGGUAAUGAUGA	AAUACU
hsa-miR-200a	4	UAACACUGUCUGGUAACGAUGU	AACACU
hsa-miR-19b-1*	4	AGUUUUGCAGGUUUGCAUCCAGC	GUUUUG
hsa-miR-199b-3p	4	ACAGUAGUCUGCACAUUGGUUA	CAGUAG
hsa-miR-199a*	4	ACAGUAGUCUGCACAUUGGUUA	CAGUAG
hsa-miR-196a	4	UAGGUAGUUUCAUGUUGUUGGG	AGGUAG
hsa-miR-194	4	UGUAACAGCAACUCCAUGUGGA	GUAACA
hsa-miR-193b	4	AACUGGCCCUCAAAGUCCCGCU	ACUGGC
hsa-miR-193a	4	AACUGGCCUACAAAGUCCCAGU	ACUGGC
hsa-miR-192	4	CUGACCUAUGAAUUGACAGCC	UGACCU
hsa-miR-191	4	CAACGGAAUCCCAAAAGCAGCUG	AACGGA
hsa-miR-18b*	4	UGCCCUAAAUGCCCCUUCUGGC	GCCCUA
hsa-miR-18b	4	UAAGGUGCAUCUAGUGCAGUUAG	AAGGUG
hsa-miR-18a	4	UAAGGUGCAUCUAGUGCAGAUAG	AAGGUG
hsa-miR-189	4	UGCCUACUGAGCUGAUAUCAGU	GCCUAC
hsa-miR-185	4	UGGAGAGAAAGGCAGUUCCUGA	GGAGAG
hsa-miR-182	4	UUUGGCAAUGGUAGAACUCACACU	UUGGCA
hsa-miR-181c	4	AACAUUCAACCUGUCGGUGAGU	ACAUUC
hsa-miR-17-3p	4	ACUGCAGUGAAGGCACUUGUAG	CUGCAG
hsa-miR-16	4	UAGCAGCACGUAAAUAUUGGCG	AGCAGC
hsa-miR-15a*	4	CAGGCCAUAUUGUGCUGCCUCA	AGGCCA
hsa-miR-155	4	UUAAUGCUAAUCGUGAUAGGGGU	UAAUGC
hsa-miR-154	4	UAGGUUAUCCGUGUUGCCUUCG	AGGUUA
hsa-miR-152	4	UCAGUGCAUGACAGAACUUGG	CAGUGC
hsa-miR-151-5p	4	UCGAGGAGCUCACAGUCUAGU	CGAGGA
hsa-miR-151	4	CUAGACUGAAGCUCCUUGAGG	UAGACU
hsa-miR-149*	4	AGGGAGGGACGGGGGCUGUGC	GGGAGG
hsa-miR-148b*	4	AAGUUCUGUUAUACACUCAGGC	AGUUCU
hsa-miR-148a	4	UCAGUGCACUACAGAACUUUGU	CAGUGC
hsa-miR-147b	4	GUGUGCGGAAAUGCUUCUGCUA	UGUGCG
hsa-miR-147	4	GUGUGUGGAAAUGCUUCUGC	UGUGUG
hsa-miR-146b-3p	4	UGCCCUGUGGACUCAGUUCUGG	GCCCUG

hsa-miR-146a*	4	CCUCUGAAAUUCAGUUCUUCAG	CUCUGA
hsa-miR-145*	4	GGAUUCCUGGAAAUACUGUUCU	GAUUCC
hsa-miR-144*	4	GGAUAUCAUCAUAUACUGUAAG	GAUAUC
hsa-miR-143	4	UGAGAUGAAGCACUGUAGCUC	GAGAUG
hsa-miR-138-2*	4	GCUAUUUCACGACACCAGGGUU	CUAUUU
hsa-miR-138	4	AGCUGGUGUUGUGAAUCAGGCCG	GCUGGU
hsa-miR-137	4	UUAUUGCUUAAGAAUACGCGUAG	UAUUGC
hsa-miR-135b*	4	AUGUAGGGCUAAAAGCCAUGGG	UGUAGG
hsa-miR-135a	4	UAUGGCUUUUUAUUCCUAUGUGA	AUGGCU
hsa-miR-133b	4	UUUGGUCCCCUUCAACCAGCUA	UUGGUC
hsa-miR-133a	4	UUUGGUCCCCUUCAACCAGCUG	UUGGUC
hsa-miR-132*	4	ACCGUGGCUUUCGAUUGUUACU	CCGUGG
hsa-miR-132	4	UAACAGUCUACAGCCAUGGUCG	AACAGU
hsa-miR-130a*	4	UUCACAUUGUGCUACUGUCUGC	UCACAU
hsa-miR-129	4	CUUUUUGCGGUCUGGGCUUGC	UUUUUG
hsa-miR-128	4	UCACAGUGAACCGGUCUCUUU	CACAGU
hsa-miR-127-5p	4	CUGAAGCUCAGAGGGCUCUGAU	UGAAGC
hsa-miR-126	4	UCGUACCGUGAGUAAUAAUGCG	CGUACC
hsa-miR-124*	4	CGUGUUCACAGCGGACCUUGAU	GUGUUC
hsa-miR-1233	4	UGAGCCCUGUCCUCCCGCAG	GAGCCC
hsa-miR-1229	4	CUCUCACCACUGCCCUCCCACAG	UCUCAC
hsa-miR-1228	4	UCACACCUGCCUCGCCCCCC	CACACC
hsa-miR-1227	4	CGUGCCACCCUUUUCCCCAG	GUGCCA
hsa-miR-105*	4	ACGGAUGUUUGAGCAUGUGCUA	CGGAUG
hsa-miR-101*	4	CAGUUAUCACAGUGCUGAUGCU	AGUUAU
hsa-miR-1	4	UGGAAUGUAAAGAAGUAUGUAU	GGAAUG
hsa-let-7i*	4	CUGCGCAAGCUACUGCCUUGCU	UGCGCA
hsa-let-7g*	4	CUGUACAGGCCACUGCCUUGC	UGUACA
hsa-let-7f-2*	4	CUAUACAGUCUACUGUCUUUCC	UAUACA
hsa-let-7f-1*	4	CUAUACAAUCUAUUGCCUUCCC	UAUACA
hsa-let-7f	4	UGAGGUAGUAGAUUGUAUAGUU	GAGGUA
hsa-let-7c*	4	UAGAGUUACACCCUGGGAGUUA	AGAGUU
hsa-miR-95	5	UUCAACGGGUAUUUAUUGAGCA	UCAACG
hsa-miR-765	5	UGGAGGAGAAGGAAGGUGAUG	GGAGGA
hsa-miR-455	5	UAUGUGCCUUUGGACUACAUCG	AUGUGC
hsa-miR-340*	5	UCCGUCUCAGUUACUUUAUAGC	CCGUCU
hsa-miR-24-2*	5	UGCCUACUGAGCUGAAACACAG	GCCUAC
hsa-miR-941	6	CACCCGGCUGUGUGCACAUGUGC	ACCCGG
hsa-miR-105	6	UCAAAUGCUCAGACUCCUGUGGU	CAAAUG
hsa-miR-937	7	AUCCGCGCUCUGACUCUCUGCC	UCCGCG
hsa-miR-510	7	UACUCAGGAGAGUGGCAAUCAC	ACUCAG
hsa-miR-361-3p	7	UCCCCCAGGUGUGAUUCUGAUUU	CCCCCA
hsa-miR-328	7	CUGGCCCUCUCUGCCCUUCCGU	UGGCCC
hsa-miR-136*	7	CAUCAUCGUCUCAAAUGAGUCU	AUCAUC
hsa-miR-125a	7	UCCCUGAGACCCUUUAACCUGUGA	CCCUGA
hsa-miR-1224-5p	7	GUGAGGACUCGGGAGGUGG	UGAGGA
hsa-miR-936	8	ACAGUAGAGGGAGGAAUCGCAG	CAGUAG
hsa-miR-935	8	CCAGUUACCGCUUCCGCUACCGC	CAGUUA
hsa-miR-876-5p	8	UGGAUUUCUUUGUGAAUCACCA	GGAUUU
hsa-miR-768-3p	8	UCACAAUGCUGACACUCAAACUGCUGAC	CACAAU
hsa-miR-621	8	GGCUAGCAACAGCGCUUACCU	GCUAGC
hsa-miR-520g	8	ACAAAGUGCUUCCCUUUAGAGUGU	CAAAGU
hsa-miR-518c*	8	UCUCUGGAGGGAAGCACUUUCUG	CUCUGG
hsa-miR-330-5p	8	UCUCUGGGCCUGUGUCUUAGGC	CUCUGG
hsa-miR-1226	8	UCACCAGCCCUGUGUUCCCUAG	CACCAG
hsa-miR-106b	8	UAAAGUGCUGACAGUGCAGAU	AAAGUG

hsa-miR-933	9	UGUGCGCAGGGAGACCUCUCCC	GUGCGC
hsa-miR-302d	9	UAAGUGCUUCCAUGUUUGAGUGU	AAGUGC
hsa-miR-101	9	UACAGUACUGUGAUAACUGAA	ACAGUA
hsa-miR-93	10	CAAAGUGCUGUUCGUGCAGGUAG	AAAGUG
hsa-miR-888	10	UACUCAAAAAGCUGUCAGUCA	ACUCAA
hsa-miR-654-3p	10	UAUGUCUGCUGACCAUCACCUU	AUGUCU
hsa-miR-490	10	CAACCUGGAGGACUCCAUGCUG	AACCUG
hsa-miR-219-1-3p	10	AGAGUUGAGUCUGGACGUCCCG	GAGUUG
hsa-miR-155*	10	CUCCUACAUAUUAGCAUUAACA	UCCUAC
hsa-miR-92b	11	UAUUGCACUCGUCCCGGCCUCC	AUUGCA
hsa-miR-653	11	GUGUUGAAACAAUCUCUACUG	UGUUGA
hsa-miR-520c-5p	11	CUCUAGAGGGAAGCACUUUCUG	UCUAGA
hsa-miR-338-5p	11	AACAAUAUCCUGGUGCUGAGUG	ACAAUA
hsa-miR-924	12	AGAGUCUUGUGAUGUCUUGC	GAGUCU
hsa-miR-9*	12	AUAAAGCUAGAUAACCGAAAGU	UAAAGC
hsa-miR-760	12	CGGCUCUGGGUCUGUGGGGA	GGCUCU
hsa-miR-627	12	GUGAGUCUCUAAGAAAAGAGGA	UGAGUC
hsa-miR-429	12	UAAUACUGUCUGGUAAAACCGU	AAUACU
hsa-miR-378	12	CUCCUGACUCCAGGUCCUGUGU	UCCUGA
hsa-miR-33b*	12	CAGUGCCUCGGCAGUGCAGCCC	AGUGCC
hsa-miR-331	12	GCCCCUGGGCCUAUCCUAGAA	CCCCUG
hsa-miR-23b	12	AUCACAUUGCCAGGGAUUACC	UCACAU
hsa-miR-217	12	UACUGCAUCAGGAACUGAUUGGA	ACUGCA
hsa-miR-210	12	CUGUGCGUGUGACAGCGGCUGA	UGUGCG
hsa-miR-204	12	UUCCCUUUGUCAUCCUAUGCCU	UCCCUU
hsa-miR-9	13	UCUUUGGUUAUCUAGCUGUAUGA	CUUUGG
hsa-miR-508-5p	13	UACUCCAGAGGGCGUCACUCAUG	ACUCCA
hsa-miR-502	13	AUCCUUGCUAUCUGGGUGCUA	UCCUUG
hsa-miR-141*	13	CAUCUUCCAGUACAGUGUUGGA	AUCUUC
hsa-miR-892a	14	CACUGUGUCCUUUCUGCGUAG	ACUGUG
hsa-miR-643	14	ACUUGUAUGCUAGCUCAGGUAG	CUUGUA
hsa-miR-587	14	UUUCCAUAGGUGAUGAGUCAC	UUCCAU
hsa-miR-494	14	UGAAACAUACACGGGAAACCUC	GAAACA
hsa-miR-484	14	UCAGGCUCAGUCCCCUCCCGAU	CAGGCU
hsa-miR-361	14	UUAUCAGAAUCUCCAGGGGUAC	UAUCAG
hsa-miR-28-3p	14	CACUAGAUUGUGAGCUCCUGGA	ACUAGA
hsa-miR-181d	14	AACAUUCAUUGUUGUCGGUGGGU	ACAUUC
hsa-miR-149	14	UCUGGCUCCGUGUCUUCACUCCC	CUGGCU
hsa-miR-148a*	14	AAAGUUCUGAGACACUCCGACU	AAGUUC
hsa-miR-140-3p	14	UACCACAGGGUAGAACCACGG	ACCACA
hsa-miR-106b*	14	CCGCACUGUGGGUACUUGCUGC	CGCACU
hsa-let-7c	14	UGAGGUAGUAGGUUGUAUGGUU	GAGGUA
hsa-miR-889	16	UUAAUAUCGGACAACCAUUGU	UAAUAU
hsa-miR-801	16	GAUUGCUCUGCGUGCGGAAUCGAC	AUUGCU
hsa-miR-502-3p	16	AAUGCACCUGGGCAAGGAUUCA	AUGCAC
hsa-miR-487	16	AAUCAUACAGGGACAUCCAGUU	AUCAUA
hsa-miR-485-3p	16	GUCAUACACGGCUCUCCUCUCU	UCAUAC
hsa-miR-483	16	UCACUCCUCUCCUCCCGUCUU	CACUCC
hsa-miR-32	16	UAUUGCACAUUACUAAGUUGCA	AUUGCA
hsa-miR-224	16	CAAGUCACUAGUGGUUCCGUU	AAGUCA
hsa-miR-220b	16	CCACCACCGUGUCUGACACUU	CACCAC
hsa-miR-219-2-3p	16	AGAAUUGUGGCUGGACAUCUGU	GAAUUG
hsa-miR-197	16	UUCACCACCUUCUCCACCCAGC	UCACCA
hsa-miR-181a-2*	16	ACCACUGACCGUUGACUGUACC	CCACUG
hsa-miR-887	18	GUGAACGGGCGCCAUCCCGAGG	UGAACG
hsa-miR-651	18	UUUAGGAUAAGCUUGACUUUUG	UUAGGA

hsa-miR-641	18	AAAGACAUAGGAUAGAGUCACCUC	AAGACA
hsa-miR-554	18	GCUAGUCCUGACUCAGCCAGU	CUAGUC
hsa-miR-411*	18	UAUGUAACACGGUCCACUAACC	AUGUAA
hsa-miR-410	18	AAUAUAACACAGAUGGCCUGU	AUAUAA
hsa-miR-369-5p	18	AGAUCGACCGUGUUAUAUUCGC	GAUCGA
hsa-miR-302c*	18	UUUAACAUGGGGGUACCUGCUG	UUAACA
hsa-miR-190b	18	UGAUAUGUUUGAUAUUGGGUU	GAUAUG
hsa-miR-886-3p	19	CGCGGGUGCUUACUGACCCUU	GCGGGU
hsa-miR-875-5p	19	UAUACCUCAGUUUUAUCAGGUG	AUACCU
hsa-miR-626	19	AGCUGUCUGAAAAUGUCUU	GCUGUC
hsa-miR-574	19	CACGCUCAUGCACACACCCACA	ACGCUC
hsa-miR-551a	19	GCGACCCACUCUUGGUUUCCA	CGACCC
hsa-miR-193a-5p	19	UGGGUCUUUGCGGGCGAGAUGA	GGGUCU
hsa-miR-146b	19	UGAGAACUGAAUUCCAUAGGCU	GAGAAC
hsa-miR-146a	19	UGAGAACUGAAUUCCAUGGGUU	GAGAAC
hsa-miR-140	19	CAGUGGUUUUACCCUAUGGUAG	AGUGGU
hsa-miR-876-3p	20	UGGUGGUUUACAAAGUAAUUCA	GGUGGU
hsa-miR-663	20	AGGCGGGGCGCCGCGGGACCGC	GGCGGG
hsa-miR-619	20	GACCUGGACAUGUUUGUGCCCAGU	ACCUGG
hsa-miR-539	20	GGAGAAAUUAUCCUUGGUGUGU	GAGAAA
hsa-miR-513c	20	UUCUCAAGGAGGUGUCGUUUAU	UCUCAA
hsa-miR-450b-3p	20	UUGGGAUCAUUUUGCAUCCAUA	UGGGAU
hsa-miR-33	20	GUGCAUUGUAGUUGCAUUGCA	UGCAUU
hsa-miR-128a	20	UCACAGUGAACCGGUCUCUUU	CACAGU
hsa-let-7e*	20	CUAUACGGCCUCCUAGCUUUCC	UAUACG
hsa-miR-874	21	CUGCCCUGGCCCGAGGGACCGA	UGCCCU
hsa-miR-648	21	AAGUGUGCAGGGCACUGGU	AGUGUG
hsa-miR-629	21	UGGGUUUACGUUGGGAGAACU	GGGUUU
hsa-miR-524	21	GAAGGCGCUUCCCUUUGGAGU	AAGGCG
hsa-miR-376a*	21	GUAGAUUCUCCUUCUAUGAGUA	UAGAUU
hsa-miR-29a	21	UAGCACCAUCUGAAAUCGGUUA	AGCACC
hsa-miR-222	21	AGCUACAUCUGGCUACUGGGU	GCUACA
hsa-miR-221	21	AGCUACAUUGUCUGCUGGGUUUC	GCUACA
hsa-miR-21*	21	CAACACCAGUCGAUGGGCUGU	AACACC
hsa-miR-20b	21	CAAAGUGCUCAUAGUGCAGGUAG	AAAGUG
hsa-miR-208	21	AUAAGACGAGCAAAAAGCUUGU	UAAGAC
hsa-miR-138-1*	21	GCUACUUCACAACACCAGGGCC	CUACUU
hsa-miR-770-5p	22	UCCAGUACCACGUGUCAGGGCCA	CCAGUA
hsa-miR-602	22	GACACGGGCGACAGCUGCGGCCC	ACACGG
hsa-miR-518a	22	GAAAGCGCUUCCCUUUGCUGGA	AAAGCG
hsa-miR-432	22	UCUUGGAGUAGGUCAUUGGGUGG	CUUGGA
hsa-miR-412	22	ACUUCACCUGGUCCACUAGCCGU	CUUCAC
hsa-miR-380-5p	22	UGGUUGACCAUAGAACAUGCGC	GGUUGA
hsa-miR-368	22	AACAUAGAGGAAAUUCCACGU	ACAUAG
hsa-miR-188-3p	22	CUCCCACAUGCAGGGUUUGCA	UCCCAC
hsa-miR-182*	22	UGGUUCUAGACUUGCCAACUA	GGUUCU
hsa-miR-10a*	22	CAAAUUCGUAUCUAGGGGAAUA	AAAUUC
hsa-miR-744*	23	CUGUUGCCACUAACCUCAACCU	UGUUGC
hsa-miR-618	23	AAACUCUACUUGUCCUUCUGAGU	AACUCU
hsa-miR-582-3p	23	UAACUGGUUGAACAACUGAACC	AACUGG
hsa-miR-556-3p	23	AUAUUACCAUUAGCUCAUCUUU	UAUUAC
hsa-miR-425-5p	23	AAUGACACGAUCACUCCCGUUGA	AUGACA
hsa-miR-33a*	23	CAAUGUUUCCACAGUGCAUCAC	AAUGUU
hsa-miR-30d*	23	CUUUCAGUCAGAUGUUUGCUGC	UUUCAG
hsa-miR-25	23	CAUUGCACUUGUCUCGGUCUGA	AUUGCA
hsa-miR-188	23	CAUCCCUUGCAUGGUGGAGGG	AUCCCU

hsa-miR-671	25	AGGAAGCCCUGGAGGGGCUGGAG	GGAAGC
hsa-miR-485-5p	25	AGAGGCUGGCCGUGAUGAAUUC	GAGGCU
hsa-miR-190	25	UGAUAUGUUUGAUAUAUUAGGU	GAUAUG
hsa-miR-187	25	UCGUGUCUUGUGUUGCAGCCGG	CGUGUC
hsa-miR-184	25	UGGACGGAGAACUGAUAAGGGU	GGACGG
hsa-miR-650	27	AGGAGGCAGCGCUCUCAGGAC	GGAGGC
hsa-miR-625	27	AGGGGGAAAGUUCUAUAGUCC	GGGGGA
hsa-miR-636	28	UGUGCUUGCUCGUCCCGCCCGCA	GUGCUU
hsa-miR-495	28	AAACAAACAUGGUGCACUUCUU	AACAAA
hsa-miR-25*	28	AGGCGGAGACUUGGGCAAUUG	GGCGGA
hsa-miR-196b	28	UAGGUAGUUUCCUGUUGUUGGG	AGGUAG
hsa-miR-129-3p	28	AAGCCCUUACCCCAAAAAGCAU	AGCCCU
hsa-miR-129*	28	AAGCCCUUACCCCAAAAAGUAU	AGCCCU
hsa-miR-122a	28	UGGAGUGUGACAAUGGUGUUUG	GGAGUG
hsa-miR-623	29	AUCCCUUGCAGGGGCUGUUGGGU	UCCCUU
hsa-miR-187*	29	GGCUACAACACAGGACCCGGGC	GCUACA
hsa-miR-613	30	AGGAAUGUUCCUUCUUUGCC	GGAAUG
hsa-miR-579	30	UUCAUUUGGUAUAAACCGCGAUU	UCAUUU
hsa-miR-585	35	UGGGCGUAUCUGUAUGCUA	GGGCGU
hsa-miR-576-3p	35	AAGAUGUGGAAAAAUUGGAAUC	AGAUGU
hsa-miR-486-3p	35	CGGGGCAGCUCAGUACAGGAU	GGGGCA
hsa-miR-409-3p	35	GAAUGUUGCUCGGUGAACCCCU	AAUGUU
hsa-miR-324-5p	35	CGCAUCCCCUAGGGCAUUGGUGU	GCAUCC
hsa-miR-195	35	UAGCAGCACAGAAAUAUUGGC	AGCAGC
hsa-miR-15a	35	UAGCAGCACAUAAUGGUUUGUG	AGCAGC
hsa-miR-584	36	UUAUGGUUUGCCUGGGACUGAG	UAUGGU
hsa-miR-144	36	UACAGUAUAGAUGAUGUACU	ACAGUA
hsa-miR-541*	38	AAAGGAUUCUGCUGUCGGUCCCACU	AAGGAU
hsa-miR-512-5p	38	CACUCAGCCUUGAGGGCACUUUC	ACUCAG
hsa-miR-422b	38	ACUGGACUUGGAGUCAGAAGG	CUGGAC
hsa-miR-302d*	38	ACUUUAACAUGGAGGCACUUGC	CUUUAA
hsa-miR-302b*	38	ACUUUAACAUGGAAGUGCUUUC	CUUUAA
hsa-miR-194*	38	CCAGUGGGGCUGCUGUUAUCUG	CAGUGG
hsa-miR-153	38	UUGCAUAGUCACAAAAGUGAUC	UGCAUA
hsa-miR-10b*	38	ACAGAUUCGAUUCUAGGGGAAU	CAGAUU
hsa-miR-517*	40	CCUCUAGAUGGAAGCACUGUCU	CUCUAG
hsa-miR-15b	40	UAGCAGCACAUCAUGGUUUACA	AGCAGC
hsa-miR-425	44	AUCGGGAAUGUCGUGUCCGCCC	UCGGGA
hsa-miR-34c	44	AGGCAGUGUAGUUAGCUGAUUGC	GGCAGU
hsa-miR-30c-2*	44	CUGGGAGAAGGCUGUUUACUCU	UGGGAG
hsa-miR-296	44	AGGGCCCCCCCUCAAUCCUGU	GGGCCC
hsa-miR-198	44	GGUCCAGAGGGGAGAUAGGUUC	GUCCAG
hsa-miR-150	44	UCUCCCAACCCUUGUACCAGUG	CUCCCA
hsa-miR-337-5p	46	GAACGGCUUCAUACAGGAGUU	AACGGC
hsa-miR-19b	46	UGUGCAAAUCCAUGCAAAACUGA	GUGCAA
hsa-miR-125b	59	UCCCUGAGACCCUAACUUGUGA	CCCUGA
hsa-let-7b*	59	CUAUACAACCUACUGCCUUCCC	UAUACA
hsa-let-7a*	59	CUAUACAAUCUACUGUCUUUC	UAUACA

Number of miRNAs	Number of groups
437	33
