# Supplementary material for: A Network-Based Method to Assess the Statistical Significance of Mild Co-Regulation Effects
Source: PLoS One. 2013 Sep 9;8(9):e73413. doi: 10.1371/journal.pone.0073413 (PMC3767771; doi:10.1371/journal.pone.0073413)
Supplement: Table S4 — List of miRNAs and their protein targets shown in Figure 8A . (PDF) [file pone.0073413.s006.pdf]

Group index	miRNA	Protein	Section
1	hsa-miR-517a	mTOR	I
1	hsa-miR-616	mTOR	I
1	hsa-miR-517b	mTOR	I
1	hsa-miR-517c	mTOR	I
1	hsa-miR-150*	mTOR	I
1	hsa-miR-100	mTOR	I
1	hsa-miR-127	mTOR	I
1	hsa-miR-34c-3p	mTOR	I
1	hsa-miR-515-5p	mTOR	I
1	hsa-miR-767-3p	mTOR	I
1	hsa-miR-99b	mTOR	I
1	hsa-miR-99a	mTOR	I
2	hsa-miR-922	GSK3B	I
2	hsa-miR-507	GSK3B	I
2	hsa-miR-24	GSK3B	I
2	hsa-miR-802	GSK3B	I
2	hsa-miR-214	GSK3B	I
2	hsa-miR-19b-1*	GSK3B	I
2	hsa-miR-215	GSK3B	I
2	hsa-miR-99a*	GSK3B	I
2	hsa-miR-452	GSK3B	I
2	hsa-miR-1231	GSK3B	I
2	hsa-miR-583	GSK3B	I
2	hsa-miR-632	GSK3B	I
2	hsa-miR-212	GSK3B	I
3	hsa-miR-455	CDK2	I
3	hsa-miR-18a	CDK2	I
3	hsa-miR-765	CDK2	I
3	hsa-miR-938	CDK2	I
3	hsa-miR-521	CDK2	I
3	hsa-miR-340*	CDK2	I
3	hsa-miR-196a*	CDK2	I
3	hsa-miR-610	CDK2	I
4	hsa-miR-621	ERK2	I
4	hsa-miR-520g	ERK2	I
4	hsa-miR-936	ERK2	I
4	hsa-miR-768-3p	ERK2	I
4	hsa-miR-1226	ERK2	I
4	hsa-miR-330-5p	ERK2	I
4	hsa-miR-584	ERK2	I
4	hsa-miR-518c*	ERK2	I
4	hsa-miR-876-5p	ERK2	I
4	hsa-miR-106b	ERK2	I
4	hsa-miR-935	ERK2	I
5	hsa-miR-491-3p	Cyclin_D1	I
5	hsa-miR-888	Cyclin_D1	I
5	hsa-miR-490	Cyclin_D1	I
5	hsa-miR-1228	Cyclin_D1	I
5	hsa-miR-219-1-3p	Cyclin_D1	I

5	hsa-miR-155*	Cyclin_D1	I
5	hsa-miR-93	Cyclin_D1	I
6	hsa-miR-92b	KRAS	I
6	hsa-miR-545	KRAS	I
6	hsa-miR-575	KRAS	I
6	hsa-miR-520h	KRAS	I
6	hsa-miR-520c-5p	KRAS	I
7	hsa-miR-627	PLCG1	I
7	hsa-miR-211	PLCG1	I
7	hsa-miR-924	PLCG1	I
7	hsa-miR-378	PLCG1	I
7	hsa-miR-429	PLCG1	I
7	hsa-miR-760	PLCG1	I
7	hsa-miR-33b*	PLCG1	I
7	hsa-miR-331	PLCG1	I
8	hsa-miR-921	MIG_6	I
8	hsa-miR-376a	MIG_6	I
8	hsa-miR-554	MIG_6	I
8	hsa-miR-369-5p	MIG_6	I
8	hsa-miR-103	MIG_6	I
9	hsa-miR-141*	SHC1	I
9	hsa-miR-9	SHC1	I
9	hsa-miR-502	SHC1	I
9	hsa-miR-133b	SHC1	I
10	hsa-let-7a*	DUSP6	I
10	hsa-miR-892a	DUSP6	I
10	hsa-miR-106b*	DUSP6	I
10	hsa-miR-28-3p	DUSP6	I
10	hsa-miR-181a	DUSP6	I
10	hsa-miR-199a	DUSP6	I
10	hsa-miR-148a*	DUSP6	I
11	hsa-miR-502-3p	JNK1	I
11	hsa-miR-224	JNK1	I
11	hsa-miR-32	JNK1	I
11	hsa-miR-197	JNK1	I
11	hsa-miR-181a-2*	JNK1	I
11	hsa-miR-487	JNK1	I
11	hsa-miR-889	JNK1	I
12	hsa-miR-551b	PIK3CA	I
12	hsa-miR-888*	PIK3CA	I
12	hsa-miR-526b	PIK3CA	I
12	hsa-miR-526b*	PIK3CA	I
13	hsa-miR-193a-5p	EGFR	I
13	hsa-miR-140	EGFR	I
13	hsa-miR-146b	EGFR	I
13	hsa-miR-146a	EGFR	I
13	hsa-miR-574	EGFR	I
13	hsa-miR-886-3p	EGFR	I
13	hsa-miR-579	EGFR	I
13	hsa-miR-493	EGFR	I

13	hsa-miR-135a	EGFR	I
13	hsa-miR-206	EGFR	I
13	hsa-miR-626	EGFR	I
14	hsa-let-7e*	p38	I
14	hsa-miR-128	p38	I
14	hsa-miR-619	p38	I
14	hsa-miR-452*	p38	I
14	hsa-miR-876-3p	p38	I
14	hsa-miR-450b-3p	p38	I
14	hsa-miR-18b*	p38	I
15	hsa-miR-376a*	STAT3	I
15	hsa-miR-202*	STAT3	I
15	hsa-miR-138-1*	STAT3	I
15	hsa-miR-298	STAT3	I
15	hsa-miR-21*	STAT3	I
15	hsa-miR-516-5p	STAT3	I
15	hsa-miR-665	STAT3	I
15	hsa-miR-29a	STAT3	I
15	hsa-miR-222	STAT3	I
15	hsa-miR-874	STAT3	I
15	hsa-miR-612	STAT3	I
15	hsa-miR-532-3p	STAT3	I
15	hsa-miR-221	STAT3	I
15	hsa-miR-648	STAT3	I
15	hsa-miR-29c	STAT3	I
15	hsa-miR-15a*	STAT3	I
16	hsa-miR-302d	AKT1	I
16	hsa-miR-516-3p	AKT1	I
16	hsa-miR-768-5p	AKT1	I
16	hsa-miR-373	AKT1	I
16	hsa-miR-101	AKT1	I
17	hsa-miR-19b	PTEN	I
17	hsa-miR-1227	PTEN	I
17	hsa-miR-582-3p	PTEN	I
17	hsa-miR-188	PTEN	I
17	hsa-miR-519e	PTEN	I
17	hsa-miR-30e-3p	PTEN	I
17	hsa-miR-25	PTEN	I
17	hsa-miR-744*	PTEN	I
17	hsa-miR-556-3p	PTEN	I
17	hsa-miR-371	PTEN	I
18	hsa-miR-495	ERK1	I
18	hsa-miR-636	ERK1	I
18	hsa-miR-185	ERK1	I
18	hsa-miR-222*	ERK1	I
18	hsa-miR-122a	ERK1	I
18	hsa-miR-431*	ERK1	I
18	hsa-miR-196b	ERK1	I
18	hsa-miR-27a*	ERK1	I
18	hsa-miR-671-3p	ERK1	I

18	hsa-miR-129-3p	ERK1	I
19	hsa-miR-671	AKT2	I
19	hsa-miR-151-5p	AKT2	I
19	hsa-miR-485-5p	AKT2	I
19	hsa-miR-564	AKT2	I
19	hsa-miR-187	AKT2	I
19	hsa-miR-184	AKT2	I
19	hsa-miR-650	AKT2	I
19	hsa-miR-625	AKT2	I
20	hsa-miR-527	CDK4	I
20	hsa-miR-149*	CDK4	I
20	hsa-miR-425	CDK4	I
20	hsa-miR-628-5p	CDK4	I
20	hsa-miR-198	CDK4	I
20	hsa-miR-34c	CDK4	I
21	hsa-miR-328	PTPN11	I
21	hsa-miR-130a*	PTPN11	I
21	hsa-miR-601	PTPN11	I
21	hsa-miR-135b*	PTPN11	I
21	hsa-miR-605	PTPN11	I
21	hsa-miR-136*	PTPN11	I
21	hsa-miR-361-3p	PTPN11	I
21	hsa-miR-509-3-5p	PTPN11	I
21	hsa-miR-510	PTPN11	I
22	hsa-miR-223	Cyclin_D3	I
22	hsa-miR-15a	Cyclin_D3	I
22	hsa-miR-422a	Cyclin_D3	I
22	hsa-miR-589	Cyclin_D3	I
23	hsa-miR-223*	GRB2	I
23	hsa-miR-221*	GRB2	I
23	hsa-miR-200a	GRB2	I
23	hsa-miR-567	GRB2	I
23	hsa-miR-422b	GRB2	I
23	hsa-miR-194*	GRB2	I
23	hsa-miR-146b-3p	GRB2	I
23	hsa-miR-302d*	GRB2	I
23	hsa-miR-512-5p	GRB2	I
23	hsa-miR-363*	GRB2	I
23	hsa-miR-132*	GRB2	I
24	hsa-miR-15b	p27_Kip1	I
24	hsa-miR-517*	p27_Kip1	I
25	hsa-miR-380-5p	PIK3CB	I
25	hsa-miR-412	PIK3CB	I
26	hsa-miR-365	RB1	I
26	hsa-miR-26a-2*	RB1	I
27	hsa-miR-552	Cyclin_D3	II
27	hsa-miR-552	GRB2	II
27	hsa-miR-297	GRB2	II
27	hsa-miR-297	Cyclin_D3	II
27	hsa-miR-744	Cyclin_D3	II

27	hsa-miR-744	GRB2	II
28	hsa-miR-520d*	KRAS	II
28	hsa-miR-520d*	EGFR	II
28	hsa-miR-661	KRAS	II
28	hsa-miR-661	EGFR	II
29	hsa-miR-588	mTOR	II
29	hsa-miR-588	Cyclin_D3	II
29	hsa-miR-635	mTOR	II
29	hsa-miR-635	Cyclin_D3	II
30	hsa-miR-623	CDK2	II
30	hsa-miR-623	STAT3	II
30	hsa-miR-187*	CDK2	II
30	hsa-miR-187*	STAT3	II
31	hsa-miR-148b*	mTOR	II
31	hsa-miR-148b*	GRB2	II
31	hsa-miR-581	mTOR	II
31	hsa-miR-581	GRB2	II
32	hsa-miR-191	PIK3CB	II
32	hsa-miR-191	Cyclin_D3	II
32	hsa-miR-450	Cyclin_D3	II
32	hsa-miR-450	PIK3CB	II
33	hsa-miR-96	SHC1	II
33	hsa-miR-96	mTOR	II
33	hsa-miR-202	SHC1	II
33	hsa-miR-202	ERK1	II
33	hsa-miR-202	mTOR	II
34	hsa-miR-496	GSK3B	II
34	hsa-miR-496	mTOR	II
34	hsa-miR-490-5p	AKT2	II
34	hsa-miR-490-5p	GSK3B	II
34	hsa-miR-490-5p	mTOR	II
34	hsa-miR-523*	GSK3B	II
34	hsa-miR-523*	mTOR	II
35	hsa-miR-520f	ERK2	II
35	hsa-miR-520f	p27_Kip1	II
35	hsa-miR-515-3p	ERK2	II
35	hsa-miR-515-3p	p27_Kip1	II
35	hsa-miR-515-3p	PTEN	II
35	hsa-miR-515-3p	PTEN	II
36	hsa-miR-342	KRAS	II
36	hsa-miR-342	GSK3B	II
36	hsa-miR-342	MIG_6	II
36	hsa-miR-192	KRAS	II
36	hsa-miR-192	GSK3B	II
37	hsa-miR-147b	mTOR	II
37	hsa-miR-147b	MIG_6	II
37	hsa-miR-154	MIG_6	II
37	hsa-miR-154	mTOR	II
37	hsa-miR-154	AKT1	II
38	hsa-miR-590	KRAS	II

38	hsa-miR-590	STAT3	II
38	hsa-miR-593*	GRB2	II
38	hsa-miR-593*	KRAS	II
38	hsa-miR-593*	STAT3	II
38	hsa-miR-593*	Cyclin_D1	II
39	hsa-miR-550	PIK3CA	II
39	hsa-miR-550	p38	II
39	hsa-miR-550	PTPN11	II
39	hsa-miR-200c	PIK3CA	II
39	hsa-miR-200c	PLCG1	II
39	hsa-miR-200c	PTPN11	II
39	hsa-miR-522	PIK3CA	II
39	hsa-miR-522	PTPN11	II
39	hsa-miR-489	PTPN11	II
39	hsa-miR-489	p38	II
39	hsa-miR-200b	PIK3CA	II
39	hsa-miR-200b	PLCG1	II
40	hsa-miR-519c	ERK2	II
40	hsa-miR-519c	PTEN	II
40	hsa-miR-519c	PIK3CA	II
40	hsa-miR-519c	ERK1	II
40	hsa-miR-483-5p	PTPN11	II
40	hsa-miR-483-5p	GRB2	II
40	hsa-miR-483-5p	ERK1	II
40	hsa-miR-483-5p	PIK3CA	II
40	hsa-miR-483-5p	RB1	II
40	hsa-miR-483-5p	ERK2	II
41	hsa-miR-491	AKT2	II
41	hsa-miR-491	CDK4	II
41	hsa-miR-491	PTPN11	II
41	hsa-miR-491	ERK1	II
41	hsa-miR-491	mTOR	II
41	hsa-miR-491	EGFR	II
41	hsa-miR-592	mTOR	II
41	hsa-miR-592	EGFR	II
41	hsa-miR-592	PTPN11	II
41	hsa-miR-544	mTOR	II
41	hsa-miR-544	EGFR	II
41	hsa-miR-127-5p	EGFR	II
41	hsa-miR-127-5p	PIK3CA	II
41	hsa-miR-127-5p	Cyclin_D1	II
41	hsa-miR-127-5p	mTOR	II
41	hsa-miR-421	PTPN11	II
41	hsa-miR-421	EGFR	II
41	hsa-miR-421	mTOR	II
42	hsa-let-7c*	GSK3B	III
42	hsa-let-7c*	SHC1	III
42	hsa-miR-520a	ERK2	III
42	hsa-miR-520a	CDK4	III
42	hsa-miR-520a	PIK3CA	III

42	hsa-miR-520a	AKT1	III
42	hsa-let-7f-1*	p38	III
42	hsa-let-7f-1*	GSK3B	III
42	hsa-let-7f	Cyclin_D3	III
42	hsa-let-7f	RB1	III
42	hsa-let-7f	AKT1	III
42	hsa-let-7f	Cyclin_D1	III
42	hsa-let-7g*	GSK3B	III
42	hsa-let-7g*	EGFR	III
42	hsa-let-7i*	AKT1	III
42	hsa-let-7i*	PTEN	III
42	hsa-miR-640	ERK1	III
42	hsa-miR-640	Cyclin_D1	III
42	hsa-miR-640	GSK3B	III
42	hsa-miR-105*	JNK1	III
42	hsa-miR-105*	STAT3	III
42	hsa-miR-105*	STAT3	III
42	hsa-miR-199b-3p	MIG_6	III
42	hsa-miR-199b-3p	SHC1	III
42	hsa-miR-199b-3p	PIK3CA	III
42	hsa-miR-199b-3p	JNK1	III
42	hsa-let-7f-2*	GSK3B	III
42	hsa-let-7f-2*	JNK1	III
42	hsa-miR-433	GRB2	III
42	hsa-miR-433	p38	III
42	hsa-miR-433	JNK1	III
42	hsa-miR-126	AKT2	III
42	hsa-miR-126	EGFR	III
42	hsa-miR-642	AKT1	III
42	hsa-miR-642	Cyclin_D3	III
42	hsa-miR-642	PTEN	III
42	hsa-miR-449b	CDK4	III
42	hsa-miR-449b	PLCG1	III
42	hsa-miR-525	CDK4	III
42	hsa-miR-525	PLCG1	III
42	hsa-miR-525	RB1	III
42	hsa-miR-525	Cyclin_D3	III
42	hsa-miR-766	Cyclin_D3	III
42	hsa-miR-766	AKT1	III
42	hsa-miR-148a	PIK3CA	III
42	hsa-miR-148a	MIG_6	III
42	hsa-miR-132	MIG_6	III
42	hsa-miR-132	GSK3B	III
42	hsa-miR-132	ERK1	III
42	hsa-miR-132	PIK3CA	III
42	hsa-miR-132	RB1	III
42	hsa-miR-541	SHC1	III
42	hsa-miR-541	CDK4	III
42	hsa-miR-541	PIK3CA	III
42	hsa-miR-542-3p	Cyclin_D3	III

42	hsa-miR-542-3p	CDK2	III
42	hsa-miR-218-1*	Cyclin_D1	III
42	hsa-miR-218-1*	MIG_6	III
42	hsa-miR-218-1*	PIK3CA	III
42	hsa-miR-137	AKT2	III
42	hsa-miR-137	EGFR	III
42	hsa-miR-548d	CDK2	III
42	hsa-miR-548d	AKT1	III
42	hsa-miR-138-2*	Cyclin_D1	III
42	hsa-miR-138-2*	GSK3B	III
42	hsa-miR-138-2*	STAT3	III
42	hsa-miR-155	SHC1	III
42	hsa-miR-155	EGFR	III
42	hsa-miR-155	GSK3B	III
42	hsa-miR-329	CDK4	III
42	hsa-miR-329	PIK3CB	III
42	hsa-miR-329	RB1	III
42	hsa-miR-329	AKT2	III
42	hsa-miR-329	AKT2	III
42	hsa-miR-144*	AKT2	III
42	hsa-miR-144*	PIK3CA	III
42	hsa-miR-144*	PTPN11	III
42	hsa-miR-144*	RB1	III
42	hsa-miR-144*	SHC1	III
42	hsa-miR-144*	GSK3B	III
42	hsa-miR-520e	AKT1	III
42	hsa-miR-520e	ERK2	III
42	hsa-miR-145*	GRB2	III
42	hsa-miR-145*	STAT3	III
42	hsa-miR-145*	JNK1	III
42	hsa-miR-562	Cyclin_D1	III
42	hsa-miR-562	JNK1	III
42	hsa-miR-499-3p	Cyclin_D1	III
42	hsa-miR-499-3p	JNK1	III
42	hsa-miR-499-3p	PLCG1	III
42	hsa-miR-299-5p	AKT2	III
42	hsa-miR-299-5p	Cyclin_D3	III
42	hsa-miR-299-5p	RB1	III
42	hsa-miR-147	AKT2	III
42	hsa-miR-147	CDK2	III
42	hsa-miR-147	CDK4	III
42	hsa-miR-147	RB1	III
42	hsa-miR-147	Cyclin_D1	III
42	hsa-miR-302a	Cyclin_D3	III
42	hsa-miR-302a	CDK2	III
42	hsa-miR-302a	ERK2	III
42	hsa-miR-302a	PTEN	III
42	hsa-miR-302a	AKT1	III
42	hsa-miR-34a	PLCG1	III
42	hsa-miR-34a	CDK4	III

42	hsa-miR-34a	GSK3B	III
42	hsa-miR-34a*	DUSP6	III
42	hsa-miR-34a*	MIG_6	III
42	hsa-miR-509-3p	DUSP6	III
42	hsa-miR-509-3p	CDK2	III
42	hsa-miR-509-3p	CDK2	III
42	hsa-miR-509-3p	PTEN	III
42	hsa-miR-509-3p	PTEN	III
42	hsa-miR-30b*	AKT1	III
42	hsa-miR-30b*	CDK4	III
42	hsa-miR-30b*	PLCG1	III
42	hsa-miR-151	AKT1	III
42	hsa-miR-151	CDK4	III
42	hsa-miR-151	DUSP6	III
42	hsa-miR-151	EGFR	III
42	hsa-miR-151	GSK3B	III
42	hsa-miR-151	PTPN11	III
42	hsa-miR-323	STAT3	III
42	hsa-miR-323	JNK1	III
42	hsa-miR-323-5p	PIK3CA	III
42	hsa-miR-323-5p	RB1	III
42	hsa-miR-326	MIG_6	III
42	hsa-miR-326	PIK3CA	III
42	hsa-miR-193b	CDK4	III
42	hsa-miR-193b	RB1	III
42	hsa-miR-193b	CDK2	III
42	hsa-miR-16	Cyclin_D3	III
42	hsa-miR-16	ERK1	III
42	hsa-miR-16	Cyclin_D3	III
42	hsa-miR-16	CDK4	III
42	hsa-miR-16	RB1	III
42	hsa-miR-17-3p	AKT2	III
42	hsa-miR-17-3p	EGFR	III
42	hsa-miR-17-3p	PIK3CA	III
42	hsa-miR-17-3p	PIK3CB	III
42	hsa-miR-17-3p	RB1	III
42	hsa-miR-342-5p	CDK4	III
42	hsa-miR-342-5p	AKT2	III
42	hsa-miR-342-5p	PIK3CA	III
42	hsa-miR-181c	DUSP6	III
42	hsa-miR-181c	PIK3CA	III
42	hsa-miR-346	p38	III
42	hsa-miR-346	GSK3B	III
42	hsa-miR-34b*	CDK4	III
42	hsa-miR-34b*	GSK3B	III
42	hsa-miR-34b*	RB1	III
42	hsa-miR-18b	AKT1	III
42	hsa-miR-18b	ERK2	III
42	hsa-miR-631	GSK3B	III
42	hsa-miR-631	EGFR	III

42	hsa-miR-520a*	DUSP6	III
42	hsa-miR-520a*	JNK1	III
42	hsa-miR-520a*	PIK3CA	III
42	hsa-miR-193a	Cyclin_D1	III
42	hsa-miR-193a	CDK4	III
42	hsa-miR-193a	MIG_6	III
42	hsa-miR-193a	JNK1	III
42	hsa-miR-193a	PIK3CA	III
42	hsa-miR-520c	ERK2	III
42	hsa-miR-520c	AKT1	III
42	hsa-miR-637	STAT3	III
42	hsa-miR-637	p27_Kip1	III
42	hsa-miR-637	GRB2	III
42	hsa-miR-520d	ERK2	III
42	hsa-miR-520d	AKT1	III
42	hsa-miR-518a-5p	CDK4	III
42	hsa-miR-518a-5p	CDK4	III
42	hsa-miR-518a-5p	DUSP6	III
42	hsa-miR-518a-5p	PTPN11	III
42	hsa-miR-644	AKT2	III
42	hsa-miR-644	PIK3CA	III
42	hsa-miR-644	EGFR	III
42	hsa-miR-558	RB1	III
42	hsa-miR-558	Cyclin_D3	III
42	hsa-miR-558	CDK4	III
42	hsa-miR-519a	CDK2	III
42	hsa-miR-519a	AKT1	III
42	hsa-miR-519a	ERK1	III
42	hsa-miR-519a	PTEN	III
42	hsa-miR-519a	CDK2	III
42	hsa-miR-449	CDK4	III
42	hsa-miR-449	PLCG1	III
42	hsa-miR-524*	JNK1	III
42	hsa-miR-524*	EGFR	III
42	hsa-miR-524*	DUSP6	III
42	hsa-miR-509	PTEN	III
42	hsa-miR-509	CDK2	III
42	hsa-miR-519b-5p	RB1	III
42	hsa-miR-519b-5p	AKT2	III
42	hsa-miR-519b-5p	Cyclin_D1	III
42	hsa-miR-208b	JNK1	III
42	hsa-miR-208b	STAT3	III
42	hsa-miR-769-5p	GSK3B	III
42	hsa-miR-769-5p	SHC1	III
42	hsa-miR-769-5p	RB1	III
42	hsa-miR-769-5p	JNK1	III
42	hsa-miR-769-5p	MIG_6	III
42	hsa-miR-31	GRB2	III
42	hsa-miR-31	CDK2	III
42	hsa-miR-31	JNK1	III

42	hsa-miR-486	DUSP6	III
42	hsa-miR-486	MIG_6	III
42	hsa-miR-486	PIK3CA	III
42	hsa-miR-877*	Cyclin_D1	III
42	hsa-miR-877*	PLCG1	III
42	hsa-miR-877*	CDK4	III
42	hsa-miR-885-3p	AKT2	III
42	hsa-miR-885-3p	CDK4	III
42	hsa-miR-26a	PTEN	III
42	hsa-miR-26a	ERK1	III
42	hsa-miR-26a	PTEN	III
42	hsa-miR-892b	MIG_6	III
42	hsa-miR-892b	RB1	III
42	hsa-miR-892b	KRAS	III
42	hsa-miR-892b	PIK3CA	III
42	hsa-miR-892b	Cyclin_D1	III
42	hsa-miR-892b	CDK4	III
42	hsa-miR-892b	CDK2	III
42	hsa-miR-338	PIK3CA	III
42	hsa-miR-338	JNK1	III
42	hsa-miR-338	MIG_6	III
42	hsa-miR-519a*	RB1	III
42	hsa-miR-519a*	CDK2	III
42	hsa-miR-569	Cyclin_D1	III
42	hsa-miR-569	JNK1	III
42	hsa-miR-569	ERK2	III
42	hsa-miR-940	CDK4	III
42	hsa-miR-940	STAT3	III
42	hsa-miR-940	GSK3B	III
42	hsa-miR-624*	SHC1	III
42	hsa-miR-624*	GSK3B	III
42	hsa-miR-624*	AKT2	III
42	hsa-miR-624*	EGFR	III
42	hsa-miR-372	AKT1	III
42	hsa-miR-372	ERK2	III
42	hsa-miR-372	PTPN11	III
42	hsa-miR-582	JNK1	III
42	hsa-miR-582	GSK3B	III
42	hsa-miR-511	CDK2	III
42	hsa-miR-511	PTEN	III
42	hsa-miR-511	PTEN	III
42	hsa-miR-634	GSK3B	III
42	hsa-miR-634	STAT3	III
42	hsa-miR-634	GRB2	III
42	hsa-miR-520b	AKT1	III
42	hsa-miR-520b	PTEN	III
42	hsa-miR-520b	ERK2	III
42	hsa-miR-629*	GSK3B	III
42	hsa-miR-629*	p38	III
42	hsa-miR-629*	STAT3	III

42	hsa-miR-608	PIK3CA	III
42	hsa-miR-608	DUSP6	III
42	hsa-miR-608	PIK3CB	III
42	hsa-miR-608	Cyclin_D1	III
42	hsa-miR-532	Cyclin_D1	III
42	hsa-miR-532	CDK2	III
42	hsa-miR-519b	CDK2	III
42	hsa-miR-519b	ERK2	III
42	hsa-miR-518e*	CDK4	III
42	hsa-miR-518e*	PTPN11	III
42	hsa-miR-518e*	GSK3B	III
42	hsa-miR-566	STAT3	III
42	hsa-miR-566	CDK4	III
42	hsa-miR-555	RB1	III
42	hsa-miR-555	DUSP6	III
42	hsa-miR-555	Cyclin_D1	III
42	hsa-miR-624	RB1	III
42	hsa-miR-624	Cyclin_D1	III
