# Supplementary material for: A Network-Based Method to Assess the Statistical Significance of Mild Co-Regulation Effects
Source: PLoS One. 2013 Sep 9;8(9):e73413. doi: 10.1371/journal.pone.0073413 (PMC3767771; doi:10.1371/journal.pone.0073413)
Supplement: Table S5 — SICORE groups in which the precursor families are significantly over-represented for regulation stringency thresholds . (PDF) [file pone.0073413.s007.pdf]

group	family	group count	family count	family in group	family members	p.hyper	fdr.hyper	p.perm	τ_B
1	mir-99	11	5	2	hsa-miR-99a*<br>hsa-miR-99b*	9.23E-05	3.85E-03	0.009	1.64
2	mir-99	8	5	2	hsa-miR-100<br>hsa-miR-99b	3.16E-05	1.40E-03	0.001	1.64
18	mir-154	9	11	2	hsa-miR-369-5p<br>hsa-miR-410	7.37E-04	2.35E-02	0.027	1.64
19	mir-146	9	4	2	hsa-miR-146a<br>hsa-miR-146b	1.91E-05	8.79E-04	0	1.64
21	mir-221	12	4	2	hsa-miR-221<br>hsa-miR-222	4.98E-05	2.20E-03	0.001	1.64
28	mir-129	7	3	2	hsa-miR-129*<br>hsa-miR-129-3p	2.01E-06	9.72E-05	0	1.64
35	mir-15	7	5	2	hsa-miR-15a<br>hsa-miR-195	1.98E-05	9.07E-04	0.004	1.64
38	mir-302	8	5	2	hsa-miR-302b*<br>has-miR-302d*	3.16E-05	1.40E-03	0.001	1.64
59	let-7	3	9	2	hsa-let-7a*<br>hsa-let-7b*	4.82E-06	2.32E-04	0.001	1.64
1	mir-99	12	4	3	hsa-miR-100<br>hsa-miR-99a<br>hsa-miR-99b	7.96E-07	4.55E-05	0.001	1.96
5	let-7	90	7	5	hsa-let-7f<br>hsa-let-7f-1*<br>hsa-let-7f-2*<br>hsa-let-7g*<br>hsa-let-7i*	1.39E-03	2.17E-02	0.029	1.96
17	mir-146	11	3	2	hsa-miR-146a<br>hsa-miR-146b	2.31E-05	1.23E-03	0.005	1.96
19	mir-221	16	4	2	hsa-miR-221<br>hsa-miR-222	3.05E-04	1.47E-02	0.011	1.96
19	mir-29	16	2	2	hsa-miR-29a<br>hsa-miR-29c	0.00E+00	0.00E+00	0.001	1.96
33	mir-506	9	7	2	hsa-miR-509-3-5p<br>hsa-miR-510	3.91E-04	1.80E-02	0.018	1.96
42	mir-8	5	5	2	hsa-miR-200b<br>hsa-miR-200c	1.39E-05	7.87E-04	0.001	1.96
45	mir-515	2	61	2	hsa-miR-515-3p<br>hsa-miR-520f	0.00E+00	0.00E+00	0.021	1.96
1	mir-99	7	2	2	hsa-miR-100<br>hsa-miR-99a	0.00E+00	0.00E+00	0.003	2.58
4	mir-34	5	2	2	hsa-miR-34a<br>hsa-miR-34b*	0.00E+00	0.00E+00	0	2.58
7	mir-8	7	4	3	hsa-miR-200b<br>hsa-miR-200c<br>hsa-miR-429	1.40E-06	5.08E-05	0	2.58
13	mir-29	11	2	2	hsa-miR-29a<br>hsa-miR-29c	0.00E+00	0.00E+00	0.004	2.58
25	mir-146	11	2	2	hsa-miR-146a<br>hsa-miR-146b	0.00E+00	0.00E+00	0.011	2.58
45	mir-506	3	6	2	hsa-miR-509<br>hsa-miR-509-3p	3.10E-05	1.11E-03	0.005	2.58
