# Supplementary material for: A Network-Based Method to Assess the Statistical Significance of Mild Co-Regulation Effects
Source: PLoS One. 2013 Sep 9;8(9):e73413. doi: 10.1371/journal.pone.0073413 (PMC3767771; doi:10.1371/journal.pone.0073413)
Supplement: Text S1 — Graph definitions. (PDF) [file pone.0073413.s008.pdf]

Text S1 - Graph definitions

As exemplified on the specific biological data set used in this paper, we model the effect of a transfected miRNA on the observed protein levels by a bipartite graph with weighted edges. The bipartite graph consists of a set of nodes $V = M \cup P$ which contains the set M of miRNAs and the set P of proteins. For each pair of miRNA x and protein y, the edge $e = (x, y)$ between the two is associated with the observed positive or negative z-score. We call this the weight of the edge e. Note that there is no edge between any two proteins or any two miRNAs which defines the graph to be bipartite. In the given data set, there is an edge between any pair of miRNA or protein, i.e., the graph is *complete*. In general, a bipartite graph does not need to be complete, but the data can be reduced to a non-complete and unweighted bipartite graph by a thresholding step.

When the graph is weighted, it is described by a tupel $G = (M \cup P, E \subseteq (M \times P), \Omega)$ where $\Omega : E \to \mathbb{R}$ is a function which maps the edges to their weights. In the unweighted case, the graph is fully described by a tupel $G = (M \cup P, E)$.

Nodes that are connected by an edge are said to be *neighbours* or *adjacent* to each other. Let $n(v)$ denote the set of neighbours of v and let $deg(v) := |n(v)|$ denote the *degree* of node v. In a bipartite graph where v is a node in one of the two node sets, its neighbours are always from the other set. The *degree sequence* of a graph is the ordered sequence of the degrees of its nodes. For bipartite graphs, we have two degree sequences, one for each side of the graph. Given any bipartite graph and two nodes v, w from the same set, we denote as their co-occurrence $coocc(v, w)$ the number of common neighbours, i.e.:

$$coocc(v, w) = n(v) \cap n(w).$$
